# Mapping environmental suitability for *Pythium insidiosum* under current and future climate conditions

**DOI:** 10.1016/j.isci.2026.115903

**Published:** 2026-04-28

**Authors:** Myat Su Yin, Panhavath Meth, Peter Haddawy, Dominique J. Bicout, Theerapong Krajaejun

**Affiliations:** 1 Faculty of Tropical Medicine, Mahidol University, Ratchathewi, Thailand; 2 Faculty of ICT, Mahidol University, Nakhon Pathom, Thailand; 3 Bremen Spatial Cognition Center, University of Bremen, Bremen, Germany; 4 University Grenoble Alpes, CNRS, Grenoble INP, VetAgro Sup, TIMC, 38000 Grenoble, France; 5 Department of Pathology, Faculty of Medicine Ramathibodi Hospital, Mahidol University, Bangkok, Thailand

**Keywords:** Applied sciences, Environmental analysis, Environmental science

## Abstract

*Pythium insidiosum* is an aquatic oomycete causing pythiosis, a serious disease in humans and animals, particularly dogs and horses. Treatment is limited and often requires surgery, leading to substantial health and economic impacts. In Thailand, infections occur mainly in rural areas with frequent environmental exposure and limited healthcare. Despite its importance, the environmental drivers of the persistence of the organism remain poorly understood. This study assessed the current environmental suitability for *P. insidiosum* and examined changes through 2100 under different climate scenarios. A two-step modeling approach was used, incorporating confirmed occurrence records and key environmental variables. The highest suitability was found in the irrigated lowlands, particularly in the Chao Phraya Basin. Under higher-emission scenarios, suitable areas expanded into parts of northeastern and eastern Thailand, while the upland regions consistently showed low suitability. These findings highlight uneven future risk and support the need for coordinated One Health surveillance.

## Highlights

• Mapped current and future environmental suitability of *Pythium insidiosum* in Thailand
• Used a zero-inflated and MaxEnt modeling approach to map pathogen suitability
• Highest suitability occurred in irrigated lowlands, especially the Chao Phraya Basin
• High-emission scenarios increased suitability in parts of the northeast and east

## Graphical abstract

**Figure:**
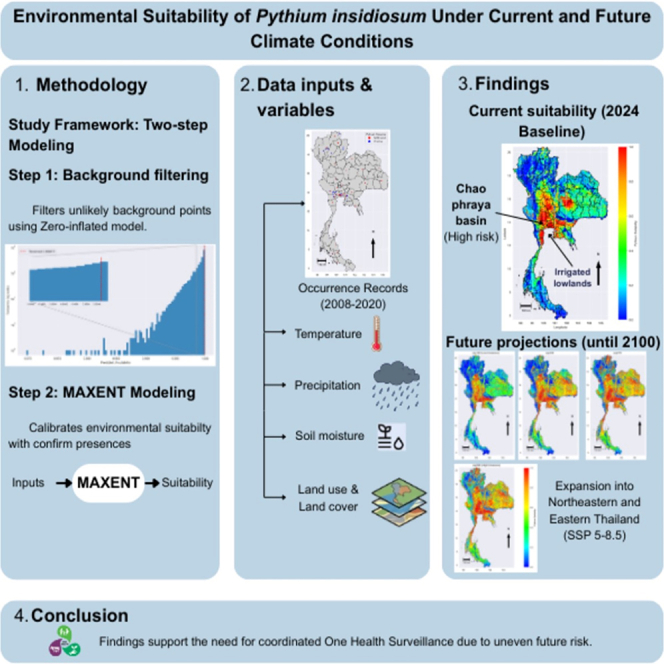

## Introduction

*Pythium insidiosum* is an aquatic fungus-like oomycete that causes pythiosis, a severe and often fatal disease in humans and animals. The organism produces motile zoospores that disperse in water and infect tissue through open wounds or skin exposure.^1^ Unlike true fungi, *P. insidiosum* lacks drug-target enzymes in the sterol biosynthesis pathway, making it intrinsically resistant to standard antifungal treatments.^2^ This resistance leaves few effective options, often necessitating surgical intervention and resulting in high morbidity and mortality. In endemic regions, most infections occur in tropical and subtropical lowlands where stagnant water creates suitable habitats, with the highest burden reported in Thailand and India.^3^^,^^4^^,^^5^^,^^6^^,^^7^ The pathogen has been detected in aquatic environments such as rice fields, reservoirs, and swampy lowlands,^6^ with Thailand recognized as the main endemic focus.

Since the 1980s, confirmed human and animal cases in Thailand have increased steadily.^6^^,^^7^ Vascular infection, the most lethal form of human pythiosis, is frequently associated with thalassemia or hemoglobinopathies and can lead to progressive arterial invasion, gangrene, aneurysm, and fatal hemorrhage.^8^^,^^9^ Ocular infection, the most common form, often affects individuals without underlying conditions and may require surgical removal of the eye to control disease progression.^6^ Beyond clinical burden, environmental surveys in Thailand have identified *P. insidiosum* in irrigation canals, flooded rice fields, and urban water bodies, indicating that rural and peri-urban landscapes can act as reservoirs.^3^^,^^10^^,^^11^ Detections are spatially and temporally sparse, a typical pattern of environmentally restricted pathogens influenced by climate and factors on the land surface. This sparseness complicates the estimation of habitat suitability when nondetections cannot be confirmed as true absences.

Environmental conditions are central to *P. insidiosum* ecology. The pathogen requires surface water for the dispersal of zoospores, can survive dry periods through the formation of oospores, and is influenced by temperature, vegetation, salinity, and land use. Despite global advances in modeling *P. insidiosum* distribution, no published study has estimated its environmental suitability in Thailand or projected how suitability might shift under climate change. Climate change can alter the distribution of infectious diseases by reshaping the temperature, precipitation, and hydrological conditions that support the persistence and transmission of pathogens.^12^^,^^13^ For *P. insidiosum*, projected increases in rainfall, flooding, and mean temperatures can expand suitable habitats into new areas, increasing the risk of exposure.

Among various species distribution modeling (SDM) algorithms, maximum entropy (MaxEnt) was selected for this study because of its strong predictive performance and robustness. Comparative evaluations of SDM methods have shown that MaxEnt consistently outperforms other algorithms, such as BIOCLIM and DOMAIN, particularly when using presence-only data.^14^^,^^15^ Furthermore, it offers a flexible framework for modeling complex non-linear relationships between species and environmental variables.^16^ Finally, MaxEnt has been shown to remain reliable when the occurrence data are spatially biased or the sample sizes are limited,^17^ which makes it a good candidate for modeling the distribution of *P. insidiosum* in the absence of systematic absence data. Consequently, MaxEnt has been extensively used in ecological and biodiversity research to model species distributions across a wide range of taxa and geographic regions.

The World Health Organization (WHO) operational framework for building climate-resilient health systems emphasizes the integration of climate information into surveillance, modeling, and preparedness, with complementary guidance on climate-informed early warning systems for infectious diseases.^18^^,^^19^ In this context, considering pythiosis within a One Health framework may indicate shared environmental risk factors for human and animal infections and the need for integrated monitoring for prevention. This study addresses three gaps: identifying environmental factors associated with *P. insidiosum* presence in Thailand, estimating current suitability at the national level, and projecting changes under future climate conditions. To achieve this, we applied a two-step modeling framework: First, a zero-inflated model was used to filter background points unlikely to support the pathogen; then, a MaxEnt model was calibrated on confirmed presences. The baseline period (2008–2020) was projected to 2024 to reflect near-current conditions and extended to the end of the century under four shared socioeconomic pathway (SSP) emission scenarios (SSP1-2.6, SSP2-4.5, SSP3-7.0, and SSP5-8.5). We then quantified how suitability trajectories vary between regions and provinces.

## Results

This section presents results of habitat suitability modeling for *P. insidiosum*, including present-day suitability, baseline and fine-tuned model performance, and projections to 2024 and future climate scenarios under multiple SSPs.

### Present-day projection (2024)

This section reports results in four subsections addressing initial model performance, outcomes after fine-tuning, and the relative importance of predictors.

#### Initial baseline model performance

**Figure 1:**
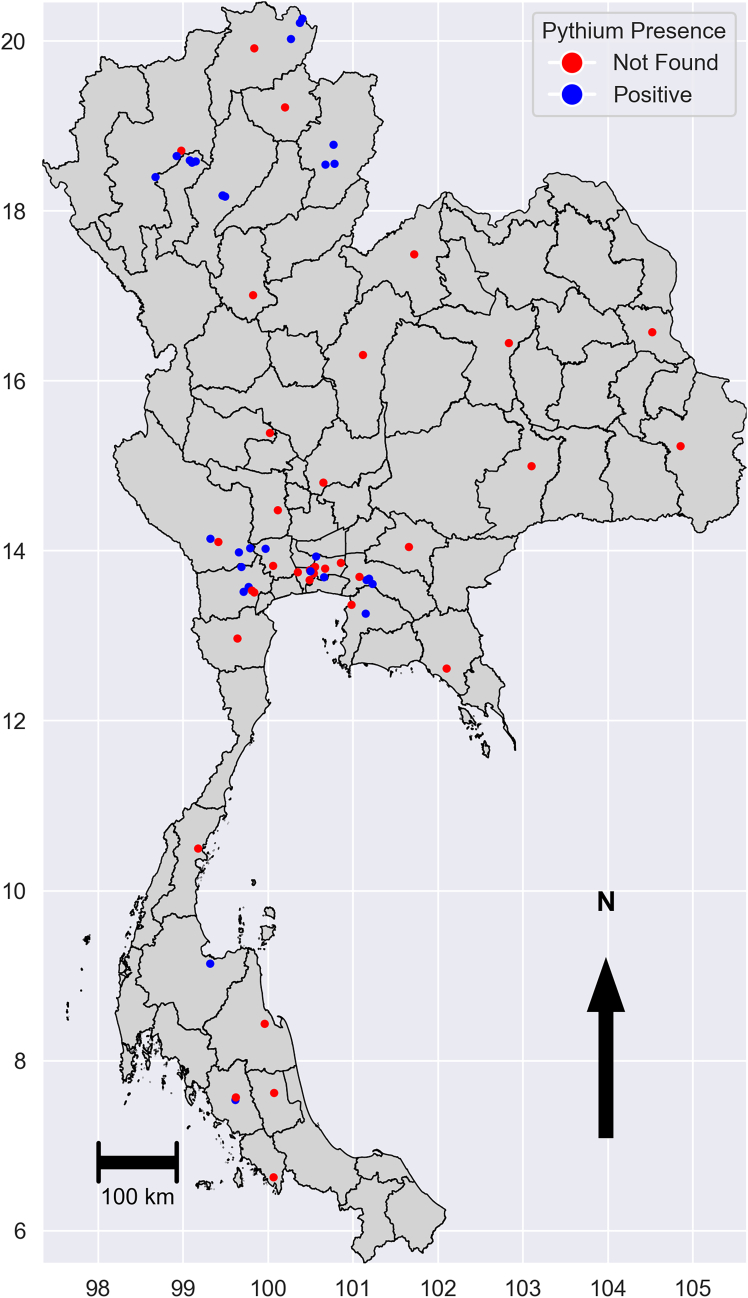
Map of sample collection areas: blue dots indicate positive sample sites, and red-colored dots represent sites without positive samples The map in this figure was produced using Python version 3.12. Source of shapefile: United Nations Office for the Coordination of Humanitarian Affairs https://data.humdata.org/dataset/thailand-administrative-boundaries.

**Figure 2:**
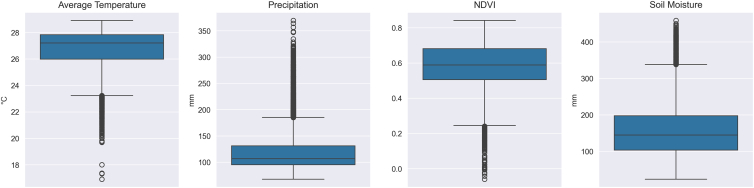
Boxplots showing the distribution of environmental variables at 4-km grid resolution across all provinces from 2008 to 2020, used in MaxEnt modeling Variables include average temperature (°C), precipitation (mm), normalized difference vegetation index (NDVI), and soil moisture (mm). In each boxplot, the central line represents the median, boxes indicate the interquartile range (25th–75th percentiles), whiskers extend to 1.5× the interquartile range, and points denote outliers.

**Figure 3:**
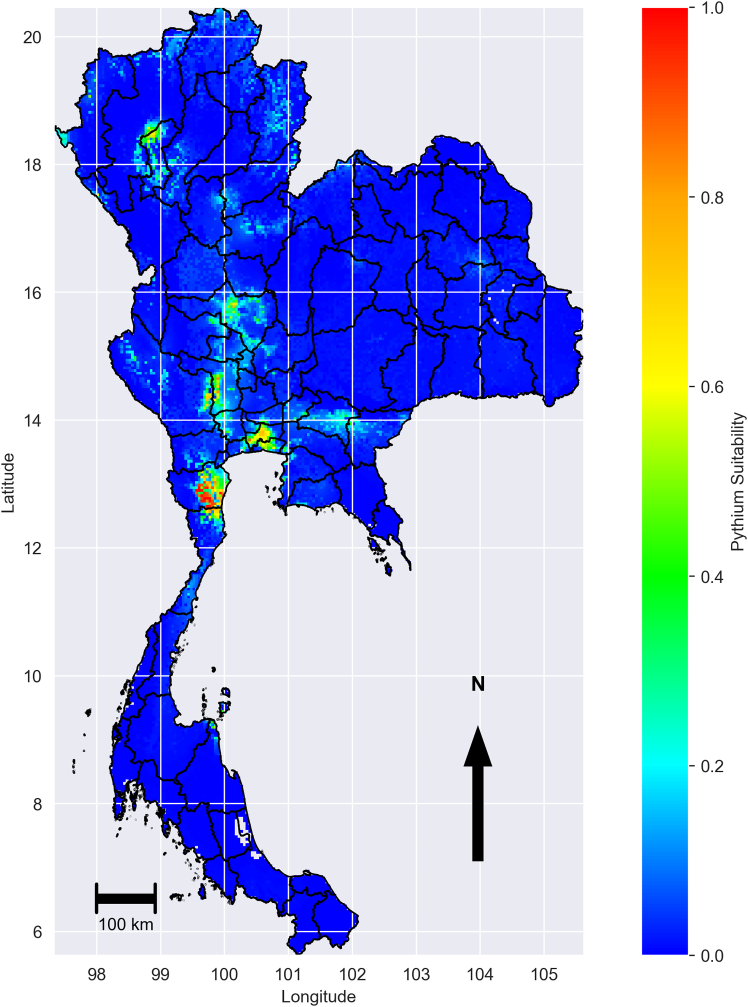
Predicted environmental suitability for *P. insidiosum*, generated using MaxEnt with default settings and unfiltered background points. Suitability values range from 0 (blue, lowest suitability to presence conditions) to 1 (red, highest suitability). The map in this figure was produced using Python version 3.12. Source of shapefile: United Nations Office for the Coordination of Humanitarian Affairs https://data.humdata.org/dataset/thailand-administrative-boundaries

We started by running MaxEnt with default settings, presence points (Figure 1), unfiltered background points and environmental variables (Figure 2) to establish an initial prediction surface (Figure 3). This model predicted broad areas of high suitability beyond the known range of *P. insidiosum*, including northern, northeast, and southern provinces that are environmentally unsuitable. These overpredictions resulted from background points drawn from environments not representative of the presence sites, which reduced the model’s ability to discriminate suitable from unsuitable conditions.

**Figure 4:**
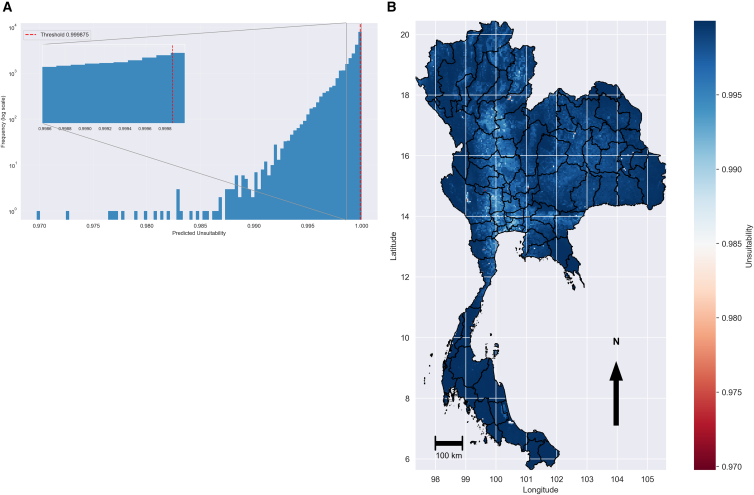
ZIP filtering of background points (A) Histogram of predicted unsuitability (*π*) values for *Pythium* on a log frequency scale. Predictions fall within 0.970–1.000, with a red dashed line marking the exclusion threshold (*π* = 0.999875) observed among presence sites; the inset highlights variation near the threshold. (B) Spatial distribution of *π* values across Thailand, with a color scale from 0.970 to 1.000, showing high predicted unsuitability in the southern peninsula, northeastern highlands, and northern uplands. The map was produced in Python 3.12 using shapefiles from the United Nations Office for the Coordination of Humanitarian Affairs (https://data.humdata.org/dataset/thailand-administrative-boundaries). (A) Predicted environmental suitability with ZIP-filtered background (B) Classification of predicted environmental suitability.

To address this, we applied a zero-inflated Poisson (ZIP) filtering approach before final model training. The histogram of predicted unsuitability values (*π*) for all background points (Figure 4A) was strongly right-skewed, with most sites showing values close to 1.0. The exclusion threshold was set to the maximum predicted unsuitability observed among confirmed presence sites (*π* = 0.999875) and is shown as a red dashed line near the upper tail of the distribution.

The spatial map of the *π* values (Figure 4B) shows darker blue shades indicating higher predicted unsuitability, approaching 1.0, and warmer red shades indicating lower values. The highest unsuitability was concentrated in the southernmost coastal provinces, the elevated terrain of the northeast, and the upland forest areas of the north. These areas generally experience cooler temperatures, more extensive forest cover, or limited standing water compared to locations where *P. insidiosum* has been detected.

**Figure 5:**
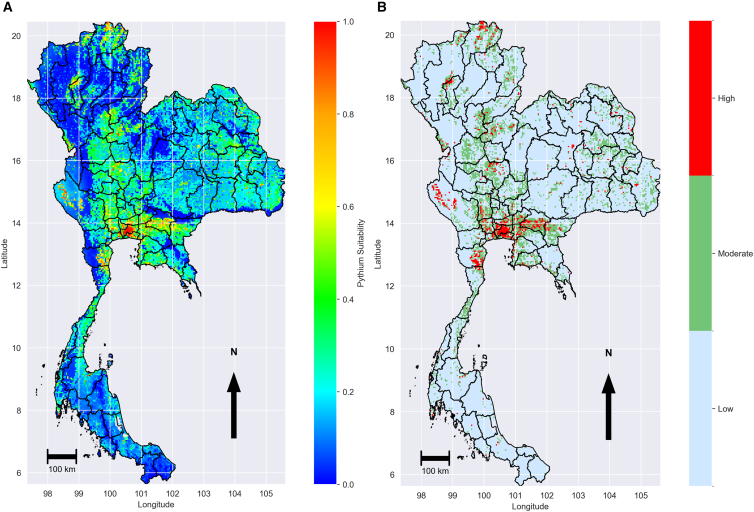
Predicted environmental suitability for *P. insidiosum* using MaxEnt with ZIP-filtered background (A) Suitability map with continuous values between 0 (blue, lowest suitability to presence conditions) to 1 (red, highest suitability) that concentrated in central floodplains and irrigated valleys, with reductions in uplands and dry zones. (B) Classification of predicted environmental suitability into three categories: high (≥0.60), moderate (0.30–0.59), and low (<0.30). High-suitability areas are concentrated in small clusters within low-lying irrigated landscapes, moderate suitability occurs in scattered patches often linked to seasonal water bodies, and low suitability dominates uplands, dry interiors, and much of the southern peninsula. The maps in this figure were produced using Python version 3.12. Source of shapefile: United Nations Office for the Coordination of Humanitarian Affairs https://data.humdata.org/dataset/thailand-administrative-boundaries. (A) Suitability map for 2024 (B) Differences between 2024 and baseline.

Applying this filter removed approximately 12.5% background points, excluding about 3,000 environments less favorable than any known occurrence. The ZIP-filtered MaxEnt model (Figure 5) produced a suitability surface on a continuous scale 0–1 (blue to red), with high values (≥0.60) concentrated in the central floodplains and smaller irrigated valleys in the northern province of Chiang Mai, while substantially reducing predictions in ecologically implausible areas of the South and much of the Northeast. Compared to the unfiltered model (Figure 3), the filtered version had a much lower Akaike information criterion (AIC) (143.5 vs. 203.9) and a higher test area under the curve (AUC) (0.922 vs. 0.808), indicating both a better fit and improved discrimination between presence and background points.

MaxEnt regularization was tuned using the ZIP-filtered background, testing multipliers from 0.1 to 5.0. The lowest mean corrected AICc occurred at 0.5, but the difference from 1.0 was minimal (see Figure S8). Models with a multiplier of 0.5 produced steeper response curves, reflecting greater complexity and potential overfitting, whereas 1.0 yielded smoother curves and more stable predictions across cross-validation replicates. The AUC of training (training AUC) and training gain both declined with increasing regularization (see Figures S9A and S9B). Cross-validation showed that the final model retained good predictive performance, with a mean training AUC of 0.88 (±0.01) and a mean test AUC of 0.78 (±0.16) (Figure S2-2B). To complement this threshold-independent measure, we also evaluated omission rates, which quantify the proportion of known presences predicted as absent. At the minimum training presence threshold, both training and test omission rates were zero across all folds. While AUC provides a widely used measure of discrimination, it does not assess probability calibration; omission rates therefore offer an important complementary measure of model accuracy. On this basis, a regularization multiplier of 1.0 was selected to balance model fit, predictive accuracy, and generalizability.

#### Fine-tuned baseline model results

The final prediction, generated at 4 × 4 km resolution (Figure 5A), was classified into three suitability categories using natural breaks^20^: high (≥0.60), moderate (0.30–0.59), and low (<0.30) (Figure 5B). Areas of high suitability (red) occurred in small, localized clusters rather than broad zones, primarily in the lower Chao Phraya Basin (central region; e.g., Ayutthaya, Pathum Thani, and Suphan Buri) and in certain irrigated zones near the Gulf of Thailand (e.g., Samut Sakhon and Samut Songkhram). Moderate suitability (green) appeared in scattered patches adjacent to clusters of high suitability and in parts of the north and west (e.g., Chiang Mai and Tak), where seasonal water bodies and floodplain agriculture are present. Low suitability (blue) dominated most of the country, including the southern coastal provinces (e.g., Surat Thani and Nakhon Si Thammarat), northeastern highlands (e.g., Khon Kaen and Udon Thani), and northern upland interiors (e.g., Chiang Rai), where topography, climate, or land cover differ from known presence environments. In general, the spatial pattern indicates that suitable habitats for *P. insidiosum* are highly fragmented and closely associated with low-lying areas that retain water.

#### Predictor importance results

We evaluated the role of each environmental predictor using permutation importance, percent contribution, and jackknife analysis (see Figure S10). Precipitation was ranked highest by permutation importance (41.3%), followed by land use (23.9%) and soil type (18.8%). Percent contribution placed land use first (35%) and precipitation second (24.7%). Jackknife analysis showed that precipitation had the highest standalone gain, while removing precipitation or land cover caused the most significant loss in gain. NDVI, although less important in permutation (6.5%), had a higher standalone gain than soil, indicating shared information with other variables. The temperature showed low importance for the permutation (2.8%) but contributed a unique explanatory power to the jackknife results.

The response curves (see Figure S11) indicated that suitability declined when precipitation exceeded 100 mm/month and increased markedly at temperatures above 27°C, suggesting a potential thermal threshold for *P. insidiosum*. The suitability was higher in the classes of irrigated and cultivated land cover (disturbed by humans), whereas forests and sparsely vegetated barren land were associated with lower suitability. Suitability also tended to decrease with increasing vegetation greenness (NDVI), with higher suitability observed in open, cultivated landscapes than in areas with dense canopy cover. To support reproducibility, the complete MaxEnt model coefficients are provided in the Supplementary Materials as.lambdas files (see Figures S28, S29, S30, S31, S32, S33, S34, S35, S36, and S37), which contain all parameters used in the analyses.

### Future suitability projection results

The results so far are based on the fitted model, which incorporates occurrence records from previous studies and environmental data from 2008 to 2020, serving as a historical reference that we refer to as the baseline. Present-day suitability, though, may differ due to recent climate variability and ongoing trends. Compared to baseline, the 2024 climate layers showed modest but spatially uneven changes, as annual precipitation decreased by about 20–120 mm in much of the northeast and several southern provinces, while mean temperature increased by 0.2°C–1.2°C, with the most substantial increases in the south and east. NDVI remained unchanged throughout the country, but soil moisture decreased by 10–75 index units in some coastal and upland areas (Table 1).

**Table 1:** Mean changes in environmental predictors between the 2008–2020 baseline and the 2024 projection

Variable	Change from 2008 to 2020 to 2024
NDVI	±0.0–0.1 units, minimal change nationwide
Precipitation (PPT)	−20 to −120 mm/year, with strong decreases in the northeast and south
Mean temperature (Tavg)	+0.2 to +1.2 °C, with the largest increases in the south and east
Soil moisture (SM)	−10 to −75 units, with decreases in coastal and upland areas

Precipitation and soil moisture declined mainly in the northeast, south, and coastal areas; temperature increases were most pronounced in the south and east.

#### Projection to present-day (2024)

**Figure 6:**
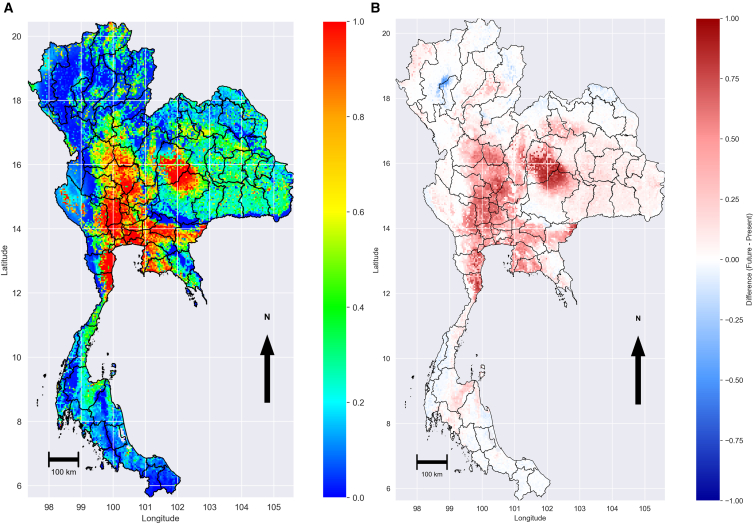
Projection of *P. insidiosum* suitability for 2024 and changes relative to the 2008–2020 baseline (A) Suitability map for 2024, projected using the baseline MaxEnt model with updated 2024 temperature and precipitation layers; colors range from blue (low suitability) to red (high suitability). (B) Difference map showing changes in suitability between 2024 and the 2008–2020 baseline, with red areas indicating increases and blue areas indicating decreases. The maps in this figure were produced using Python version 3.12. Source of shapefile: United Nations Office for the Coordination of Humanitarian Affairs https://data.humdata.org/dataset/thailand-administrative-boundaries.

To assess the impact of recent climatic changes on *P. insidiosum* suitability, the baseline model was projected to 2024 using updated temperature and precipitation layers, while NDVI, land cover, and soil moisture were held constant. The projection to 2024 (Figure 6A, hereafter referred to as the 2024-projection) and the corresponding difference between the baseline and this projection map (Figure 6B) provide a direct comparison of recent suitability patterns with the historical baseline. The difference map shows that highly suitable areas were contracted in the upper north and northeast. At the same time, smaller patches of expansion emerged in the lower central plain and parts of western Thailand, where warmer conditions coincided with adequate moisture. High-suitability areas continued to occur in the central floodplains (Ayutthaya, Pathum Thani, Suphan Buri, and adjacent provinces) and the lower Chao Phraya basin (Lop Buri, Nakhon Sawan, and Chai Nat), with slight expansion toward the western borders (Kanchanaburi and Ratchaburi) and reductions along parts of the northern (Phichit, Phitsanulok) and eastern borders (Nakhon Ratchasima, Buriram). Central floodplain provinces generally maintain warm conditions of 28–32°C, while northern and eastern provinces with upland areas are several degrees cooler (see Figure S4). This temperature pattern is consistent with laboratory findings *P. insidiosum*, which thrives at 28°C–37°C, can persist up to 42 °C, and is inhibited at lower temperatures (e.g., 8°C), which explains the higher suitability of warm lowlands compared to cooler highlands.^21^

#### Projections to the future

Building on the 2024-projection, we extended the analysis to the end of the 21st century using the climate output of the ACCESS-CM2 model in CMIP6 under four SSPs (SSP1-2.6, SSP2-4.5, SSP3-7.0, and SSP5-8.5), representing low to very high emissions. The climate variables (temperature and precipitation) were bias corrected, down-scaled from 100-250 km–4 km resolution, and aligned with the baseline suitability model. Besides temperature and precipitation, NDVI, land cover, land use, and soil moisture were kept constant, since high-resolution projections at comparable scales are not available. Studies suggest that Thailand’s land use patterns will remain relatively stable in moderate scenarios until 2100, with urban growth affecting only a small fraction of the total land area.^22^^,^^23^^,^^24^ We projected temperature and precipitation for four time periods (2021–2040, 2041–2060, 2061–2080, and 2081–2100) to inform both near- and long-term planning. Because these projections extend beyond the conditions used to train MaxEnt models, we applied a multivariate environmental similarity surface analysis (MESS).^25^ MaxEnt estimates species-climate relationships from the range of conditions represented in the training data; projections beyond this range involve extrapolation into novel environments where predictions are less certain.^25^^,^^26^ To evaluate the extent of extrapolation, MESS assigns each grid cell a score based on whether the projected climate variables fall within or outside the training range. Positive scores indicate conditions within the range, while values of zero or less identify novel climates, meaning combinations of variables not present in the baseline data. For example, a cell may remain within the temperature range but exceed the training limits for rainfall; in such cases, MESS flags the grid cell as novel. The final score is determined by the most dissimilar variable, which means that any deviation from the training range is detected.

We classified cells into two categories: within-range (MESS <0) and out-of-range (MESS ≤0), corresponding to more and less reliable projections, respectively. Out-of-range areas indicate novel environmental conditions, where predictions are based on extrapolation. Provinces in which more than 50% of the area had MESS ≤0 were considered affected by extrapolation and are indicated with hatching on the suitability maps. The complete set of suitability projections with this overlay is provided in Tables S5, S6, S7, S8, and S9.

Cooler conditions remain in the northern highlands and along the Tenasserim Range (a mountain chain that extends through Myanmar, Thailand, and Malaysia), while the strongest warming areas are found in the central plains (Ayutthaya, Pathum Thani, Suphan Buri) and northeastern provinces (Ubon Ratchathani, Khon Kaen, Nakhon Ratchasima). Under higher-emission scenarios, warming also extends into the lower north and upper northeast.

Rainfall projections show greater variability than temperature, with differences between scenarios and regions (see Figures S2 and S3). Under SSP1–2.6, annual rainfall increases slightly from approximately 111 mm/month in 2018–2020 to more than 122 mm/month in 2081–2100, mainly after the middle of the century. SSP2–4.5 shows minor fluctuations, with drying in the east and northeast after 2040 but modest increases along the Andaman coast late in the century. SSP3-7.0 projects a more pronounced drying in 2041–2060 in northeast and central provinces (e.g., Chaiyaphum and Buri Ram), followed by partial recovery in southern coastal areas (e.g., Songkhla and Pattani). SSP5–8.5 indicates the strongest drying in the northeast and central interior by 2081–2100 (e.g., Yasothon and Amnat Charoen), while the southern coasts maintain or gain rainfall. In general, wetter conditions persist along the western highlands and southern coasts, while inland areas, especially the northeast, become drier under high-emission scenarios.

Climate projections show consistent warming, with more variable rainfall changes in Thailand. In the following section, we assess how these temperature and precipitation patterns translate into changes in *P. insidiosum* suitability.

#### Projected *P. insidiosum* suitability

**Figure 7:**
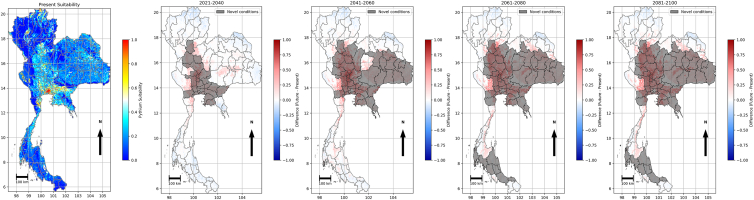
Projected environmental suitability for *P. insidiosum* under SSP1–2.6 relative to the 2008–2020 baseline The maps illustrate differences from the baseline, with warm colors indicating increases, cool colors presenting decreases, and hatching marking provinces where novel conditions cover more than half of the area. The maps were produced in Python 3.12 using shapefiles from the United Nations Office for the Coordination of Humanitarian Affairs (https://data.humdata.org/dataset/thailand-administrative-boundaries).

As described earlier, in the northeast and central regions, higher temperatures combined with reduced rainfall are expected to shorten the persistence of aquatic habitats for *P. insidiosum*. In contrast, stable or increasing rainfall in the west and south could sustain transmission risks despite warming. These climatic changes were incorporated into the suitability model by applying the projected temperature and precipitation from each SSP to the baseline model. To evaluate changes, we computed the differences between the baseline suitability and each future projection. Positive differences are described as gains (increased suitability), and negative differences as losses (reduced suitability). In low-emission scenarios (SSP1-2.6 and SSP2-4.5), changes were modest and gradual. Under SSP1-2.6 (Figure 7), high baseline suitability persisted in the central plain, with smaller gains in the lower central plain and western Thailand, and losses confined to areas of the northern highlands such as Chiang Rai and Mae Hong Son. Moderate suitability remained in the northeast provinces, while the northern highlands and much of the south stayed in lower classes. The hatching regions indicated new climates, adding uncertainty, but overall suitability increased in the agricultural core area of Thailand with only limited reductions in the north. SSP2–4.5 (see Figure S18) produced greater gains in the lower north and central plains, while losses in the northeast increased by approximately 5% relative to baseline during most future periods. The high-emission scenarios (SSP3–7.0 and SSP5–8.5) produced more substantial gains and earlier losses. SSP3–7.0 (see Figure S19) showed early gains in irrigated lowlands and losses in drier uplands, which intensified by the middle of the century. SSP5-8.5 (see Figure S20) produced the most significant and earliest changes, with gains exceeding +0.3 suitability units in the central plains, the lower north and west, and extensive declines in the northeast and the upper north from 2081 to 2100.

In all scenarios, spatial patterns were consistent: low-lying irrigated floodplains gained, while drier uplands lost, consistent with the ecology of *P. insidiosum*. The strength and timing of these changes scaled with the emission level. The low emission scenarios (SSP1-2.6 and SSP2-4.5) showed modest gains and losses that developed gradually. In contrast, high-emission scenarios (SSP3-7.0 and SSP5-8.5) produced earlier losses in the northeast and more substantial gains in the central plains, with northeast losses emerging early and central plain gains expanding thereafter.

Although soil moisture did not vary in future projections, its strong role in the baseline models provides a likely explanation for these results. Provinces that stayed stable or became wetter tended to gain, while those that dried lost on nearly every pathway. Because NDVI and land cover were kept constant in the model, future vegetation changes could further alter suitability, especially in rainfed agricultural areas such as parts of Nan and Phrae in the north and Khon Kaen and Buriram in the northeast. Permanent agricultural land in the lower Chao Phraya and Mae Klong basins showed the most significant gains, reflecting the combined influence of irrigation infrastructure and warmer conditions.

**Figure 8:**
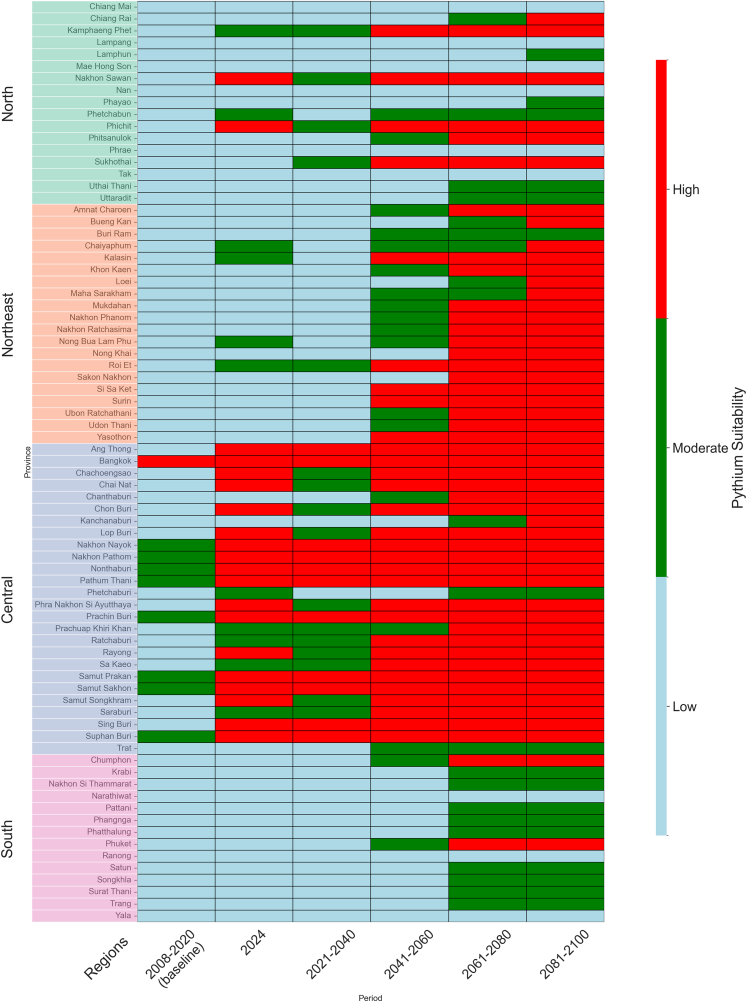
Projected suitability for *P. insidiosum* under SSP5–8.5, classified into high (≥0.60), moderate (0.30–0.59), and low (<0.30) categories at baseline (2008–2020), near-current (2024), and long-term projections Trends are not strictly monotonic due to non-linear climate drivers and threshold effects; central provinces show steady increases, while some northern and northeastern provinces show temporary gains followed by subsequent declines.

Since soil moisture indicates changes in suitability at the provincial level, we use provincial-level heat maps to visualize these changes from baseline through 2100. To illustrate spatial and temporal dynamics, results are shown in two formats for each SSP. The categorical figures (see Figures S21, S22, and S23) and Figure 8 classify the suitability into high, moderate, and low groups, using the thresholds described above (≥0.60, 0.30–0.59, <0.30). The colors distinguish the three categories. The continuous Figures S24, S25, S26, and S27 show the suitability probabilities on a scale of 0–1, shaded from blue (low probability) to red (high probability). The categorical view emphasizes shifts across thresholds, while the continuous view retains gradients and finer changes. In both sets, provinces are grouped by region on the y axis and periods are arranged on the x axis, highlighting province-by-province trajectories across t. The regions follow the conventional Thai division into *North*, *Northeast*, *Central*, and *South*. In the following discussion, results are primarily described using categorical maps, while probability maps are mentioned only when they reveal patterns not apparent from the categories.

Across the four SSPs, provincial trajectories exhibit distinct regional patterns. The summaries of the category shifts based on thresholds are provided below, with the provinces grouped into three suitability classes: high (≥0.60), moderate (0.30–0.59), and low (<0.30).

#### SSP-based projections (SSP1–2.6, SSP2–4.5, SSP3–7.0, SSP5–8.5)

Under SSP1–2.6 (see Figure S21), the changes in suitability are mild and gradual. In the north (Chiang Mai, Nan, Phrae, located in the northern highlands), provinces remain in the low category in all periods. In the northeast (Khon Kaen, Nakhon Ratchasima, Ubon Ratchathani), suitability is stable through baseline and 2024, with slight declines after 2041–2060. In the central region (Ayutthaya, Suphan Buri, Pathum Thani), suitability is stable through baseline and 2024, with modest declines later in the century. The lower Chao Phraya and Mae Klong basins (Samut Sakhon, Nakhon Pathom, Ratchaburi) remain in the high category. In the west (Kanchanaburi and Tak, within the Tenasserim Range), suitability stays in the low category, with probability maps confirming little change over time.

Under SSP2–4.5 (see Figure S22), the changes in suitability are moderate. In the north (Chiang Mai, Chiang Rai, Lamphun), provinces remain in the low category, although probability maps show brief midcentury increases in Chiang Mai and Lamphun before returning to low levels. In the northeast (Nakhon Ratchasima, Roi Et), suitability shifts from moderate through the initial period and 2024 to low by 2041–2060 and after. In the central region (Suphan Buri, Ang Thong, Chai Nat), the suitability also drops from moderate to low, while Bangkok, Pathum Thani, and Nonthaburi remain in the high category. In the south (Surat Thani, Nakhon Si Thammarat), suitability remains in the low category, with probability maps showing only slight improvements at the end of the century.

Under SSP3-7.0 (see Figure S23), the changes in suitability are substantial. In the north (Chiang Mai, Chiang Rai, Lamphun), provinces remain in the low category throughout. In the northeast (Nakhon Ratchasima, Udon Thani, Khon Kaen), suitability decreases stepwise from moderate to baseline and 2024 to low by 2041–2060. In the central region (Bangkok, Pathum Thani, Nonthaburi), the provinces remain in the high category, while others (Ang Thong, Suphan Buri, Chai Nat) decline from moderate to low by the midcentury. In the south (Nakhon Si Thammarat, Surat Thani, Songkhla), provinces remain in the low category, although probabilities in Surat Thani show small late-century increases that never reach the moderate threshold.

Under SSP5–8.5 (Figure 8), suitability trajectories were not strictly monotonic across regions. Although this scenario involves a continuous rise in temperature, suitability is shaped by multiple interacting variables, including precipitation and soil moisture, which do not change uniformly over time. The combined influence of these drivers can produce temporary increases or decreases in suitability before further declines. Classification into discrete categories can also exaggerate apparent shifts near thresholds. In general, changes were more severe and uneven than in other scenarios. In the north (Chiang Mai, Lampang, Phayao, Phichit, Sukhothai, and Uttaradit), the provinces transition from a low category at baseline to a moderate category by 2041–2060 and remain in this category thereafter, while most other northern provinces remain in the low category. In the northeast (Khon Kaen, Maha Sarakham, Nakhon Phanom, Nakhon Ratchasima, Roi Et), provinces remain in the moderate category through 2041–2060 before declining to low later in the century, while other areas of the region change earlier from moderate to low. In the central region, a large cluster of provinces (Bangkok, Ang Thong, Chachoengsao, Chon Buri, Nakhon Nayok, Nakhon Pathom, Nonthaburi, Pathum Thani, Phra Nakhon Si Ayutthaya, Samut Prakan, Samut Sakhon, Samut Songkhram, Saraburi, Suphan Buri) changes from moderate at baseline to high in 2041–2060 and stays high thereafter. Chanthaburi, Kanchanaburi, Lop Buri, Ratchaburi, and Rayong remain moderate, while Chai Nat, Prachuap Khiri Khan, and Sing Buri drop to a low in 2061–2080. In the south (Krabi, Nakhon Si Thammarat, Ranong), the provinces change from low at baseline to moderate by 2041–2060, while most other southern provinces remain low. The probability map (see Figure S27) also confirms these patterns, indicating intensification in the central region and gradual increases in selected southern provinces.

## Discussion

This study presents the national-scale modeling effort to identify environmental factors associated with *P. insidiosum* in Thailand that incorporate current and future climate projections. We used confirmed presence data and environmental predictors to estimate present suitability, extend projections to 2024 relative to the 2008–2020 baseline, and then project future changes under different climate scenarios. To reduce bias in presence-only modeling, we filtered background points using probabilities from a ZIP model, removing areas structurally unlikely to support *P. insidiosum* and improving environmental contrast in the MaxEnt model. This combined approach reduced overprediction in ecologically implausible zones and achieved strong discriminatory performance across cross-validation runs. The variable importance analysis highlighted precipitation and land use as the dominant predictors, followed by soil properties, vegetation greenness (NDVI), land cover, and temperature. These results align with field observations linking the pathogen to irrigated agriculture, floodplains, and persistent surface water. Because awareness of pythiosis among clinicians and veterinarians is limited in many parts of Thailand, areas of high predicted suitability but no recorded cases may partly reflect under-recognition rather than the true absence of the pathogen.

Suitability was concentrated in lowland regions with water bodies that persist year-round during the baseline period (2008–2020), particularly central floodplains, the lower Chao Phraya Basin, and irrigated valleys in the northeast. Provinces such as Ayutthaya, Pathum Thani, and Suphan Buri, which combine irrigated landscapes with high soil moisture, consistently appeared as long-term hotspots. The spatial distribution is consistent with the ecology of *P. insidiosum*, which favors aquatic or semi-aquatic habitats such as rice fields, canals, and aquaculture systems. Environmental sampling and pathogen isolation from rice fields and reservoirs in Thailand support these associations.^3^^,^^10^ Some areas with favorable environmental conditions but no official reports, particularly in eastern Thailand, may reflect under-surveillance or limited case detection, given the difficulty of confirmation without a definitive laboratory investigation.^7^^,^^27^ These spatial patterns in Thailand, as a representative country in the Eastern Hemisphere, are broadly consistent with previous modeling efforts in Western regions. For example, in Rio Grande do Sul, Brazil, high suitability was also associated with lowland, water-retentive agricultural landscapes, particularly rice-growing areas.^4^ Similarly, in the Chincoteague National Wildlife Refuge, Virginia, the highest suitability occurred in wetland and marsh habitats with persistent water availability.^5^ Unlike these earlier studies, our projections under multiple SSPs show how emission-driven climatic changes could amplify gains in some irrigated lowlands while reducing persistence in regions prone to drying, suggesting both a shared ecological niche across continents and region-specific vulnerabilities in the future.

Future projections showed that high-emission scenarios (SSP5–8.5) produced early and extensive losses in areas of pathogen suitability in the northeast, with gains emerging in irrigated lowlands by the mid-century. Low-emission scenarios (SSP1–2.6) maintained near-baseline conditions for decades, with smaller, localized gains in the lower central plain and western Thailand. The highlands remained unsuitable, reflecting cooler temperatures and the limited persistence of surface water. This pattern is consistent with laboratory evidence that *P. insidiosum* grows optimally at 28 to 37° C, survives up to 42 °C, and is inhibited at 8° C,^6^^,^^21^ making high-altitude regions persistently unfavorable. Increases in mean temperature, particularly in the south and east, can prolong the viability of zoospores and the growth of mycelium in aquatic habitats where moisture is sustained. In contrast, projected drying in parts of the northeast, especially nonirrigated uplands, can limit the persistence of the pathogen regardless of warming. In all scenarios, provinces where soil moisture remained stable or increased tended to gain suitability, while those that dried experienced losses in nearly every pathway. Because NDVI and land cover were kept constant in the model, future vegetation changes could further alter suitability, especially in rainfed agricultural areas such as parts of Nan and Phrae in the north and Khon Kaen and Buriram in the northeast. Permanent agricultural areas in the lower Chao Phraya and Mae Klong basins showed the largest gains, due to irrigation infrastructure and warmer conditions.

Present-day hotspots remain concentrated in lowland areas with persistent surface water, particularly the central plains and irrigated areas of the northeast. Under higher-emission scenarios, gains are projected in the central plains, lower north, and parts of the east, with losses in the northeast, uplands, and drier interior provinces. The expansion of suitability in the eastern and northeastern lowlands under high-emission scenarios is notable given the high prevalence of thalassemia and hemoglobinopathies in these regions, which are associated with severe vascular pythiosis and high mortality.^8^^,^^28^ Awareness among clinicians is also limited, as shown by survey data from a major Thai teaching hospital, highlighting the need for targeted training to improve early recognition and diagnosis.^29^ In general, most human cases occur in Thailand and India, whereas animal cases are concentrated in the Americas, reflecting both ecological conditions and detection biases.^6^ Clinical and environmental surveillance is important for detecting potential outbreaks of *P. insidiosum*, but it may not fully capture the actual situation due to under-reporting. Environmental changes could also affect persistence and spread. In low-lying areas, the risk of flooding may increase due to land subsidence and rising sea level, whereas prolonged dry periods may limit persistence elsewhere. Additional factors, such as salinity and flood depth, although not modeled here, could also influence coastal provinces and should be considered in future analyses of *P. insidiosum* distribution and disease burden.

The MaxEnt framework can be applied to other water-associated pathogens with limited surveillance data and specific ecological requirements. Integrating climate, land use, and probabilistic background filtering provides a reliable estimation of environmental suitability without confirmed absences. A One-Health approach that links clinical and environmental surveillance would improve early detection, especially in agricultural landscapes where human and animal exposure overlap. The approach is also applicable to neighboring countries with similar agroecological conditions, such as Cambodia, Laos, and Myanmar, where targeted surveillance may detect early changes in pathogen distribution driven by climate change.

This study provides the national-scale projections of *P. insidiosum* suitability in Thailand under multiple climate scenarios. Present-day hotspots were concentrated in lowland areas with persistent surface water, particularly central plains and irrigated areas of the northeast. Under higher-emission scenarios, suitability gains were projected in the central plains, lower north, and parts of the east, while longer-term losses became more pronounced in the northeast and some northern upland provinces.

These findings highlight the need for targeted surveillance and improved diagnostic readiness in areas where environmental conditions are favorable, but cases have not been reported. Future work should include validation with geographically precise case data from both human and animal infections, expanded environmental sampling in under-surveyed regions, and incorporation of additional hydrological and land-use projections. Using multiple climate models will strengthen confidence in long-term projections. Although focused on Thailand, the modeling framework can be adapted to other countries with similar ecological conditions and applied as part of regional One Health strategies to address climate-sensitive infectious diseases.

### Limitations of the study

This study has several limitations. The occurrence data were compiled from published environmental and clinical studies, which can introduce bias into the data toward known endemic areas. Although ZIP filtering reduced overprediction, data limitations could still influence spatial patterns. Data from human cases with precise geographic coordinates were not available, limiting clinical validation. In addition, environmental suitability does not necessarily correspond to disease prevalence. Infection risk is strongly influenced by host susceptibility and exposure patterns. Previous studies indicate that individuals with underlying conditions such as thalassemia or hemoglobinopathies are particularly vulnerable to pythiosis.^6^^,^^7^^,^^8^ While our study incorporated data from previous seroprevalence surveys, these were available only to a limited extent and did not comprehensively capture host-specific conditions. Consequently, even in regions with high environmental suitability, the absence of reported cases may reflect limited clinical detection (as pythiosis is underrecognized and diagnostic tests are often unavailable) or the lack of susceptible hosts present in these areas, rather than a true absence of infection risk. Future research should integrate broader seroprevalence data, host susceptibility, and behavioral exposure with ecological models to improve disease prediction and guide targeted surveillance. Differences in spatial resolution between datasets may reduce fine-scale accuracy, and seasonal variation was not explicitly modeled. NDVI, land use, and land cover were treated as static, so future agricultural or urban changes were not reflected. Reliance on a single general circulation model (ACCESS-CM2) means that results represent one plausible trajectory; Future work should incorporate multi-model ensembles, drawing on outputs from CMIP6 global climate models such as HadGEM, MPI-ESM, and GFDL, to better capture uncertainty. The model was calibrated for Thailand, and further validation will be needed before application to other countries.

## Resource availability

### Lead contact

Further information and requests for resources should be directed to and will be fulfilled by the Lead Contact, Dominique Bicout (bicout@ill.fr).

### Materials availability

This study did not generate new unique materials or reagents.

### Data and code availability

• All data used in this study are publicly available. The datasets analyzed include Pythium insidiosum occurrence records from previously published studies (Lohnoo et al., 2019; Mar Htun et al., 2021; Supabandhu et al., 2008), as listed in the key resources table.
• This study did not generate custom code. SDM was performed using the graphical user interface of MaxEnt (v3.4.4), and analyses were conducted using publicly available software, as listed in the key resources table.
• This study did not generate new unique reagents or materials.

## Acknowledgments

The authors acknowledge the support of the 10.13039/501100004704National Research Council of Thailand and 10.13039/501100004156Mahidol University to T.K.

## Author contributions

Conceptualization, T.K., M.S.Y., and D.J.B.; methodology, M.S.Y., P.M., T.K., and D.J.B.; investigation, M.S.Y., P.M., P.H., D.J.B., and T.K.; writing – original draft, M.S.Y., P.M., T.K., and D.J.B.; writing – review and editing, M.S.Y., P.M., T.K., D.J.B., and P.H.; funding acquisition, T.K.; resources, M.S.Y., P.M., P.H., D.J.B., and T.K.; supervision, M.S.Y., T.K., D.J.B., and P.H.

## Declaration of interests

The authors declare no competing interests.

## STAR★Methods

### Key resources table

**Table:** 

REAGENT or RESOURCE	SOURCE	IDENTIFIER
**Deposited data**		

*Pythium insidiosum human serum occurrence records (Thailand, 2008–2019)*	Lohnoo et al.^30^	Published dataset (https://doi.org/10.1093/mmy/myy030)
*Pythium insidiosum horse serum occurrence records (Thailand)*	Mar Htun et al.^31^	Published dataset (https://doi.org/10.1016/j.mycmed.2020.101085)
*Pythium insidiosum environmental water isolates (Northern Thailand)*	Vanittanakom et al.^11^	Published dataset (https://doi.org/10.1016/j.ijmm.2013.11.016)
*Pythium insidiosum environmental water samples (Central and Southern Thailand)*	Mar Htun et al.^10^	Published dataset (https://doi.org/10.3390/jof7040242)

**Software and algorithms**		

MaxEnt software (GUI) v3.4.4	Phillips et al.^32^	https://biodiversityinformatics.amnh.org/open_source/maxent
Python v3.12	Python Core Team^33^	RRID:SCR_008394
NumPy	Harris et al.^34^	RRID:SCR_008633
Pandas	McKinney et al.^35^	RRID:SCR_018214
Scikit-learn	Pedregosa et al.^36^	RRID:SCR_002577
Statsmodels	Seabold et al.^37^	RRID:SCR_016074
GeoPandas	Kelsey et al.^38^	https://geopandas.org
Matplotlib	Hunter^39^	RRID:SCR_008624

All remaining categories are intentionally left blank, as no biological reagents, organisms, or experimental models were used.

### Experimental model and study participant details

This study did not involve experimental models, human participants, or biological samples. All analyses were conducted using previously published occurrence data obtained from the literature.

### Method details

#### Occurrence data

We compiled *P. insidiosum* occurrence records from five environmental and serological surveys conducted throughout Thailand between 2008 and 2019. The dataset includes human serum samples, animal serum samples, and environmental water samples collected from multiple regions. All records represent confirmed positive occurrences of *P. insidiosum*; negative samples were not included because the MaxEnt model was constructed using presence-only data. The occurrence dataset comprised 21 human serum records, 8 horse serum records, and 38 environmental water records, each representing at least one confirmed positive detection. Human serum data was obtained from a national survey by Lohnoo et al.,^30^ which covered 21 provinces in the four main geographical regions of Thailand: northern, northeast, central, and southern. Horse serum data came from a veterinary surveillance program reported by Mar Htun et al.,^31^ with 150 serum samples collected from clinically healthy horses (43 from the northern region, 86 from the central region, 14 from the eastern region and 7 from the southern region). Water samples from agricultural and irrigation areas in northern Thailand resulted in 66 isolates (Chiang Mai: 8; Chiang Rai: 13; Lumphun: 20; Lumpang: 12; Nan: 6), collected from field reservoirs, irrigation channels, rice irrigation systems, and household reservoirs.^3^ Additional water samples from central and southern provinces were reported by Mar Htun et al.,^10^ including 500 samples collected from 100 sites in seven provinces. Bangkok (240) and Nakhon Pathom (60) in the Central region, Chonburi (15) and Chachoengsao (40) in the Eastern region, Kanchanaburi (55) and Ratchaburi (65) in the Western region, and Trang (25) in the Southern region. All sampling sites were georeferenced and classified as positive if at least one sample tested positive for *P. insidiosum*. The spatial distribution of the surveyed locations is shown in Figure 1. All data were obtained from previously published sources. We did not collect any new data on human or animal subjects; all data were obtained from previously published studies. To support spatial reference throughout the article, a provincial map of Thailand with regions is included (see Figure S7). Table S1 lists the references and sample types for all the occurrence records.

#### Environmental predictors

To estimate the environmental suitability of pythiosis occurrence in Thailand, we assembled a set of continuous and categorical environmental predictors. Continuous predictors included mean precipitation (PPT), mean average temperature (average of yearly means) (Tavg), soil moisture (SM), and normalized difference vegetation index (NDVI), each calculated from data from 2008 to 2020, aggregated into a 4 km grid and represented as a single raster layer. Categorical predictors were land cover (LC) and land use (LU). LC data were retrieved from the Copernicus land cover dataset^40^ and included categories such as croplands, flooded vegetation, forests, urban areas, and open water (see Table S2). LU data was obtained from the Land Development Department of Thailand^41^ and classified into 11 categories, such as aquaculture, rice paddies, surface water infrastructure, and other agricultural and urban land types (See Table S4).

All environmental layers were resampled onto a 4 km grid to match the spatial resolution of the coarsest input dataset and ensure comparability between predictors across the national extent. The layers were re-projected into the WGS 84 geographic coordinate system to maintain consistency with the coordinate reference system used for the occurrence points and to facilitate integration with other global datasets. Collinearity among predictors was assessed using variance inflation factors (VIF), with all values below 2.5, indicating acceptable independence for inclusion (see Table S4). Figure 2 summarizes the distributions of four environmental predictors in Thailand from 2008 to 2020. The average temperature remains relatively stable across regions, indicating consistent thermal conditions, while NDVI shows moderate variation in vegetation productivity. Precipitation and soil moisture vary more widely and are unevenly distributed, indicating strong spatial contrasts in water availability. These patterns suggest that temperature and NDVI represent stable background conditions, while precipitation and soil moisture are more likely to influence habitat suitability. The spatial layers of environmental and land use/land cover used in the models are provided in Figures S1 and S2.

#### Ecological niche modeling

Species distribution models are based on both species presence data and the range of environmental conditions in the study area. Because true absence data are rarely collected, researchers often characterize the environment using background points. These points are a type of pseudoabsence data that represents the conditions accessible to the species^42^ and help to establish contrasts between the locations where the species is known to occur and the wider set of available environments.^26^^,^^42^ However, background points cannot always distinguish genuinely unsuitable habitats, such as dry highlands or dense urban areas without standing water, from locations that may be suitable but were not recorded due to sparse sampling or diagnostic limitations.^43^^,^^44^

In our study, many of the locations surveyed had zero detections. To address this issue, we applied a zero-inflated modeling approach, evaluating both zero-inflated Poisson (ZIP) and zero-inflated negative binomial (ZINB) models. The ZIP model was selected on the basis of its lower AIC, indicating a better fit.^45^^,^^46^ We used MaxEnt’s default sampling of 24,000 background points throughout Thailand to represent the available environmental space, ensuring adequate coverage while maintaining computational efficiency. Within the ZIP model, this background sample was used to jointly estimate the probability of structural unsuitability (*π*) and expected detections at potentially suitable sites (*λ*), thus separating structural absences from non-detections resulting from incomplete sampling.

The predictor variables were identical to those described in the Environmental Predictors and Data Preparation section. Continuous variables (PPT, Tavg, SM, NDVI) were used in their original units, and categorical variables (LC, LU) were treated as factors. The ZIP model was specified as:

$$\left\{\begin{array}{c} logit\left(\pi \right)∼constant\\ log\left(\lambda \right)∼\text{PPT}+\text{Tavg}+\text{SM}+\text{NDVI}+\text{LC}+\text{LU} \end{array}\right)$$

where PPT = mean precipitation, Tavg = mean average temperature, SM = soil moisture, and NDVI = normalized difference vegetation index. The adjusted expected detections were calculated as *λ* × (1 − *π*), where *λ* is the expected detection count for suitable sites and *π* is the probability of structural unsuitability. This adjustment accounts for both environmental suitability and the possibility of non-detection. Interaction terms were not considered to maintain the simplicity of the model.

After fitting the model, the background points were filtered by excluding those with predicted structural unsuitability (*π*) greater than the maximum *π* observed between the presence sites. This rule is analogous to the lowest presence threshold (LPT) commonly used in species distribution modeling,^47^ with the difference that the LPT is applied to predicted suitability scores, whereas here we apply it to predicted unsuitability probabilities. In both cases, the cutoff corresponds to the least suitable known presence, ensuring that all retained background sites are at least as permissive as the marginal conditions under which the species has been observed.

In the second step, the filtered dataset was used in MaxEnt^26^ to model the ecological niche and distribution of *P. insidiosum*. MaxEnt estimates the probability distribution of environmental conditions that is closest to uniform while constrained by the observed presence data, producing a continuous suitability surface ranging from 0 (lowest suitability to the presence conditions) to 1 (highest suitability).

To reduce overfitting and improve generalizability, we applied regularization during MaxEnt model fitting. Regularization constrains model complexity by penalizing overly specific relationships between predictors and suitability. This penalty smooths the response curves, minimizes the influence of predictors that contribute little to the explanatory power, and improves the predictive performance of the model in unsampled areas. Regularization multipliers of 0.1, 0.5, 1, 2, and 5 were tested using only linear features to produce simple, interpretable response curves and to emphasize broad, monotonic relationships rather than complex higher-order interactions. The selection of the model was based on the corrected Akaike Information Criterion (AICc), the area under the receiver operating characteristic curve (AUC), and the regularized training gain.

The final model evaluation used 10-fold cross-validation. The presence data was divided into ten subsets; each model was trained on nine and tested on one. Continuous output was retained rather than applying a binary threshold, as binary classification can misrepresent risk patterns in under-sampled areas.

All analyzes were performed in Python version 3.12 using the package statsmodels for zero-inflated models and also collinearity checks. MaxEnt modeling was performed in the standalone MaxEnt software (version 3.4.4) via the graphical user interface (GUI). Random seeds were set for all stochastic processes to ensure reproducibility.

### Quantification and statistical analysis

Statistical analyses and data preprocessing were performed in Python (v3.12). We assessed collinearity among predictors and fitted Zero-Inflated Poisson (ZIP) regression models using the statsmodels library. Species distribution modeling was conducted using the standalone MaxEnt software (v3.4.4). Random seeds were fixed for all stochastic procedures to ensure reproducibility. To mitigate sampling bias from ecologically unsuitable background points, we implemented a ZIP filtering protocol prior to MaxEnt training. A ZIP model was fitted to the full dataset to estimate the probability of unsuitability (pi) for all background locations. We established a strict exclusion threshold at pi = 0.999875, corresponding to the maximum unsuitability value observed at confirmed presence locations. Background points exceeding this threshold were discarded to minimize false negatives and improve the discrimination between suitable and unsuitable conditions. MaxEnt models were trained using the ZIP-filtered background. We optimized the regularization multiplier by testing values from 0.1 to 5.0, selecting a final multiplier of 1.0 to balance Akaike Information Criterion (AICc) and omission rates. This selection prioritized model generalizability over the higher complexity observed with lower multipliers. Model performance was evaluated using the Area Under the Curve (AUC), yielding a mean test AUC of 0.78 ± 0.16 across cross-validation replicates. To assess the reliability of model extrapolations to the future until 2100, we computed a Multivariate Environmental Similarity Surface (MESS). Areas with highly negative MESS values (<−10) were identified as novel environments where predictor variables fell outside the training range; these regions (Figure 4B) should be interpreted with caution. The final suitability surface was classified into high (≥0.60), moderate (0.30 − −0.59), and low (<0.30) suitability categories using natural breaks. Final models were based on *n* = 67 confirmed *P. insidiosum* occurrence records. The initial background sample of *n* = 24,900 points was reduced by approximately 12.5% (3,000 points) following the ZIP filtration process.
