# Supplementary material for: Mapping environmental suitability for Pythium insidiosum under current and future climate conditions
Source: iScience. 2026 Apr 28;29(6):115903. doi: 10.1016/j.isci.2026.115903 (PMC13196145; doi:10.1016/j.isci.2026.115903)
Supplement: Document S1. Figures S1–S37 and Tables S1–S9 and Data S1 [file mmc1.pdf]

iScience, Volume *29*

Supplemental information

Mapping environmental suitability for *Pythium insidiosum* under current and future climate conditions

Myat Su Yin, Panhavath Meth, Peter Haddawy, Dominique J. Bicout, and Theerapong Krajaejun

Data S1: Environmental data, model performance, and projected suitability of *Pythium insidiosum* in Thailand

Table S1: Geographical locations of *Pythium insidiosum* records in Thailand from published studies

Reference, DOI	Sample type	Location	
		Latitude	Longitude
Mar Htun, Zin, et al. (2021), `10.3390/jof7040242`	Environmental water	13.770966	100.516366
		13.854501	100.859078
		13.807507	100.555620
		13.929843	100.568340
		13.787263	100.674502
		13.731485	100.541550
		13.652241	100.491406
		13.770022	100.494723
		13.686055	100.662055
		13.744389	100.352753
		13.257211	101.151760
		13.668917	101.191042
		13.652780	101.161328
		13.606459	101.232437
		14.020776	99.973345
		14.138575	99.324756
		13.978799	99.659559
		14.027293	99.791957
		13.806338	99.689354
		13.571397	99.774272
		13.514250	99.715126
		13.507072	99.840394
		7.534068	99.618507
Lohnoo, Tassanee, et al. (2019), `10.1093/mmy/myy030`	Human serum	18.775632	100.773042
		18.706064	98.981716
		17.005557	99.826371
		16.301669	101.119280
		15.383500	100.024553
		17.486023	101.722300
		16.569572	104.523121
		16.441936	102.835992
		15.228686	104.856422
		14.993002	103.102919
		14.799508	100.653371
		14.042070	101.660087
		13.819921	100.062168
		13.756331	100.501765
		12.964922	99.642588
		12.611340	102.103855
		10.493050	99.180020
		9.138239	99.321748
		8.430398	99.963122
		7.616682	100.074023
		6.623816	100.067374

Continued on next page

Reference, DOI	Sample type	Location	
		Latitude	Longitude
Vanittanakom, et al. (2014), 10.1016/j.ijmm.2013.11.016	Environmental soil	18.395300	98.678300
		18.642700	98.927200
		18.591800	99.081800
		18.565200	99.108600
		18.579600	99.151900
		18.551600	100.785000
		18.179300	99.467200
		18.177300	99.470400
		18.177300	99.470300
		18.165800	99.498000
		20.020100	100.272000
		20.209900	100.377000
		20.258900	100.407000
		20.258200	100.406000
		18.541700	100.678000
Htun, Z. Mar, et al. (2021), 10.1016/j.mycmed.2020.101085 Full text links	Horse serum	14.474500	100.117700
		13.528300	99.813400
		14.101100	99.417900
		19.910500	99.840600
		19.215400	100.202400
		13.690400	101.078000
		13.361100	100.984700
		7.564500	99.623900

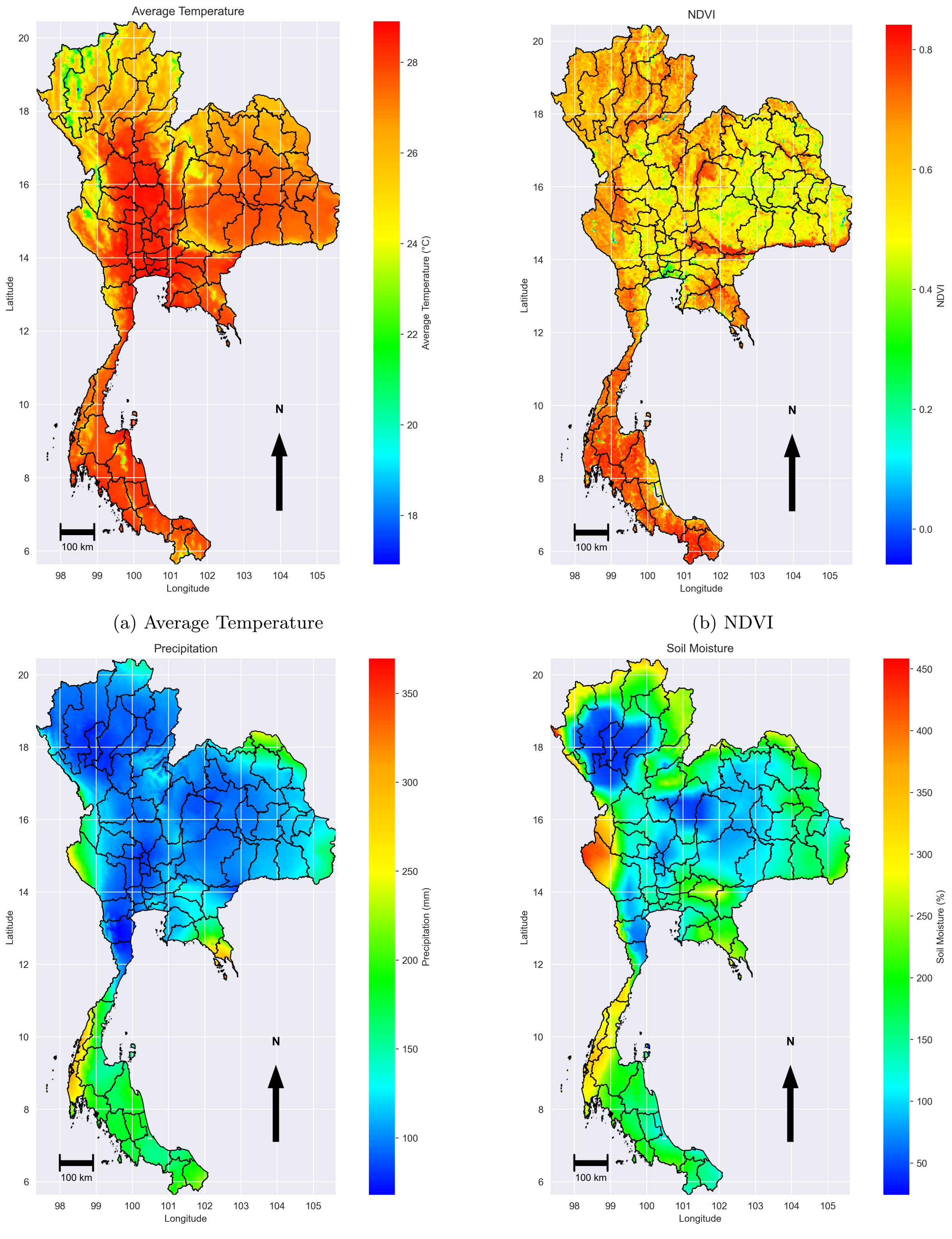

(a) Average Temperature (b) NDVI

(c) Precipitation (d) Soil Moisture

Figure S1: Environmental layers of (a) average temperature, (b) normalized difference vegetation index (NDVI), (c) precipitation, and (d) soil moisture index used as continuous predictors in the MaxEnt model. The maps in this figure were produced using Python version 3.12. Source of shapefile: United Nations Office for the Coordination of Humanitarian Affairs https://data.humdata.org/dataset/thailand-administrative-boundaries.

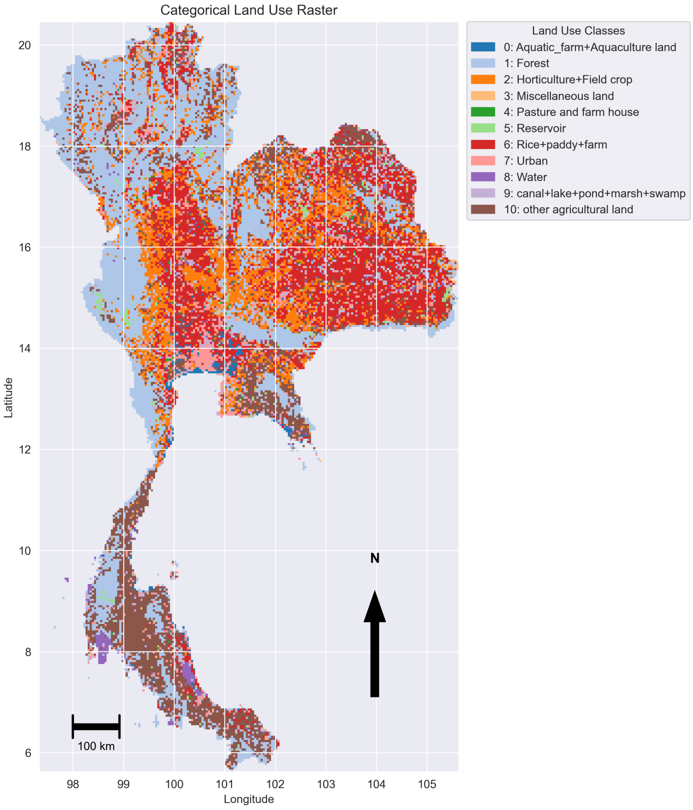

(a) Land use

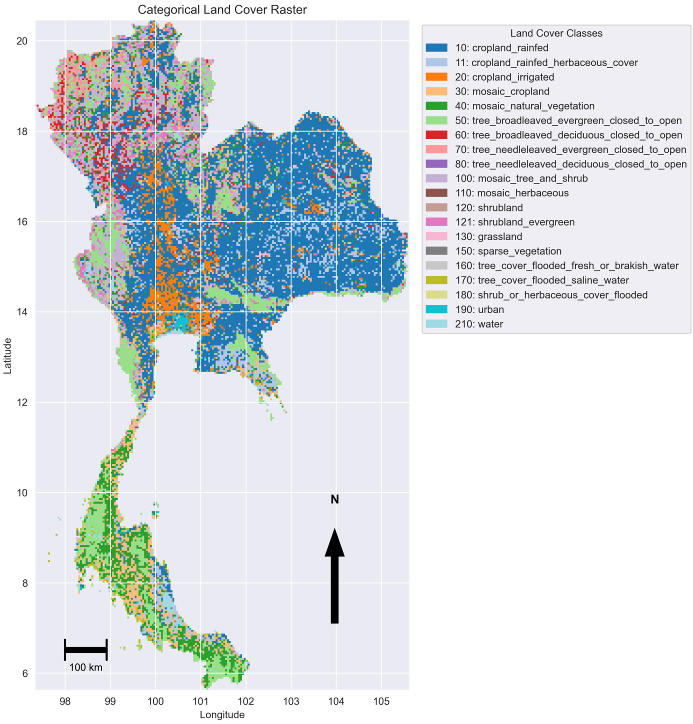

(b) Land cover

Figure S2: Input layers of (a) land use and (b) land cover variables used as categorical predictors in the MaxEnt model. The maps in this figure were produced using Python version 3.12. Source of shapefile: United Nations Office for the Coordination of Humanitarian Affairs https://data.humdata.org/dataset/thailand-administrative-boundaries.

Table S2: Land cover categories used in the MaxEnt model.

Code	Category description	Code	Category description
LC01	No data	LC20	Mosaic herbaceous
LC02	Cropland, rainfed	LC21	Shrubland
LC03	Cropland, rainfed, herbaceous cover	LC22	Shrubland, evergreen
LC04	Cropland, rainfed, tree or shrub cover	LC23	Shrubland, deciduous
LC05	Cropland, irrigated	LC24	Grassland
LC06	Mosaic cropland	LC25	Lichens and mosses
LC07	Mosaic natural vegetation	LC26	Sparse vegetation
LC08	Tree, broadleaved evergreen, closed to open	LC27	Sparse tree
LC09	Tree, broadleaved deciduous, closed to open	LC28	Sparse shrub
LC10	Tree, broadleaved deciduous, closed	LC29	Sparse herbaceous
LC11	Tree, broadleaved deciduous, open	LC30	Tree cover flooded, fresh or brackish water
LC12	Tree, needleleaved evergreen, closed to open	LC31	Tree cover flooded, saline water
LC13	Tree, needleleaved evergreen, closed	LC32	Shrub or herbaceous cover, flooded
LC14	Tree, needleleaved evergreen, open	LC33	Urban
LC15	Tree, needleleaved deciduous, closed to open	LC34	Bare areas
LC16	Tree, needleleaved deciduous, closed	LC35	Bare areas, consolidated
LC17	Tree, needleleaved deciduous, open	LC36	Bare areas, unconsolidated
LC18	Tree, mixed	LC37	Water
LC19	Mosaic tree and shrub		

Table S3: Land Use categories

Code	Category description	Code	Category description
LU01	Aquatic farm / aquaculture land	LU07	Rice, paddy, farm
LU02	Forest	LU08	Urban
LU03	Horticulture / field crop	LU09	Water
LU04	Miscellaneous land	LU10	Canal, lake, pond, marsh, swamp
LU05	Pasture and farm house	LU11	Other agricultural land
LU06	Reservoir		

Table S4: Variance inflation factor (VIF) values for environmental predictors included in the ZIP model. All variables had VIF values below 2.5, indicating low collinearity. The high value for the intercept term is expected and does not affect model interpretation.

Variable	VIF Values
Precipitation	1.13
Temperature	1.23
Soil moisture	1.08
NDVI	1.59
Land use	1.73
Land cover	2.24
Constant	39.88

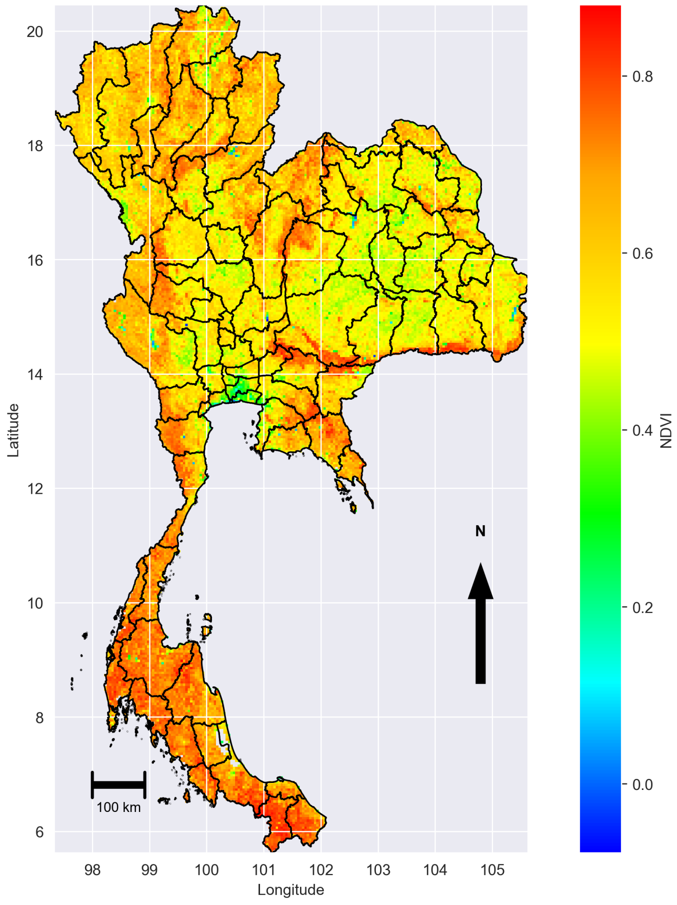

(a) NDVI in 2024

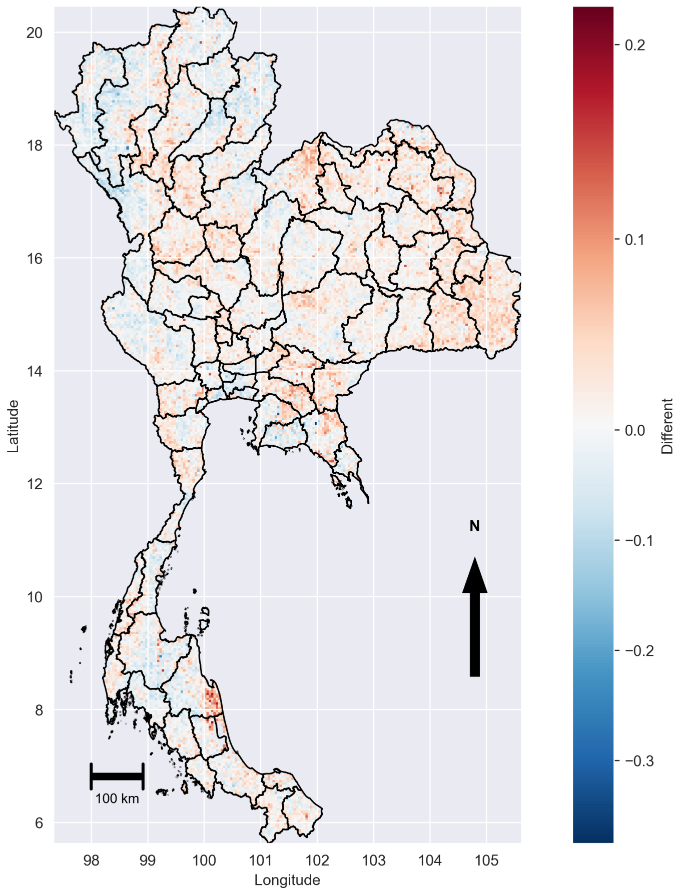

(b) Difference 2024 – baseline (2008–2020)

Figure S3: Normalized Difference Vegetation Index (NDVI). (a) 2024 annual mean, where lower values (blue shades) indicate less vegetation cover/greenness and higher values (green to yellow to red shades) indicate denser vegetation. (b) Difference relative to the 2008–2020 baseline (2024 minus baseline), where positive values (red shades) denote increased greenness/vegetation vigor and negative values (blue shades) indicate declines. The maps in this figure were produced using Python version 3.12. Source of shapefile: United Nations Office for the Coordination of Humanitarian Affairs https://data.humdata.org/dataset/thailand-administrative-boundaries.

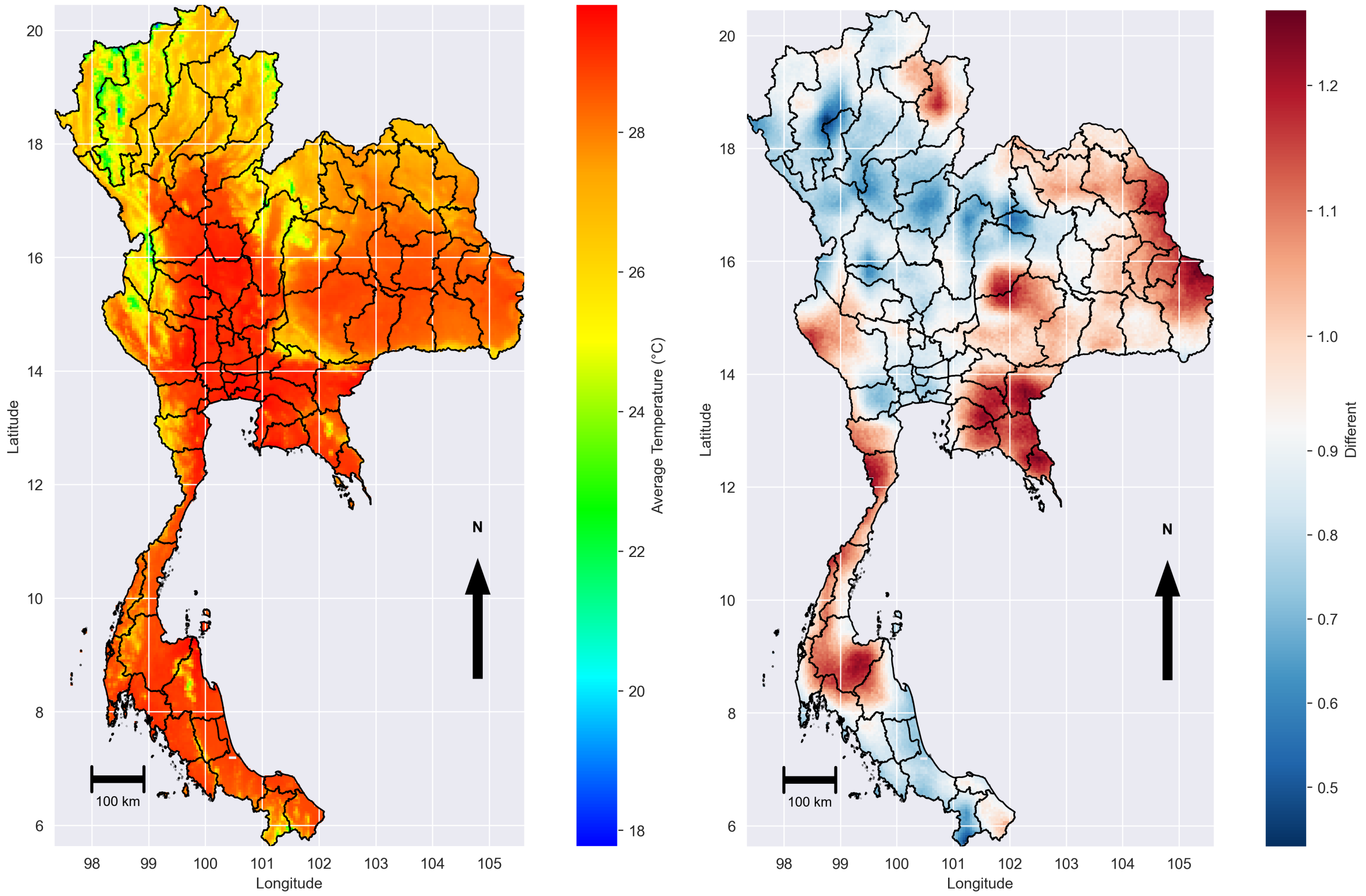

(a) Temperature average in 2024

(b) Difference 2024 – baseline (2008–2020)

Figure S4: Monthly mean of daily air temperature (TAVG, °C). (a) 2024 annual mean, where warmer colors (yellow to red) indicate higher temperatures and cooler colors (green to blue) indicate lower temperatures. (b) Differences in 2024 relative to the baseline period 2008–2020, where positive values (red shades) indicate warming and negative values (blue shades) indicate cooling. The maps in this figure were produced using Python version 3.12. Source of shapefile: United Nations Office for the Coordination of Humanitarian Affairs https://data.humdata.org/dataset/thailand-administrative-boundaries.

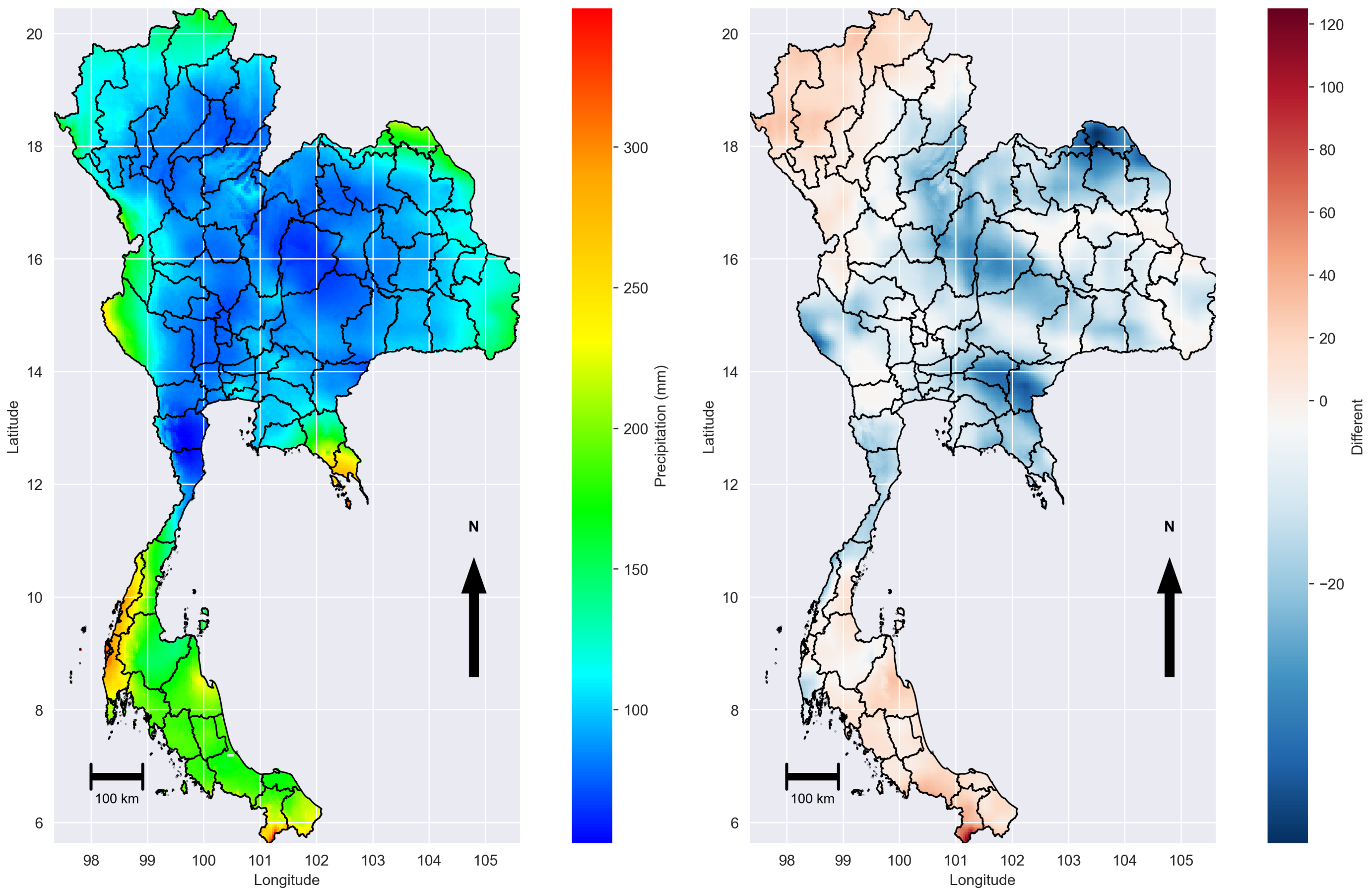

(a) Monthly precipitation in 2024

(b) Difference 2024 – baseline (2008–2020)

Figure S5: Total monthly precipitation summed to annual totals (PPT, mm). (a) 2024 annual total, where lower values are shown in blue shades and higher values in green to yellow to red shades. (b) Differences in 2024 relative to the baseline period 2008–2020, where positive values (red shades) indicate wetter conditions and negative values (blue shades) indicate drier conditions. The maps in this figure were produced using Python version 3.12. Source of shapefile: United Nations Office for the Coordination of Humanitarian Affairs https://data.humdata.org/dataset/thailand-administrative-boundaries.

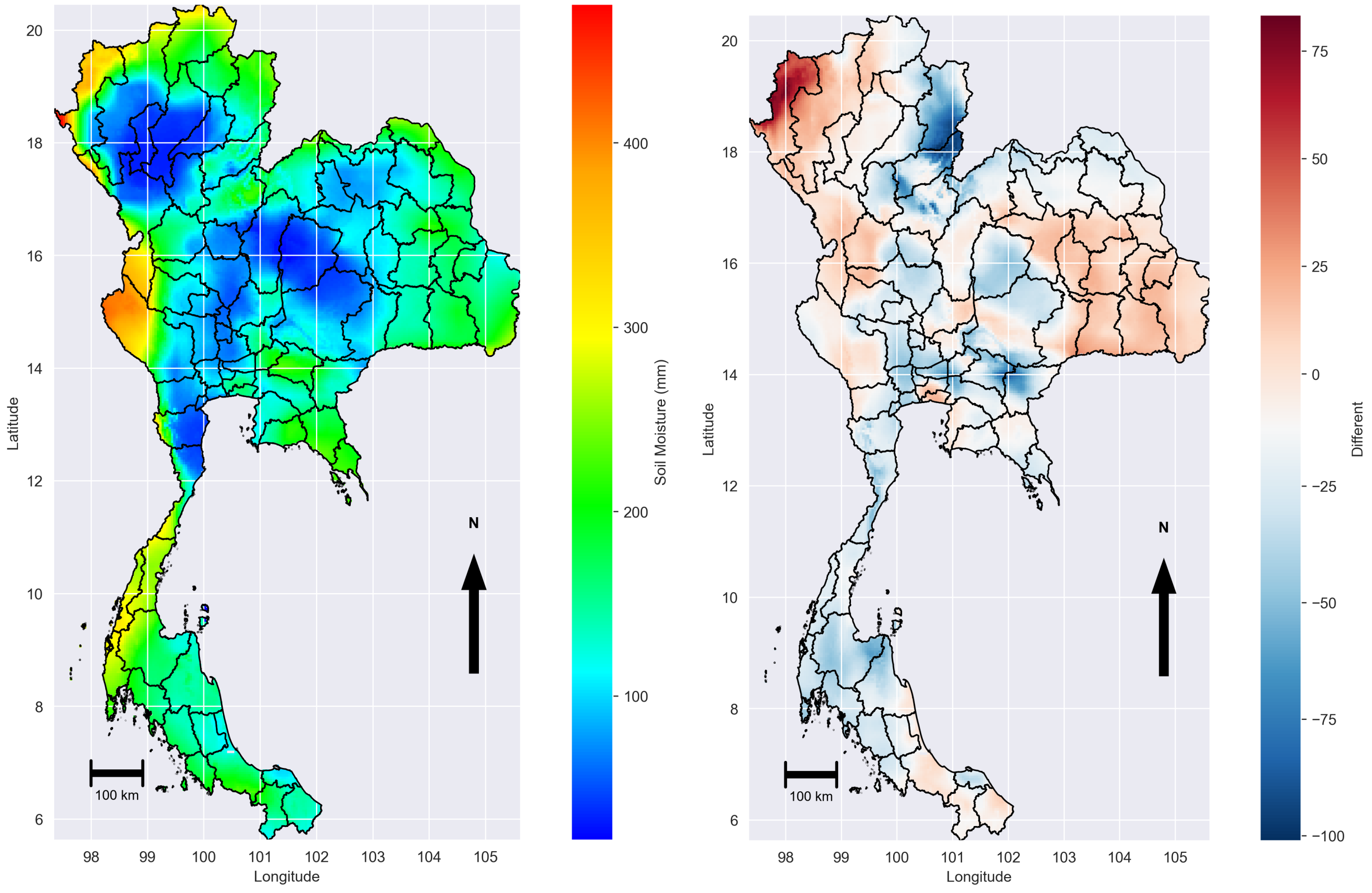

(a) Soil Moisture in 2024

(b) Difference 2024 – baseline (2008–2020)

Figure S6: Soil moisture. (a) 2024 annual mean (mm), where lower values are shown in blue shades and higher values in green to yellow to red shades. (b) Differences in 2024 relative to the baseline period 2008–2020, where positive values (red shades) denote increased soil moisture and negative values (blue shades) denote decreased soil moisture. The maps in this figure were produced using Python version 3.12. Source of shapefile: United Nations Office for the Coordination of Humanitarian Affairs https://data.humdata.org/dataset/thailand-administrative-boundaries.

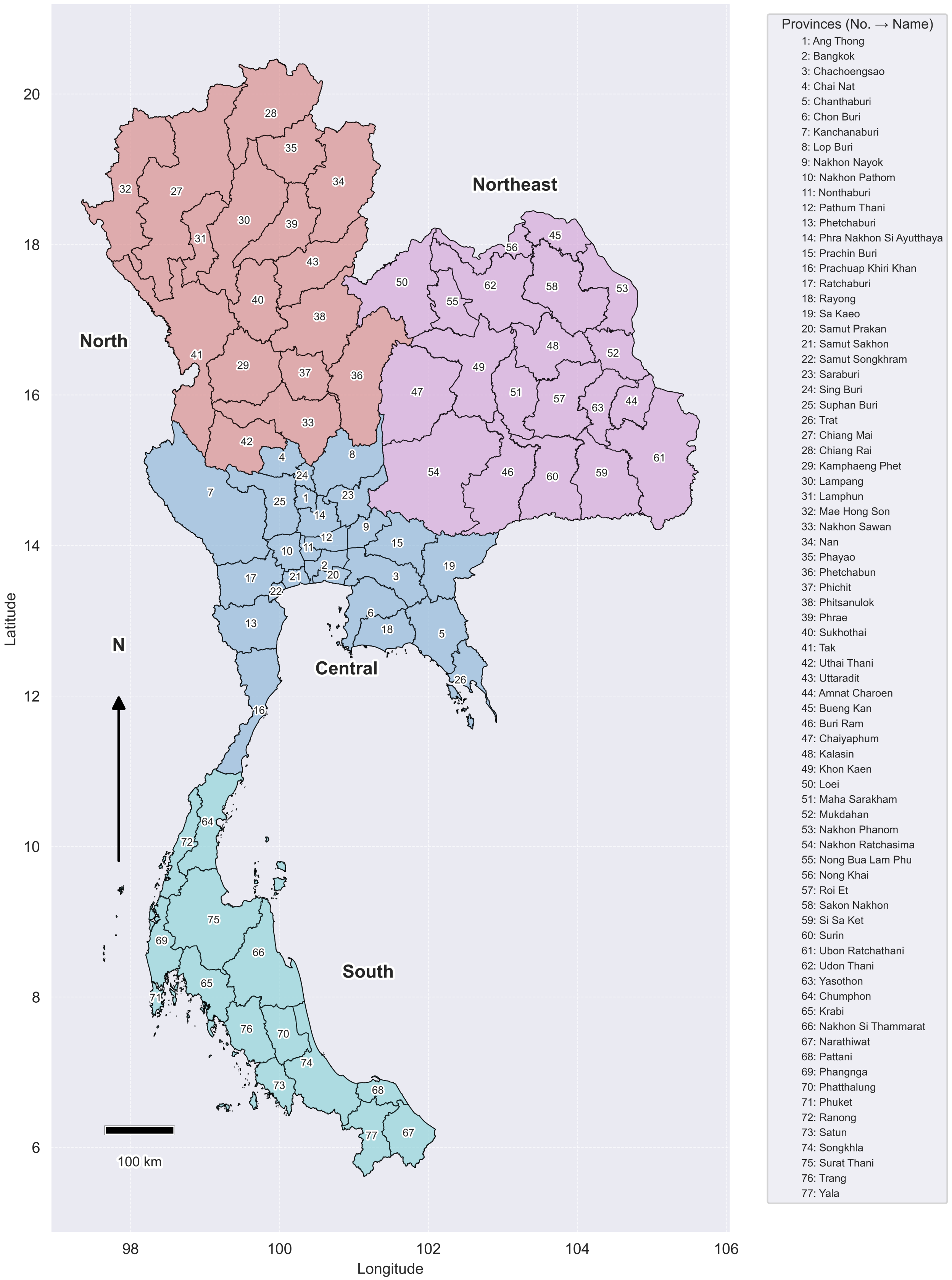

Figure S7: Map of Thailand showing provincial boundaries grouped into four regions: North (red), Northeast (purple), Central (blue), and South (teal). Provinces are numbered and listed in the panel on the right with corresponding names. The map in this figure was produced using Python version 3.12. Source of shapefile: United Nations Office for the Coordination of Humanitarian Affairs https://data.humdata.org/dataset/thailand-administrative-boundaries.

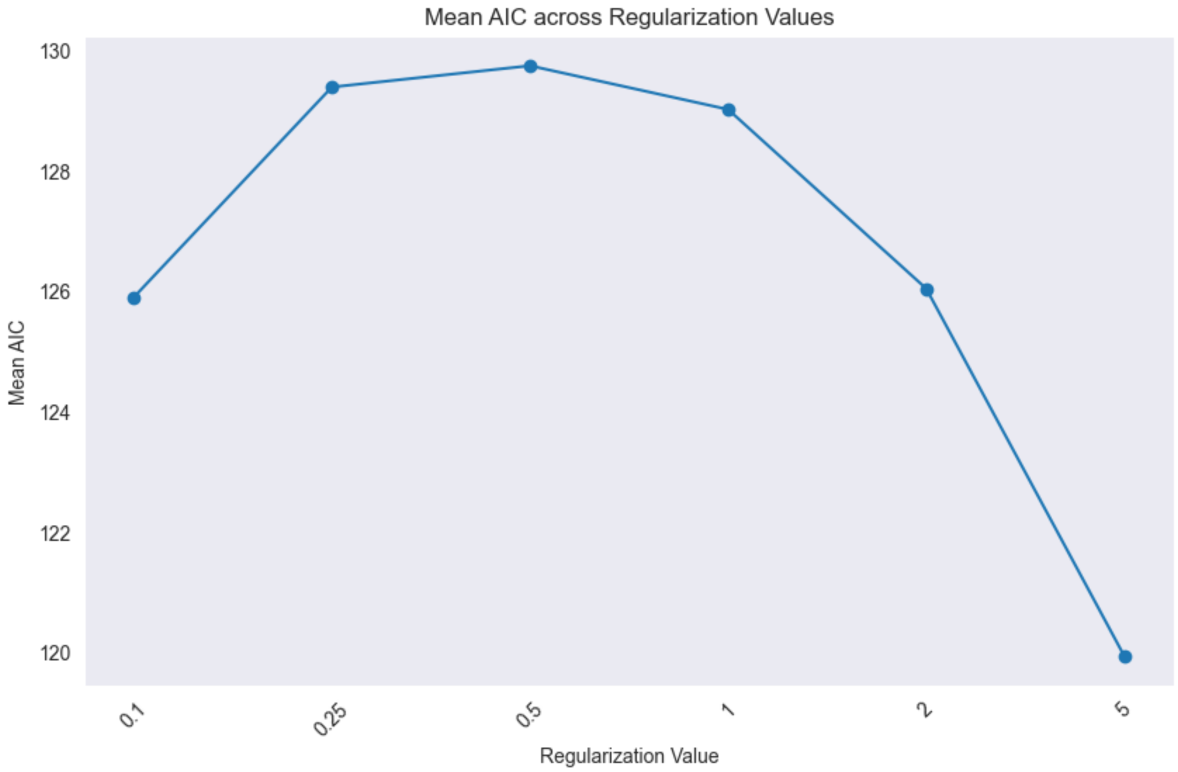

Figure S8: Mean AICc values for MaxEnt models with different regularization multipliers using the ZIP-filtered background. Although AICc was lowest at 0.5, improvement over 1.0 was minimal. The 1.0 setting produced smoother response curves and less variability across cross-validation replicates, indicating more stable predictions.

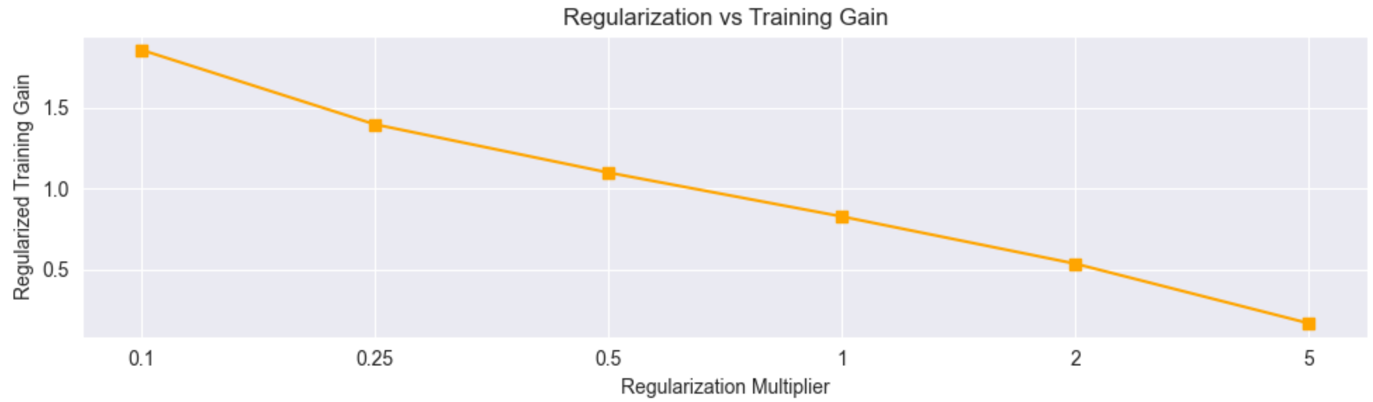

(a) Average training gain

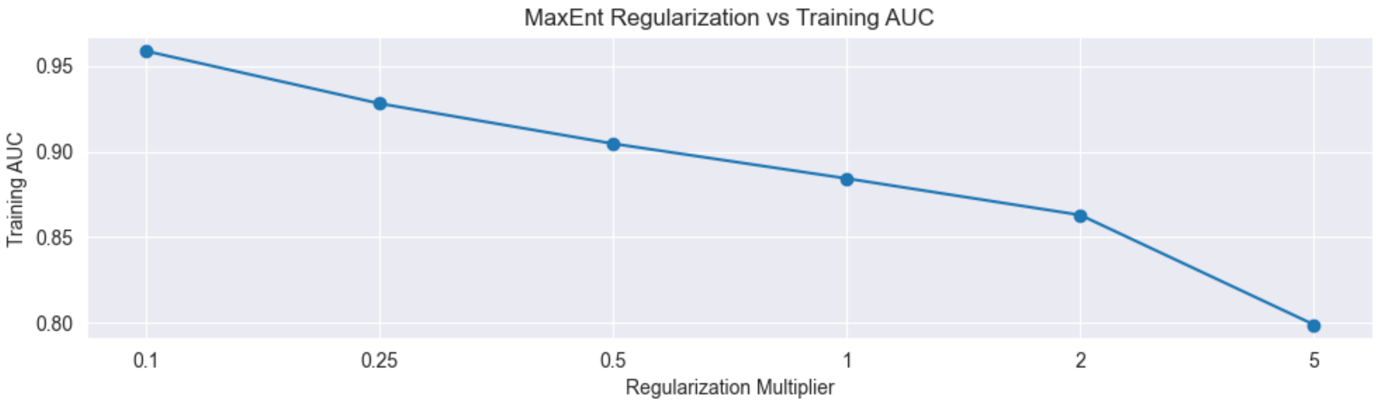

(b) Average training AUC

Figure S9: (a) Average training gain and (b) average training AUC across 10-fold cross-validation for MaxEnt models using linear features and a ZIP-filtered background. Both metrics remain stable with regularization values ranging from 0.1 to 5.0, with minor declines at higher values. The chosen setting of 1.0 balances predictive performance with smoother response behavior.

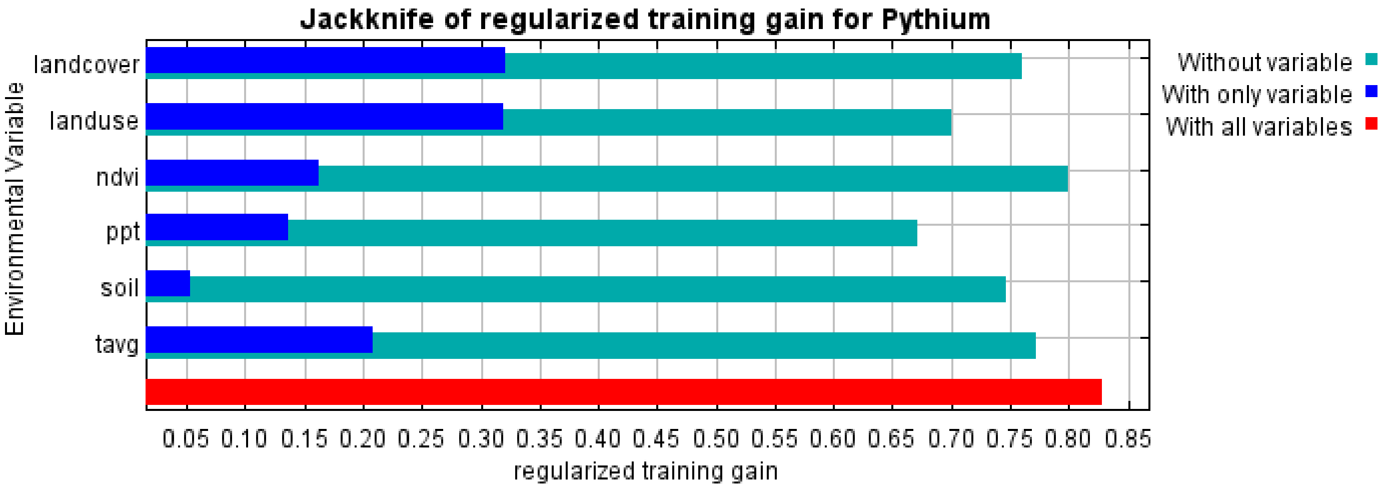

Figure S10: Jackknife analysis showing the contribution of each variable to regularized training gain. Land use and precipitation had the highest standalone gain and the greatest impact when omitted from the model.

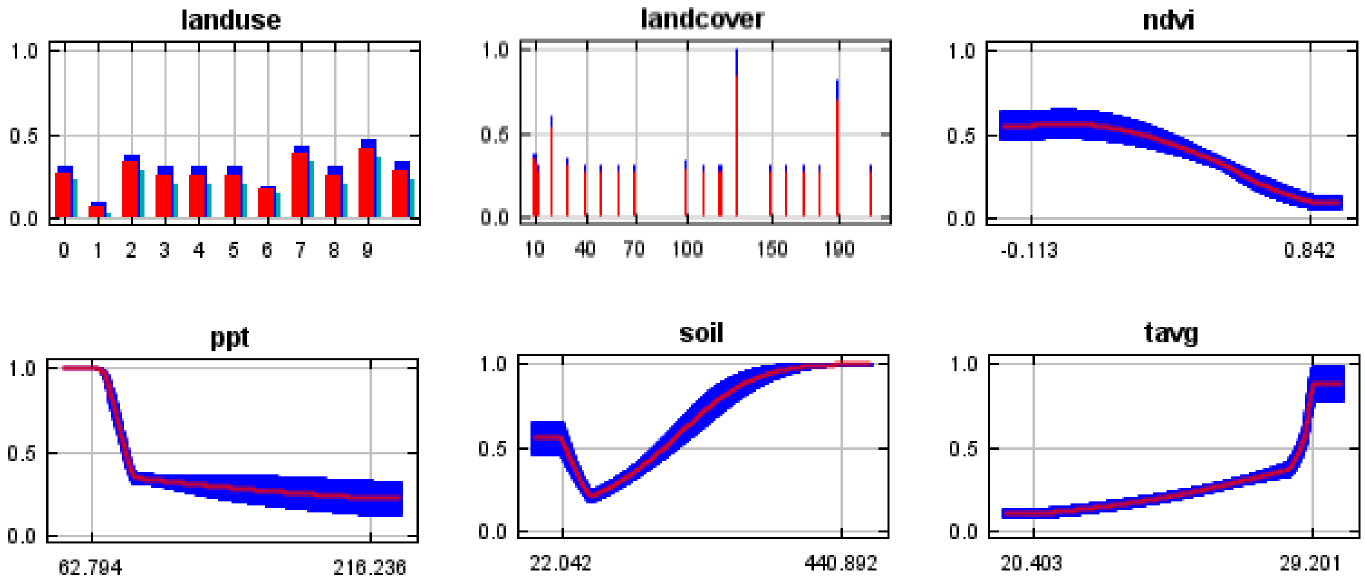

(a) Marginal curves

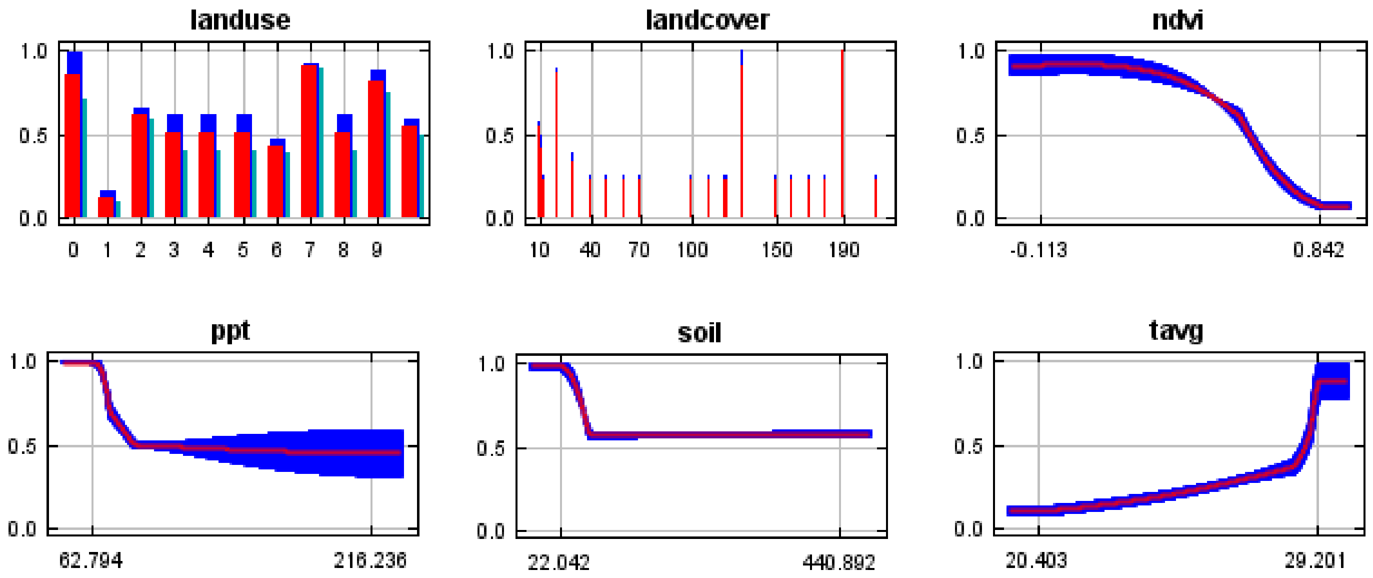

(b) Single-variable curves

Figure S11: Response curves for each predictor in the final MaxEnt model. Marginal curves (a) show suitability when all variables are included, while single-variable curves (b) isolate each predictor effect. Suitability declines with precipitation above 100 mm, rises sharply above 27 °C, varies across land use and land cover classes, decreases with increasing NDVI, and peaks for intermediate soil values. The differences between the marginal and single-variable curves reflect correlations among predictors.

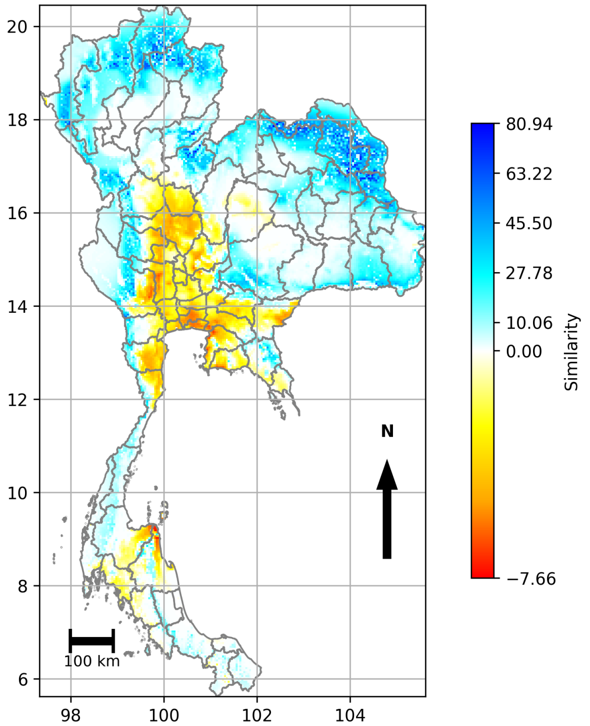

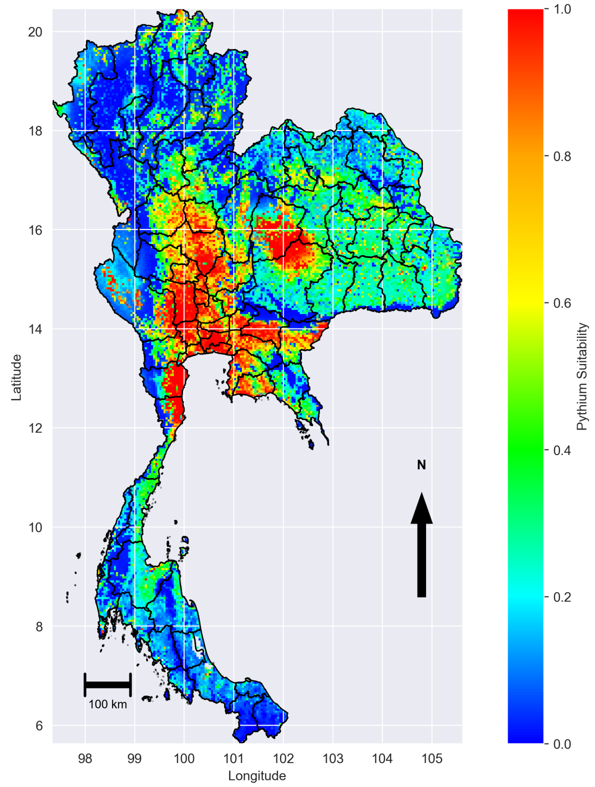

for

(a) MESS value

(b) 2024 suitability

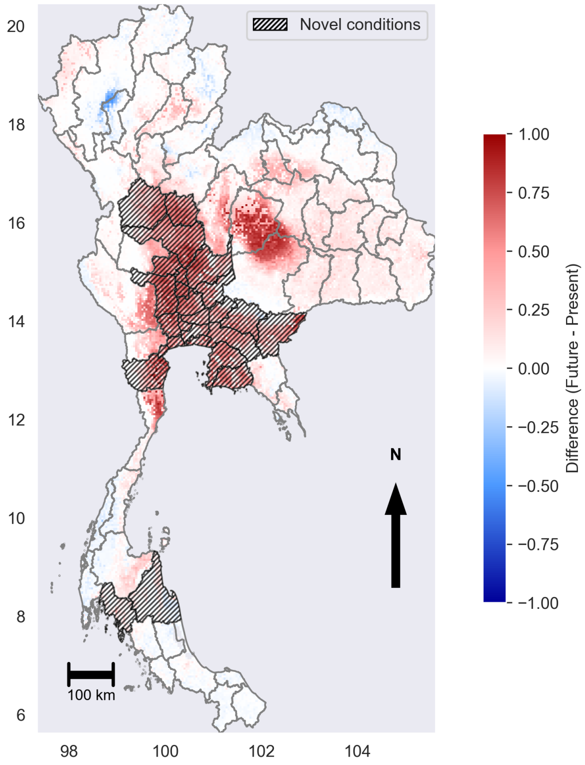

(c) Differences from baseline (future minus baseline)

Figure S12: Projected environmental suitability for *P. insidiosum* in 2024 relative to the 2008–2020 baseline: (a) MESS values, with only six out of seventy-seven provinces having more than 50% of their area classified as novel (MESS ≤ 0); (b) projected 2024 suitability across Thailand, with warmer colors indicating higher environmental favorability concentrated in the central plain; and (c) differences from the baseline, with warm colors showing increased suitability and cool colors decreased suitability, and hatched provinces indicating those where more than 50% of the area was novel. Maps were produced in Python 3.12 using shapefiles from the United Nations Office for the Coordination of Humanitarian Affairs (https://data.humdata.org/dataset/thailand-administrative-boundaries).

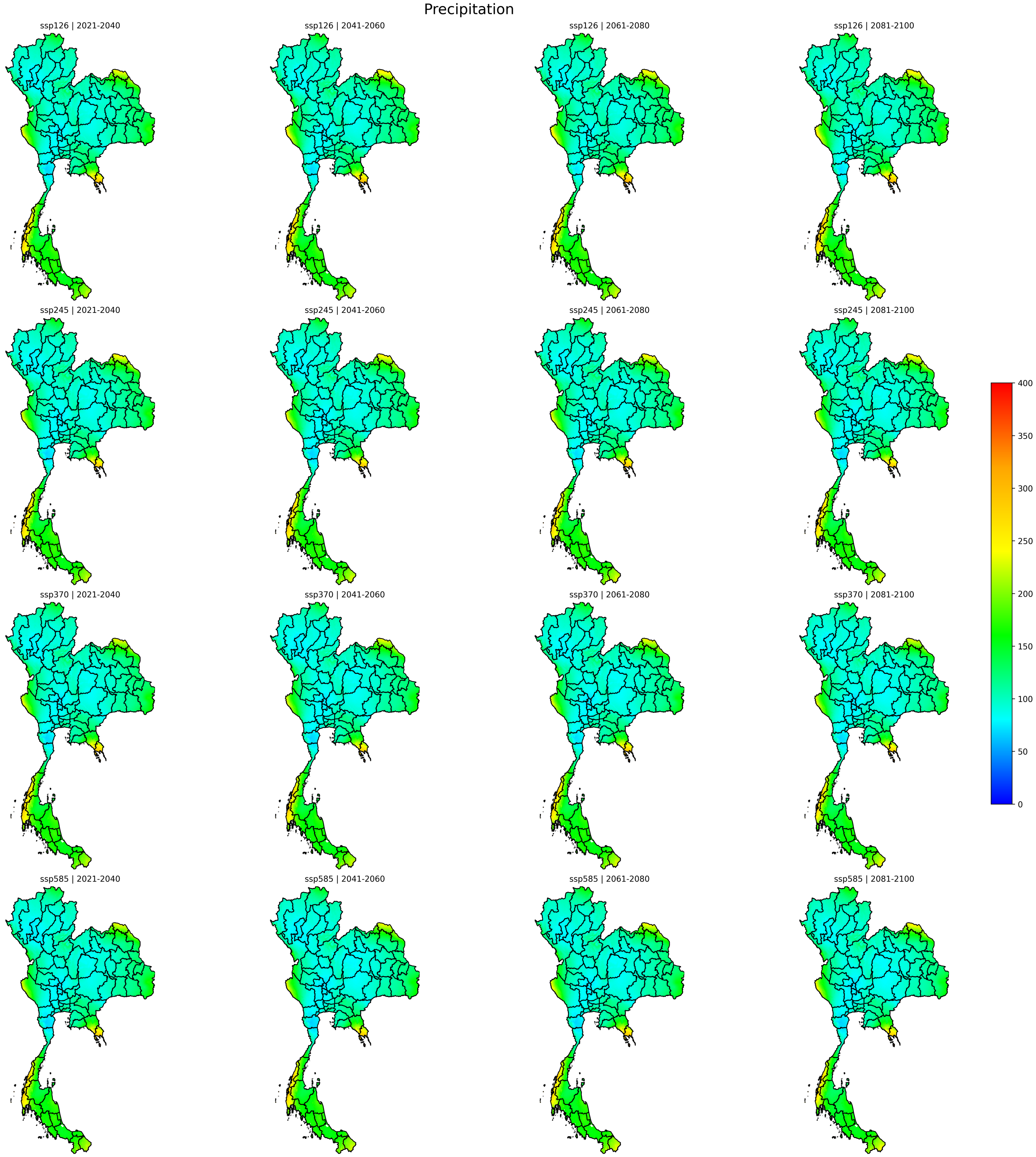

Figure S13: Projected annual precipitation (mm/year) across Thailand under SSP1–2.6, SSP2–4.5, SSP3–7.0, and SSP5–8.5 scenarios for four time periods: 2021–2040, 2041–2060, 2061–2080, and 2081–2100.The maps in this figure were produced using Python version 3.12. Source of shapefile: United Nations Office for the Coordination of Humanitarian Affairs https://data.humdata.org/dataset/thailand-administrative-boundaries.

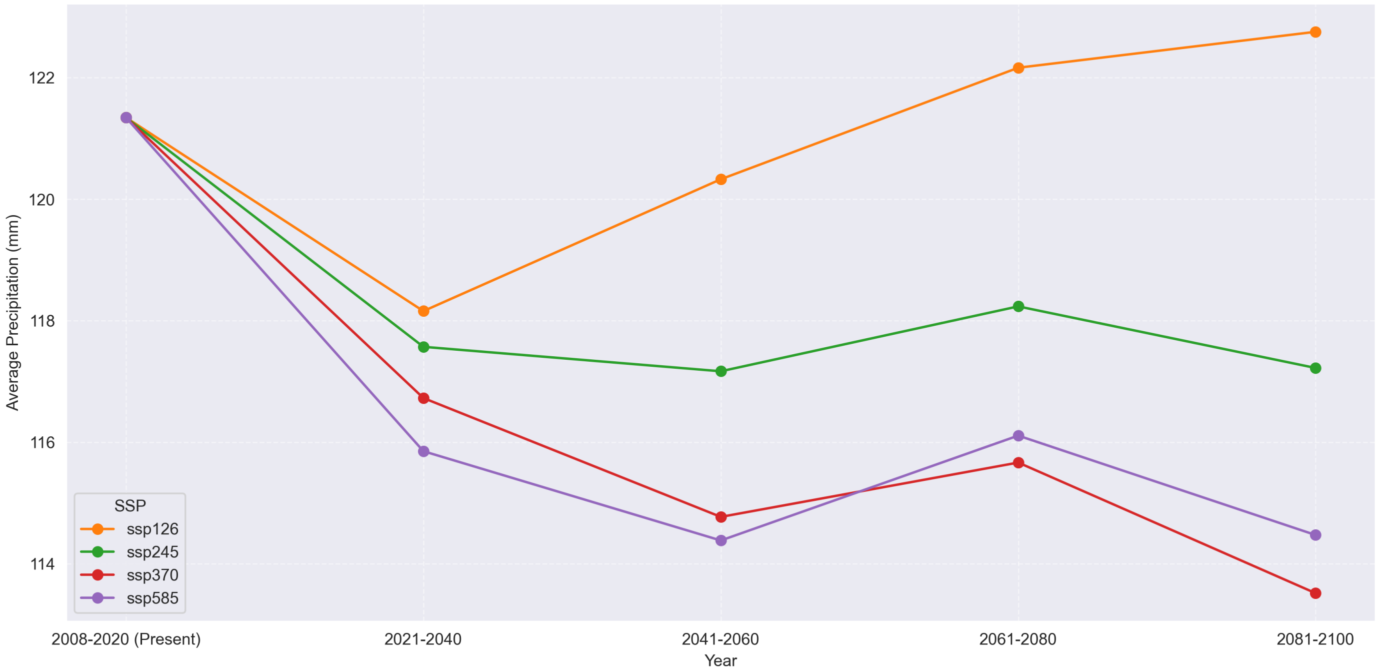

Figure S14: Trends in projected average monthly precipitation (mm) for Thailand under four SSP scenarios, 2008–2100. Values represent national means for each time period relative to the 2008–2020 baseline.

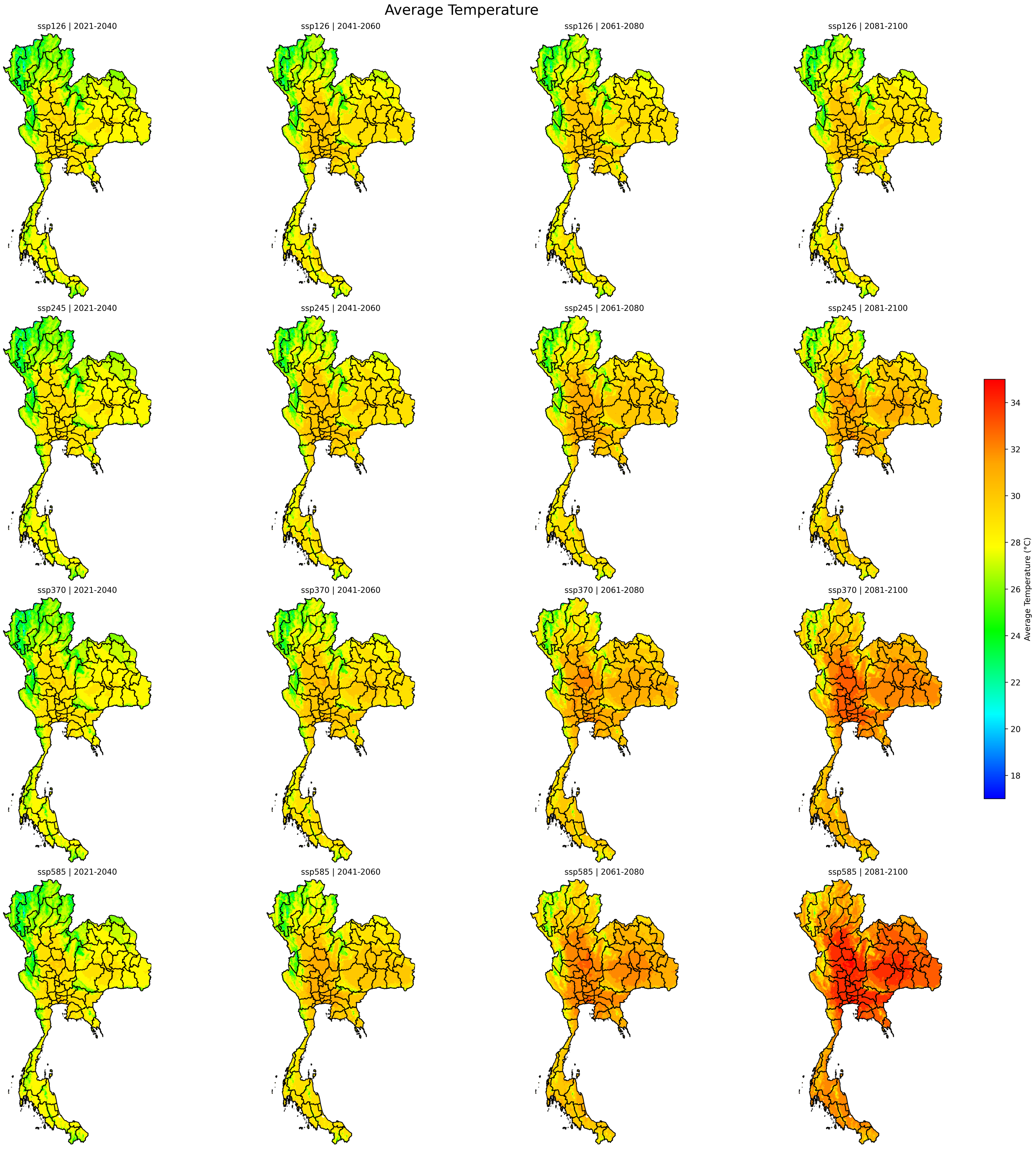

Figure S15: Projected annual mean temperature (°C) across Thailand under SSP1–2.6, SSP2–4.5, SSP3–7.0 and SSP5–8.5 scenarios for four time periods: 2021–2040, 2041–2060, 2061–2080, and 2081–2100. The maps in this figure were produced using Python version 3.12. Source of shapefile: United Nations Office for the Coordination of Humanitarian Affairs https://data.humdata.org/dataset/thailand-administrative-boundaries.

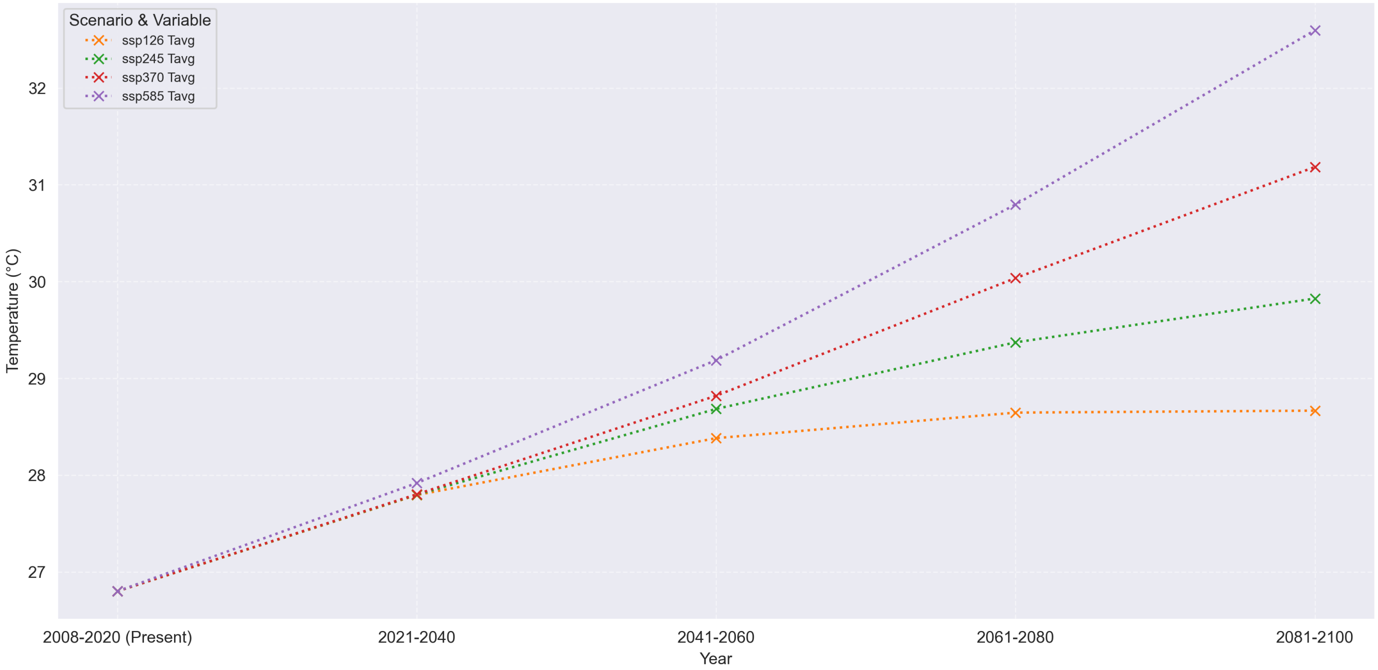

Figure S16: Trends in projected annual mean temperature (°C) for Thailand under four SSP scenarios, 2008–2100. Values represent national means for each time period relative to the 2008–2020 baseline.

1 SSP1-2.6

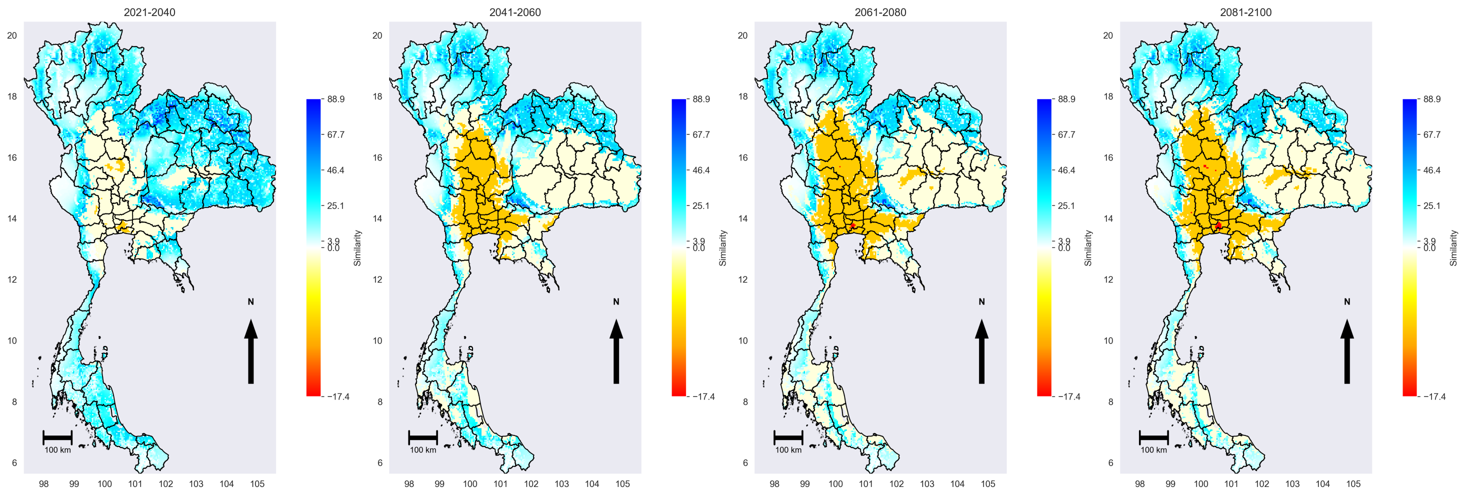

(a) MESS value

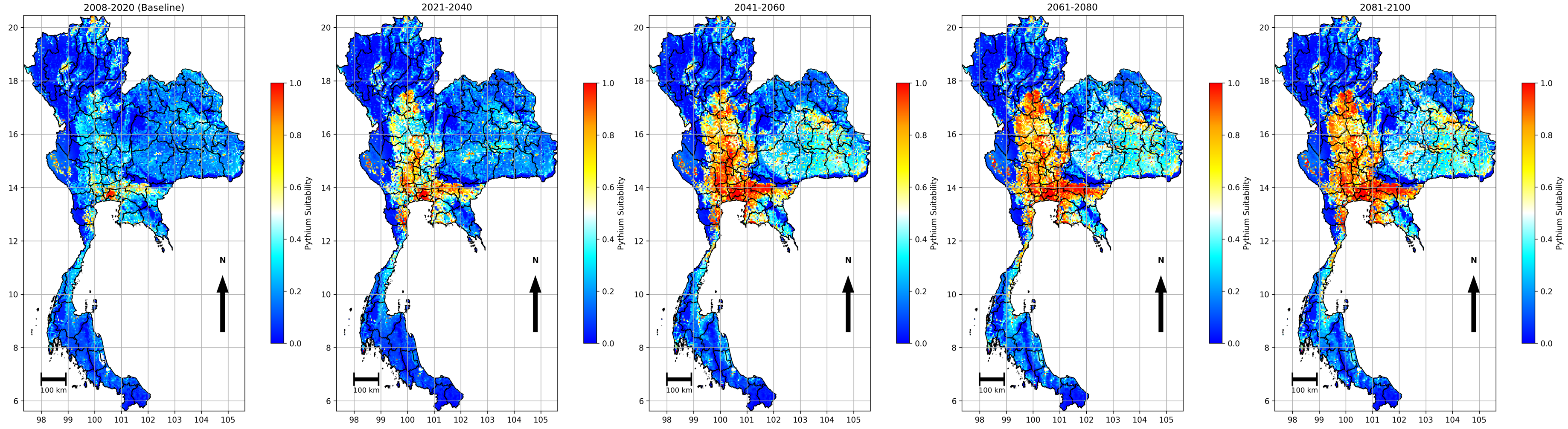

(b) Baseline suitability (leftmost) and projections for 2021–2040, 2041–2060, 2061–2080, and 2081–2100

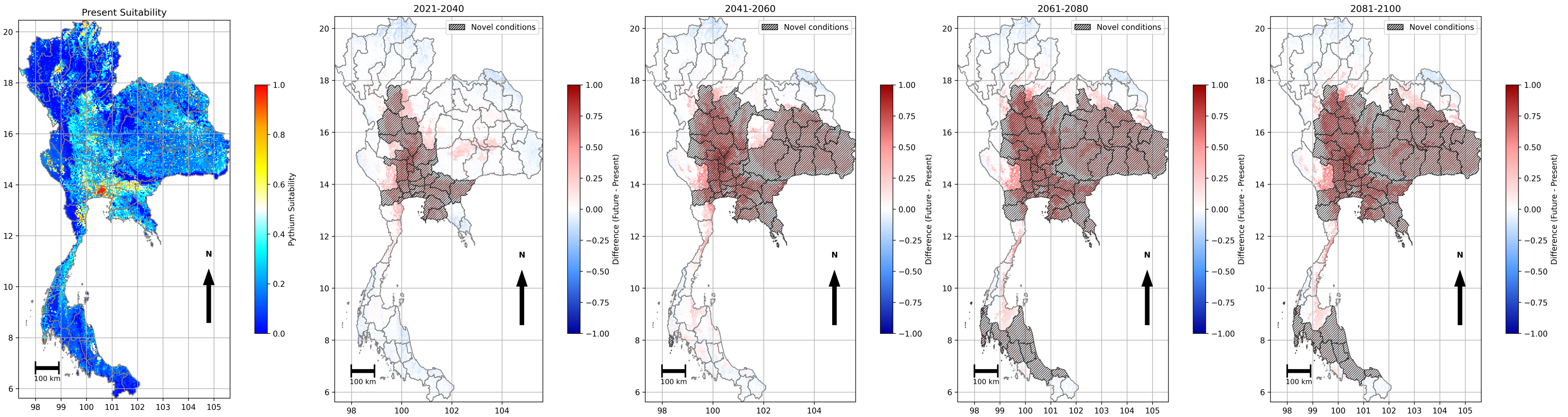

(c) Differences from baseline (future minus baseline)

Figure S17: Projected environmental suitability for *P. insidiosum* under SSP1–2.6 relative to the 2008–2020 baseline: (a) MESS values, where positive values (MESS > 0) indicate environments within the training range and negative values (MESS ≤ 0) indicate novel conditions; by 2021–2040 novel conditions were evident in only about 12 provinces (15.6%), but expanded gradually to reach roughly two-thirds of provinces by 2100, while several northern highland provinces remained less affected; (b) baseline suitability and projections for 2021–2100, with warm colors showing consistently high favorability in the central plain and cool colors lower suitability; and (c) differences from the baseline, where warm colors show increases, cool colors decreases, and hatching highlights provinces where novel conditions covered more than half of the area. Maps were produced in Python 3.12 using shapefiles from the United Nations Office for the Coordination of Humanitarian Affairs (https://data.humdata.org/dataset/thailand-administrative-boundaries).

2 SSP2-4.5

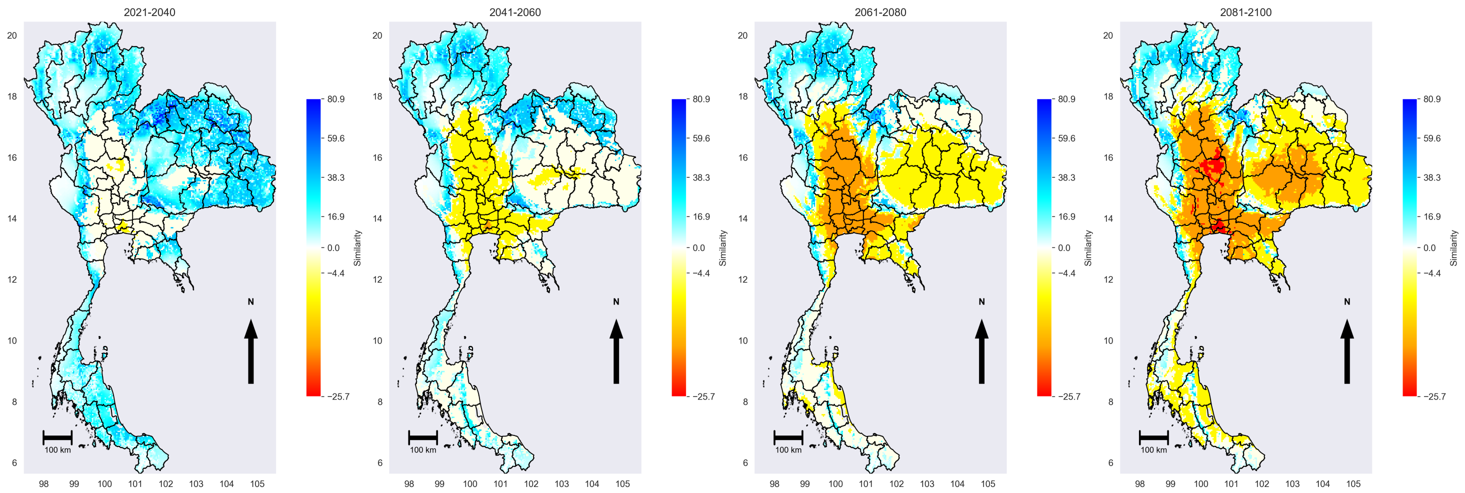

(a) MESS value

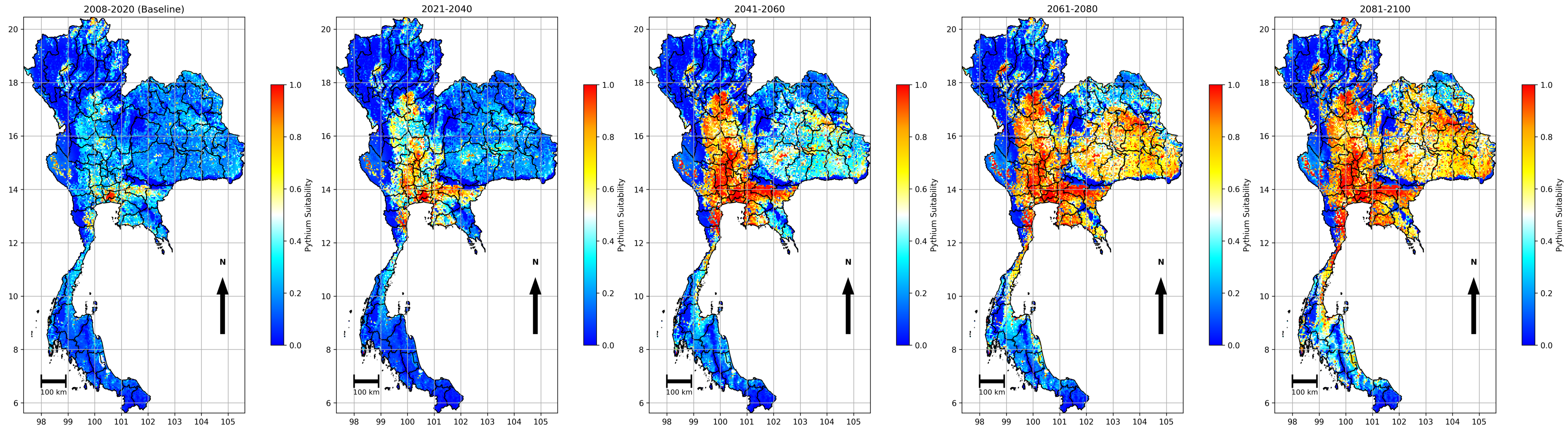

(b) Baseline suitability (leftmost) and projections for 2021–2040, 2041–2060, 2061–2080, and 2081–2100

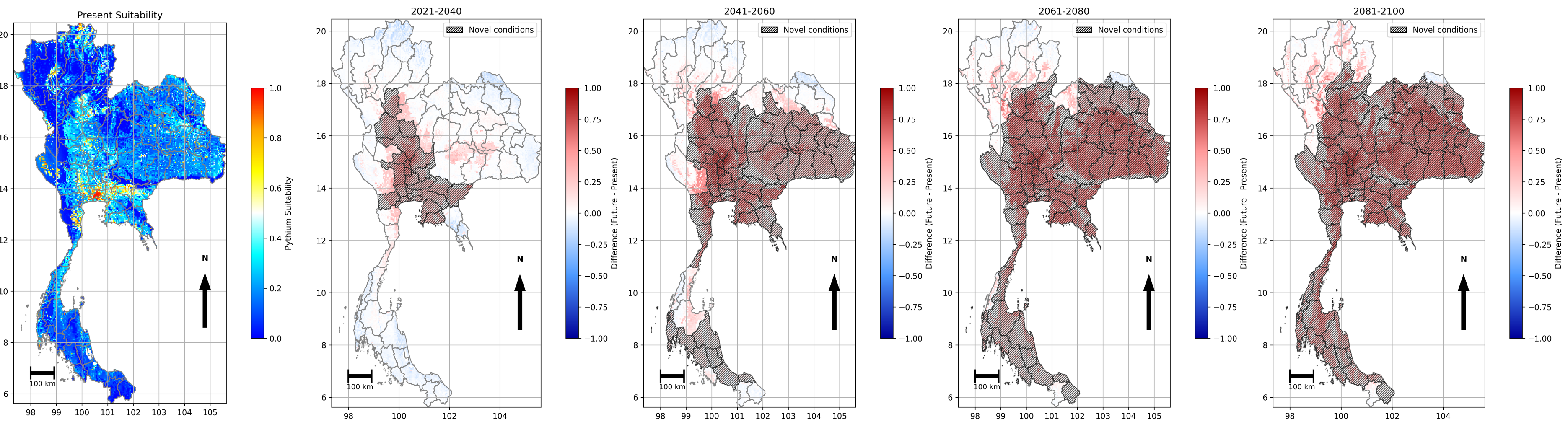

(c) Differences from baseline (future minus baseline)

Figure S18: Projected environmental suitability for *P.insidiosum* under SSP2–4.5 relative to the 2008–2020 baseline: (a) MESS values, where positive values (MESS > 0) indicate environments within the training range and negative values (MESS ≤ 0) indicate novel conditions; these emerged by 2021–2040, spread to over half of provinces by mid-century, and extended to nearly all by 2100; (b) baseline suitability and projections for 2021–2100, with warm colors showing widespread and intensifying favorability in central and northeastern provinces, while cooler colors mark persistently lower suitability in northern highlands; and (c) differences from the baseline, where warm colors show increases, cool colors decreases, and hatching indicate provinces where novel conditions covered more than half of the area. Maps were produced in Python 3.12 using shapefiles from the United Nations Office for the Coordination of Humanitarian Affairs (https://data.humdata.org/dataset/thailand-administrative-boundaries).

3 SSP3–7.0

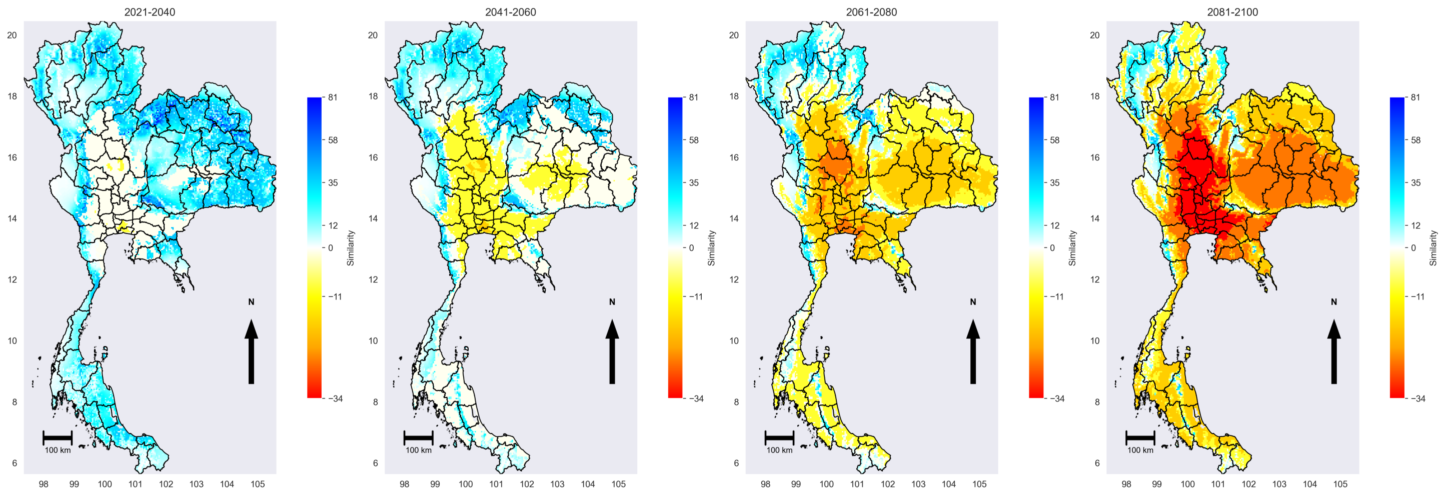

(a) MESS value

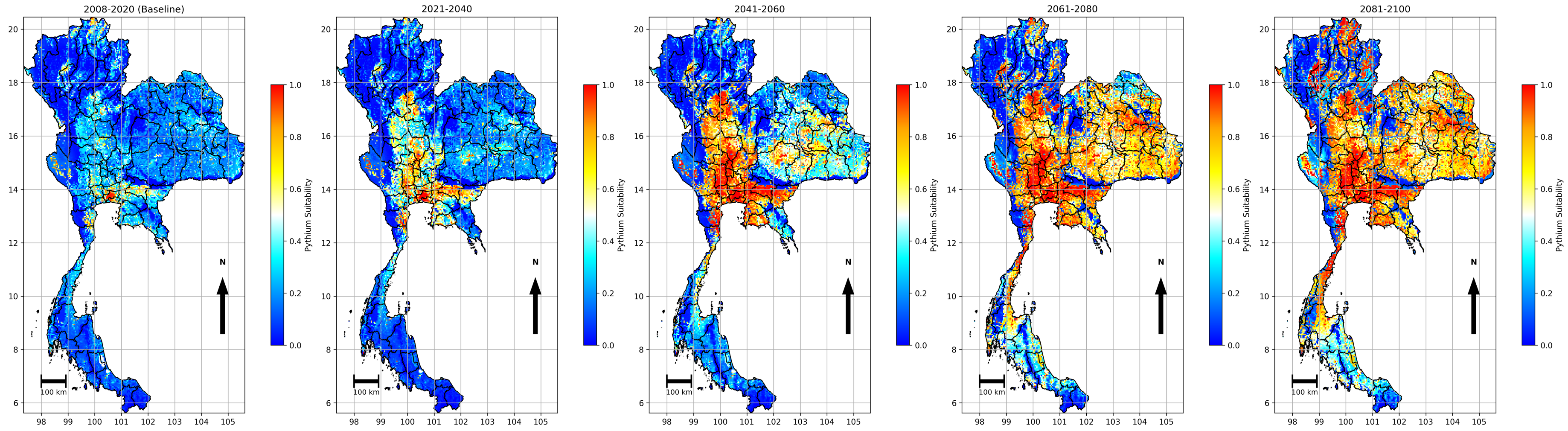

(b) Baseline suitability (leftmost) and projections for 2021–2040, 2041–2060, 2061–2080, and 2081–2100

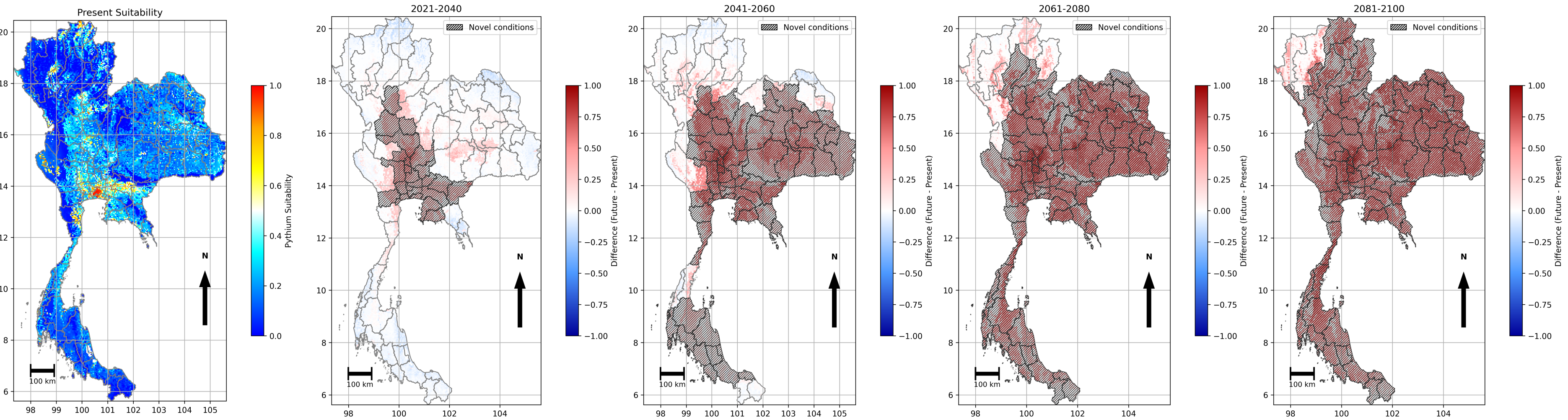

(c) Differences from baseline (future minus baseline)

Figure S19: Projected environmental suitability for P.insidiosum under SSP3–7.0 relative to the 2008–2020 baseline: (a) MESS values, where positive values (MESS > 0) indicate environments within the training range and negative values (MESS ≤ 0) indicate novel conditions; these became widespread by 2021–2040, expanded to a majority of provinces by mid-century, and extended to nearly all by 2100, with particularly strong signals across central and northeastern regions; (b) baseline suitability and projections for 2021–2100, with warm colors showing extensive and intensifying suitability throughout most lowland and northeastern provinces, and cooler colors marking persistently lower suitability in northern highlands; and (c) differences from the baseline, where warm colors indicate increases, cool colors decreases, and hatching indicate provinces where novel conditions covered more than half of the area. Maps were produced in Python 3.12 using shapefiles from the United Nations Office for the Coordination of Humanitarian Affairs (https://data.humdata.org/dataset/thailand-administrative-boundaries).

4 SSP5-8.5

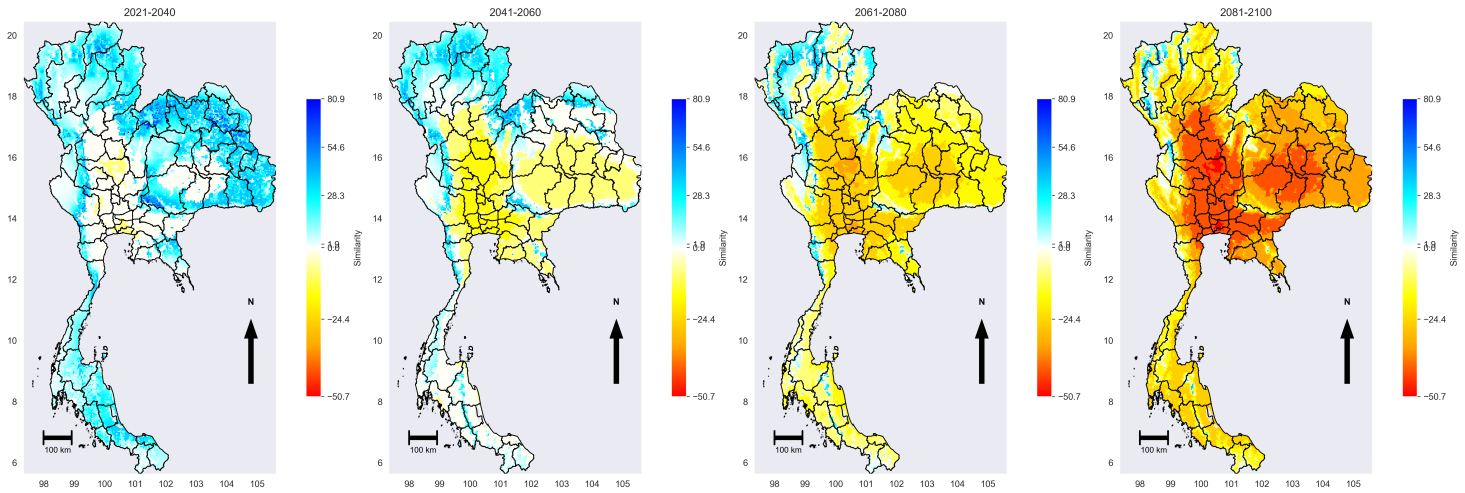

(a) MESS value

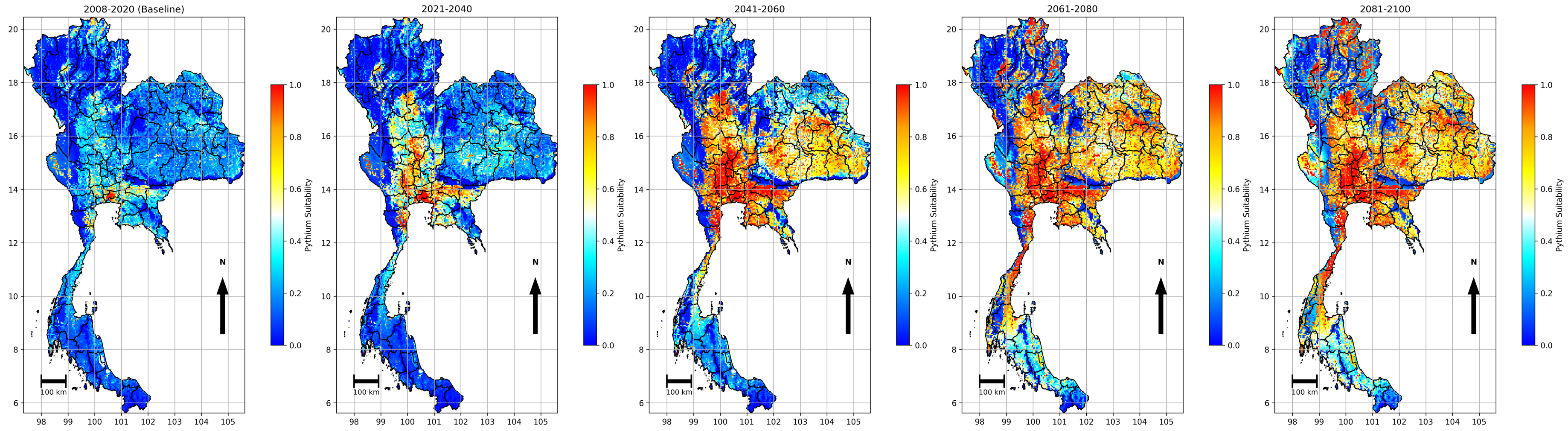

(b) Baseline suitability (leftmost) and projections for 2021–2040, 2041–2060, 2061–2080, and 2081–2100

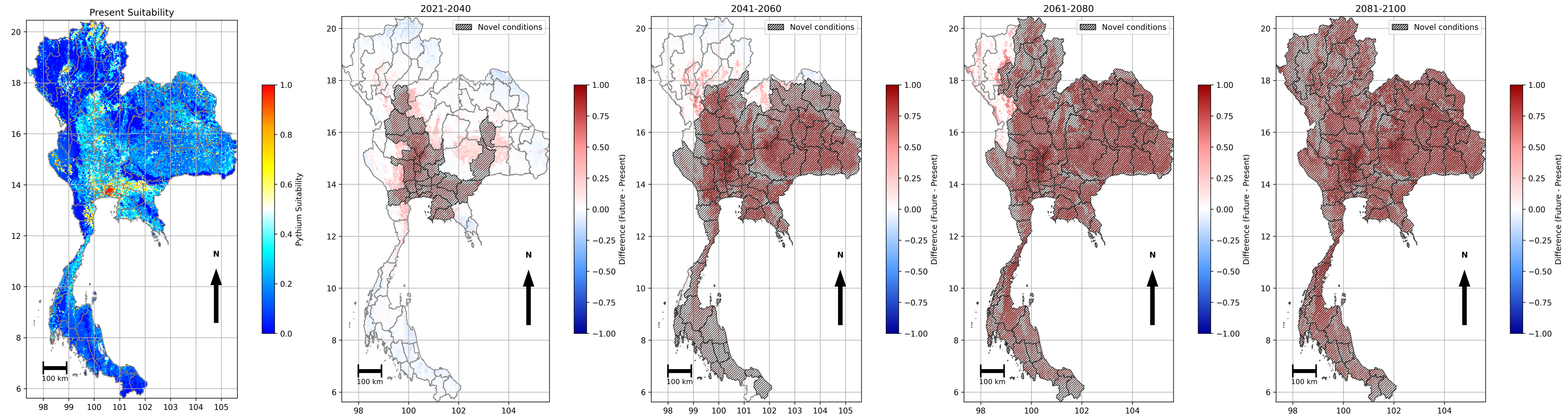

(c) Differences from baseline (future minus baseline)

Figure S20: Projected environmental suitability for *P. insidiosum* under SSP5–8.5 relative to the 2008–2020 baseline: (a) MESS values, where positive values (MESS > 0) indicate environments within the training range and negative values (MESS ≤ 0) indicate novel conditions; these expanded most rapidly, with many provinces already showing widespread novel conditions by 2021–2040, the majority exceeding 70–80% by mid-century, more than seventy provinces ($>$90%) surpassing 80% by 2061–2080, and all seventy-seven provinces reaching $>$90–100% by 2100; (b) baseline suitability and projections for 2021–2100, with warm colors indicating increasingly high suitability across central, northeastern, and lowland regions, and cool colors denoting persistently lower suitability in highlands and southern fringes; and (c) differences from the baseline, where warm colors indicate increases, cool colors decreases, and hatching indicate provinces where novel conditions covered more than half of the area. Maps were produced in Python 3.12 using shapefiles from the United Nations Office for the Coordination of Humanitarian Affairs (https://data.humdata.org/dataset/thailand-administrative-boundaries)

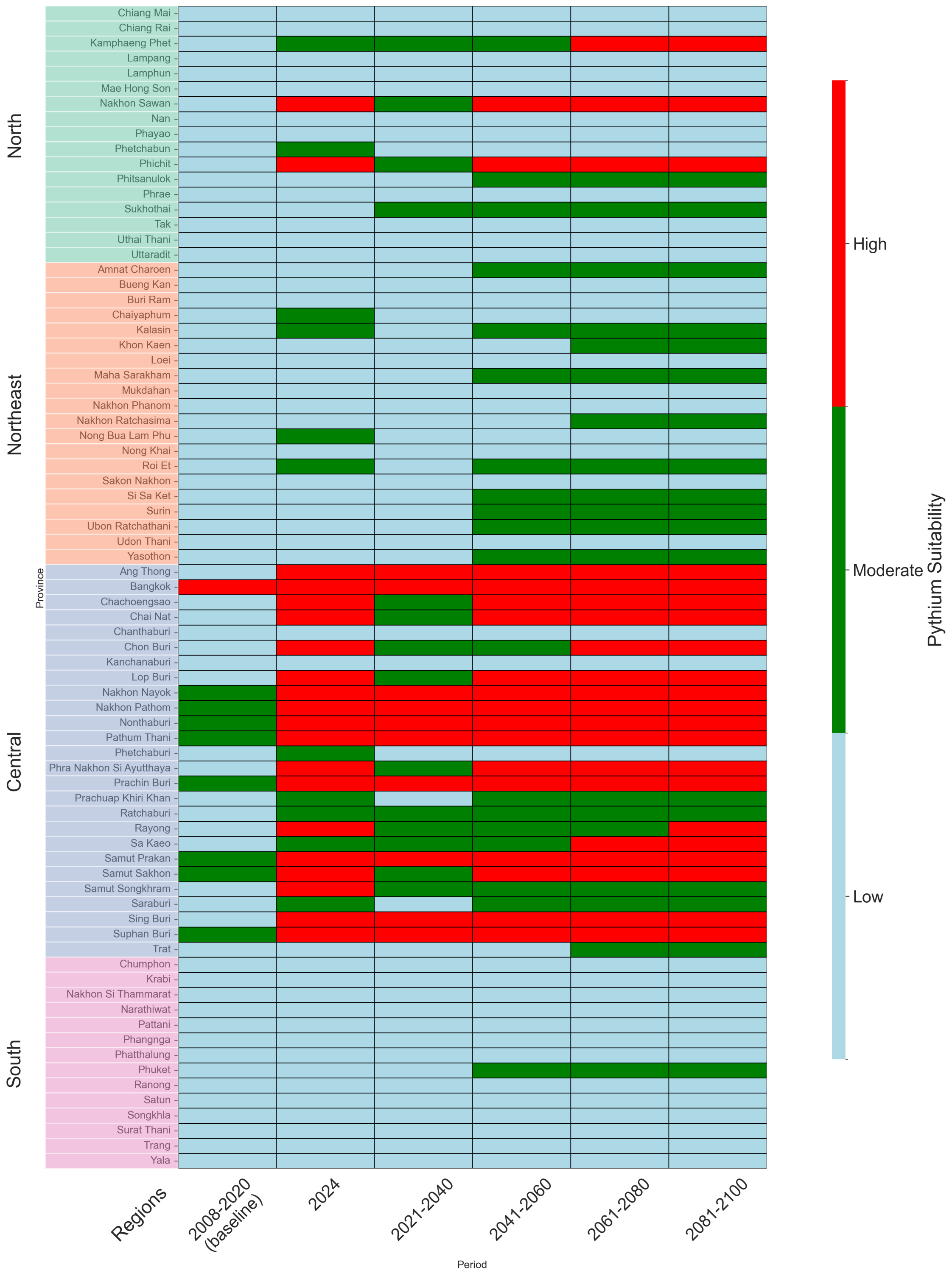

Figure S21: Provincial suitability distributions at baseline, near-current projection (2024), and long-term projection for 2021–2040, 2041–2060, 2061–2080, and 2081–2100 under SSP1-2.6, showing threshold-based category shifts with provinces classified into high (⩾0.60), moderate (0.30–0.59), and low (<0.30) suitability.

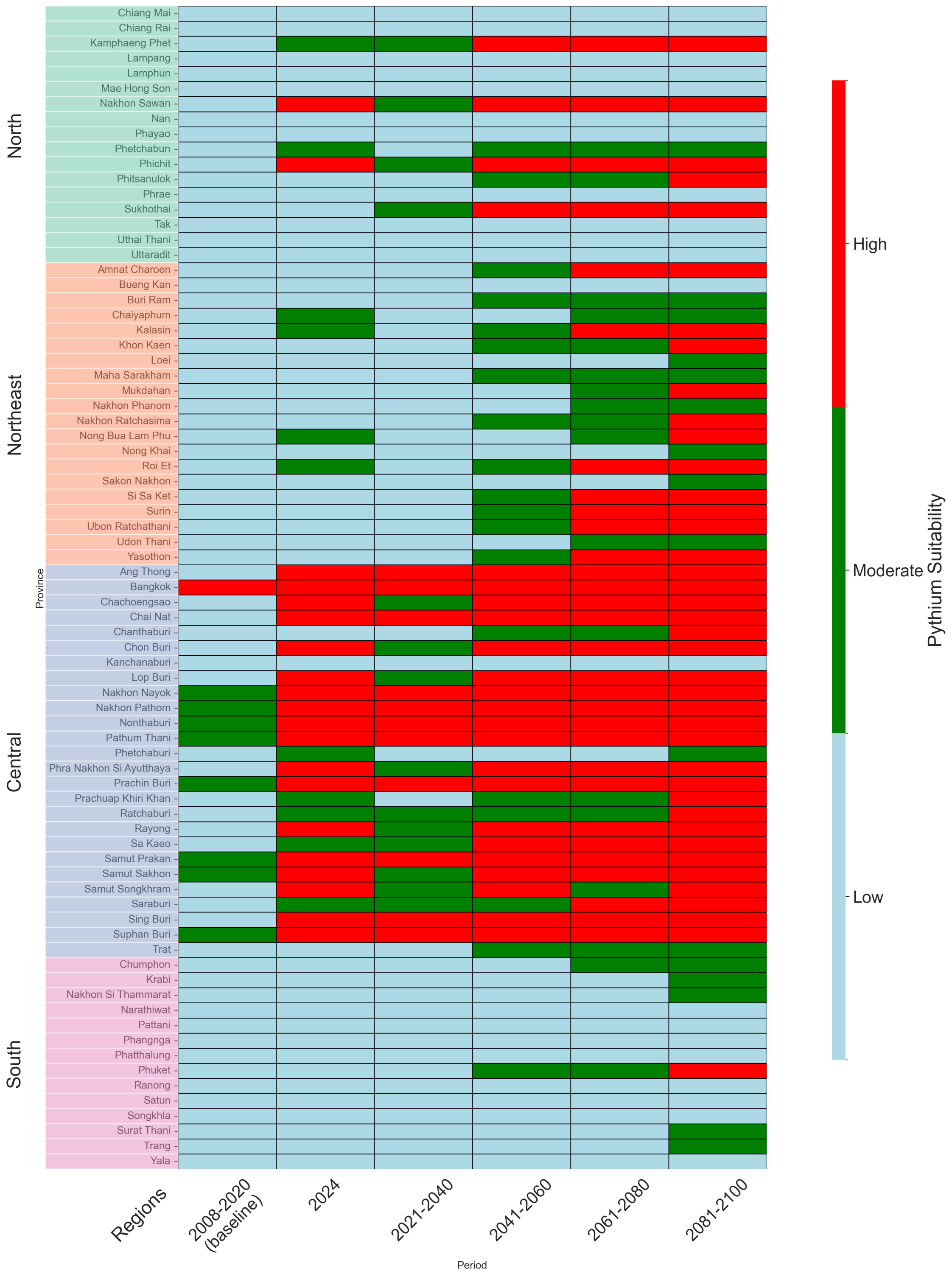

Figure S22: Provincial suitability distributions at baseline, near-current projection (2024), and long-term projection for 2021–2040, 2041–2060, 2061–2080, and 2081–2100 under SSP2-4.5, showing threshold-based category shifts with provinces classified into high (⩾0.60), moderate (0.30–0.59), and low (<0.30) suitability.

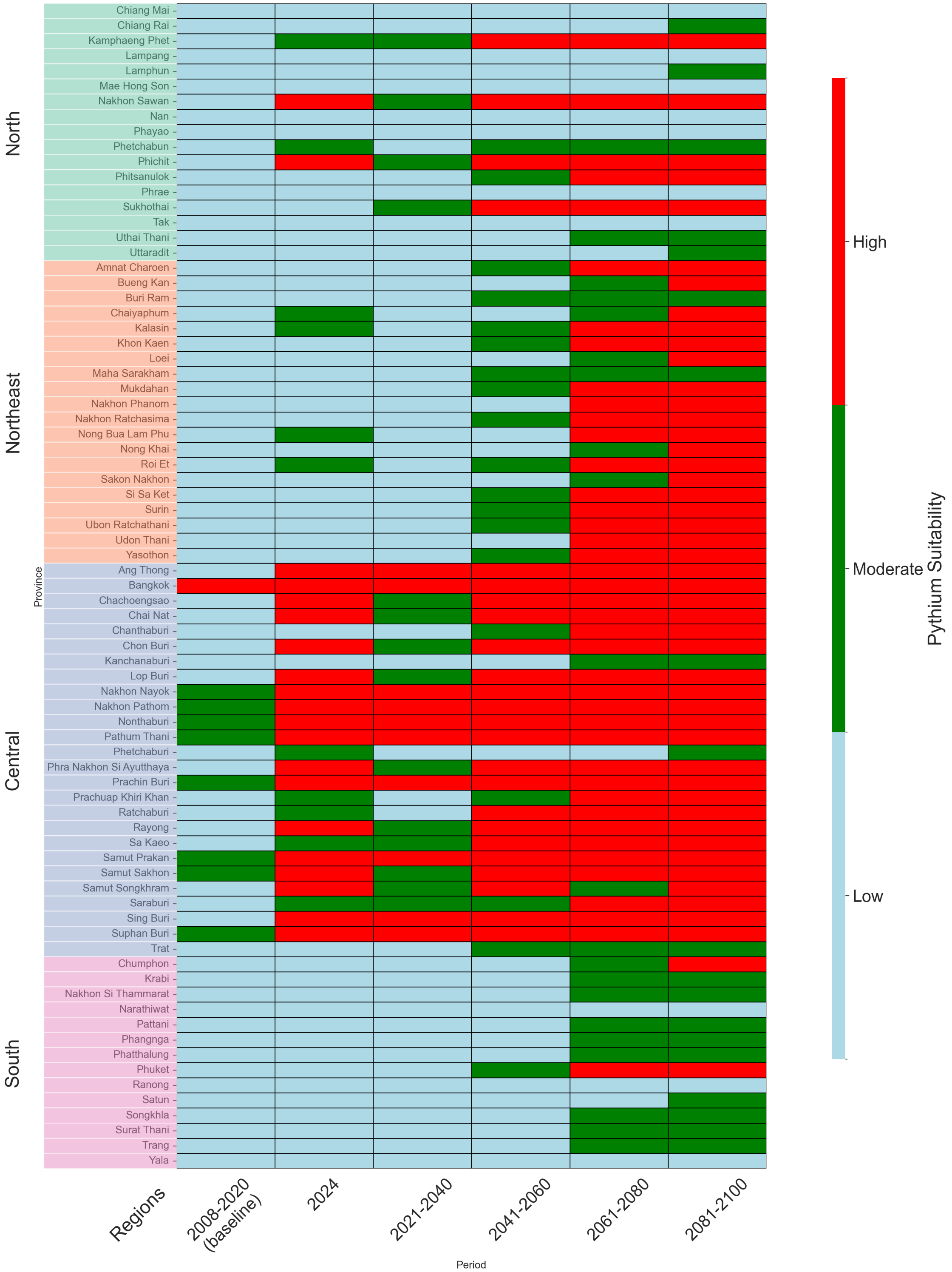

Figure S23: Provincial suitability distributions at 2008-2020 baseline, near-current projection (2024), and long-term projection for 2021–2040, 2041–2060, 2061–2080, and 2081–2100 under SSP3-7.0, showing threshold-based category shifts with provinces classified into high ($\geqslant$0.60), moderate (0.30–0.59), and low ($<$0.30) suitability.

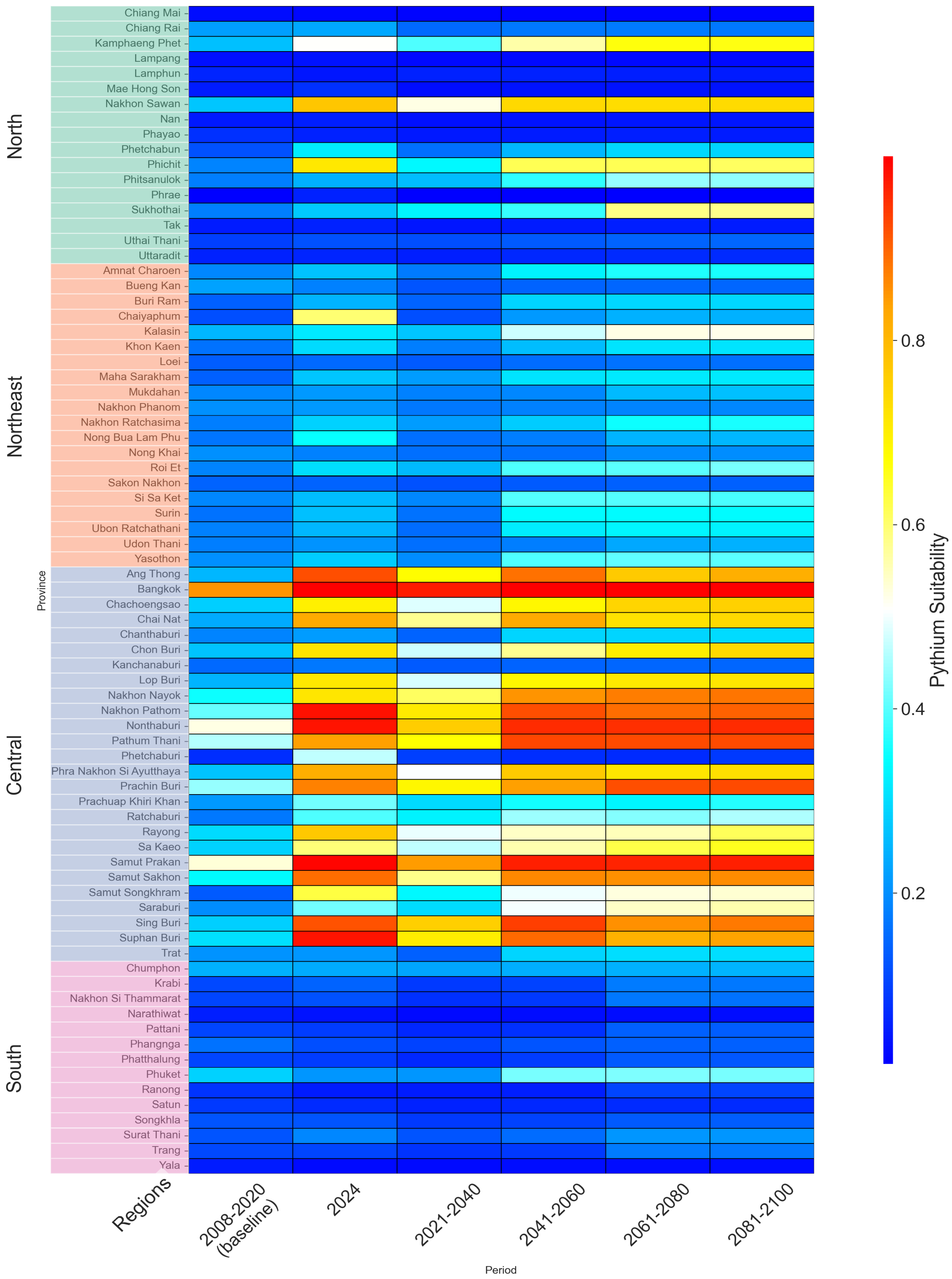

Figure S24: Provincial suitability distributions under baseline conditions, near-current projection (2024), and long-term projection for 2021–2040, 2041–2060, 2061–2080, and 2081–2100 under SSP1-2.6. Values represent continuous suitability probabilities (0–1) estimated using MaxEnt.

Under SSP1–2.6, the probability map (Fig. S24) shows relatively limited changes in suitability, with most provinces remaining in low or moderate categories throughout the century. In the *North* (e.g., Chiang Mai, Lampang, Phitsanulok, Sukhothai, Uttaradit), probabilities remain low through the baseline and early century, with gradual increases reaching moderate levels by 2041–2060, while many provinces (e.g., Chiang Rai, Mae Hong Son, Tak) remain persistently low. In contrast, in the *Northeast* (e.g., Khon Kaen, Nakhon Ratchasima, Udon Thani, Maha Sarakham, Roi Et), suitability increases modestly from low at baseline to moderate by 2041 to 2060, but most provinces stabilize at this level with little further change through 2100. The *Central region* (e.g., Bangkok, Chon Buri, Nonthaburi, Pathum Thani, Ayutthaya,

Samut Prakan) maintains moderate probabilities from baseline onward, with some provinces reaching higher levels by the late century. However, the increases are smaller and slower compared to other SSPs. Meanwhile, the *South* (e.g., Surat Thani, Songkhla, Phuket, Nakhon Si Thammarat) remains mainly in the low category across all periods, with only slight late-century increases in some coastal areas that do not cross into the moderate range.

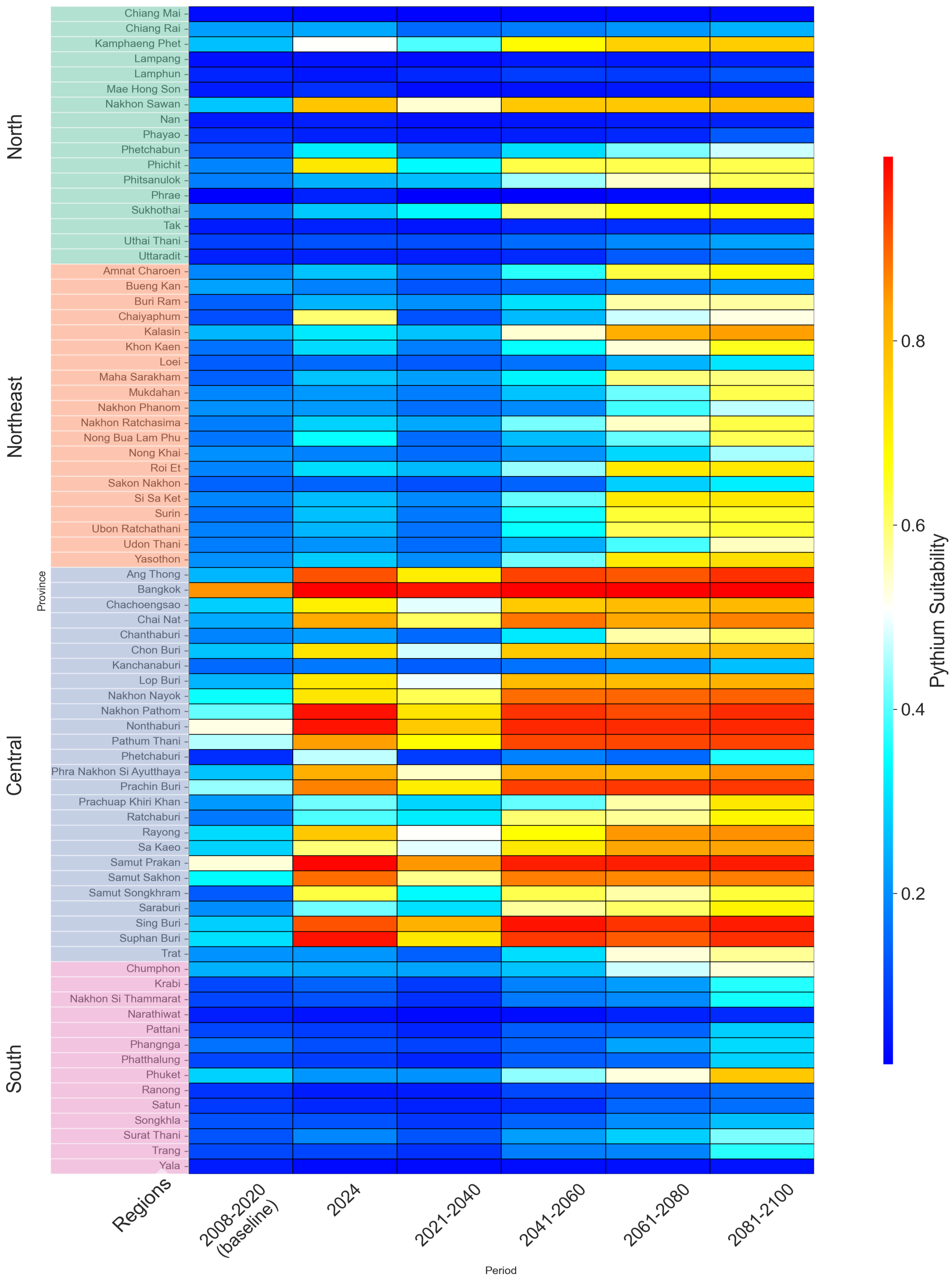

Figure S25: Provincial suitability distributions under baseline conditions, near-current projection (2024), and long-term projection for 2021–2040, 2041–2060, 2061–2080, and 2081–2100 under SSP2-4.5 (2021–2100). Values represent continuous suitability probabilities (0–1) estimated using MaxEnt.

Under SSP2–4.5, the probability map (Fig. S25) shows moderate but uneven changes between regions. In the *North* (e.g., Chiang Mai, Lampang, Phitsanulok, Sukhothai, Uttaradit), probabilities remain low at baseline and early century, with gradual increases towards moderate by 2041-2060, while many provinces (e.g., Chiang Rai, Mae Hong Son, Tak) remain at a low level until 2100. In the *Northeast* (e.g., Khon Kaen, Nakhon Ratchasima, Udon Thani, Maha Sarakham, Roi Et), suitability increases from low at baseline to moderate around 2041–2060, with some provinces maintaining this level and others showing smaller gains that level off later in the century. The *Central* region (e.g., Bangkok, Chon Buri, Nonthaburi, Pathum Thani, Ayutthaya, Samut Prakan) shows the largest increases, shifting from moderate at baseline to

high probabilities by 2081–2100. Neighboring provinces (e.g., Lop Buri, Ratchaburi, Kanchanaburi, Rayong) increase more slowly, some remaining moderate through the late century. In the *South* (e.g., Surat Thani, Songkhla, Phuket, Nakhon Si Thammarat), most provinces remain in the low category throughout all periods, although a few coastal provinces show modest upward trends after 2041–2060, without exceeding the moderate threshold.

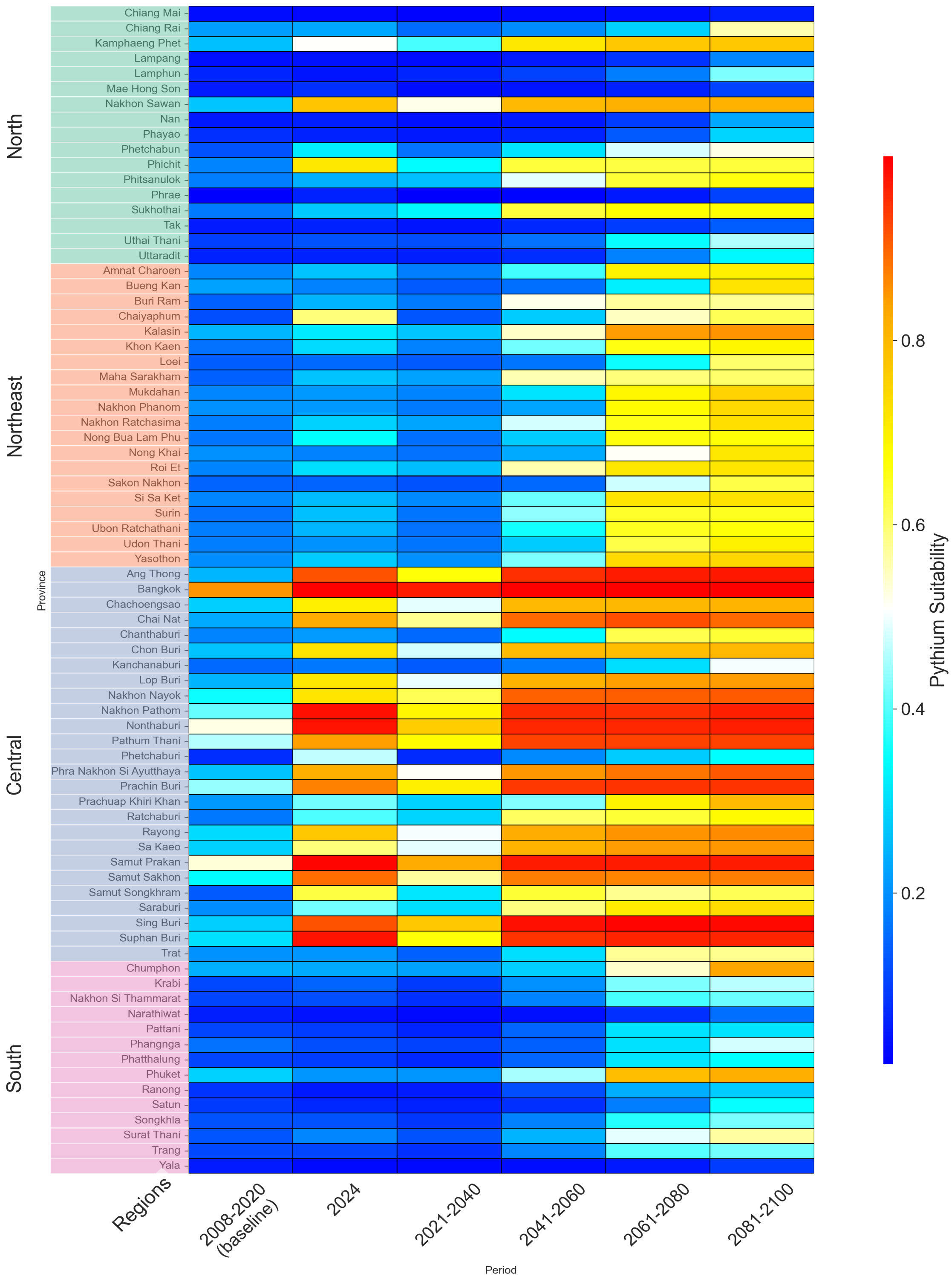

Figure S26: Provincial suitability distributions under baseline conditions, near-current projection (2024), and long-term projection for 2021–2040, 2041–2060, 2061–2080, and 2081–2100 under SSP3–7.0. Values represent continuous suitability probabilities (0–1) estimated using MaxEnt.

Under SSP3–7.0 (Fig. S26), the probability map shows moderate increases, but regional differences are subtler than under SSP5–8.5. In the *North* (e.g., Chiang Mai, Lampang, Phitsanulok, Sukhothai, Uttaradit), probabilities remain low through the baseline and early century, with gradual increases to moderate after 2041–2060, while many provinces (e.g., Chiang Rai, Mae Hong Son, Tak) remain persistently low. In the *Northeast* (e.g., Khon Kaen, Nakhon Ratchasima, Udon Thani, Maha Sarakham, Roi Et), suitability increases from low at baseline to moderate in the mid-century and then shows little further change, although increases are weaker than in SSP5–8.5. The *Central* region (e.g., Bangkok, Chon Buri, Nonthaburi, Pathum Thani, Ayutthaya, Samut Prakan) shows the highest probabilities, changing from moderate at the

beginning of the study to high in 2081 to 2100, with surrounding provinces (e.g. Lop Buri, Ratchaburi, Kanchanaburi, Rayong) increasing more gradually and some remaining moderate. In the *South* (e.g., Surat Thani, Songkhla, Phuket, Nakhon Si Thammarat), probabilities remain low in most provinces, although some coastal provinces show gradual upward trends after 2041–2060, indicating modest late-century increases.

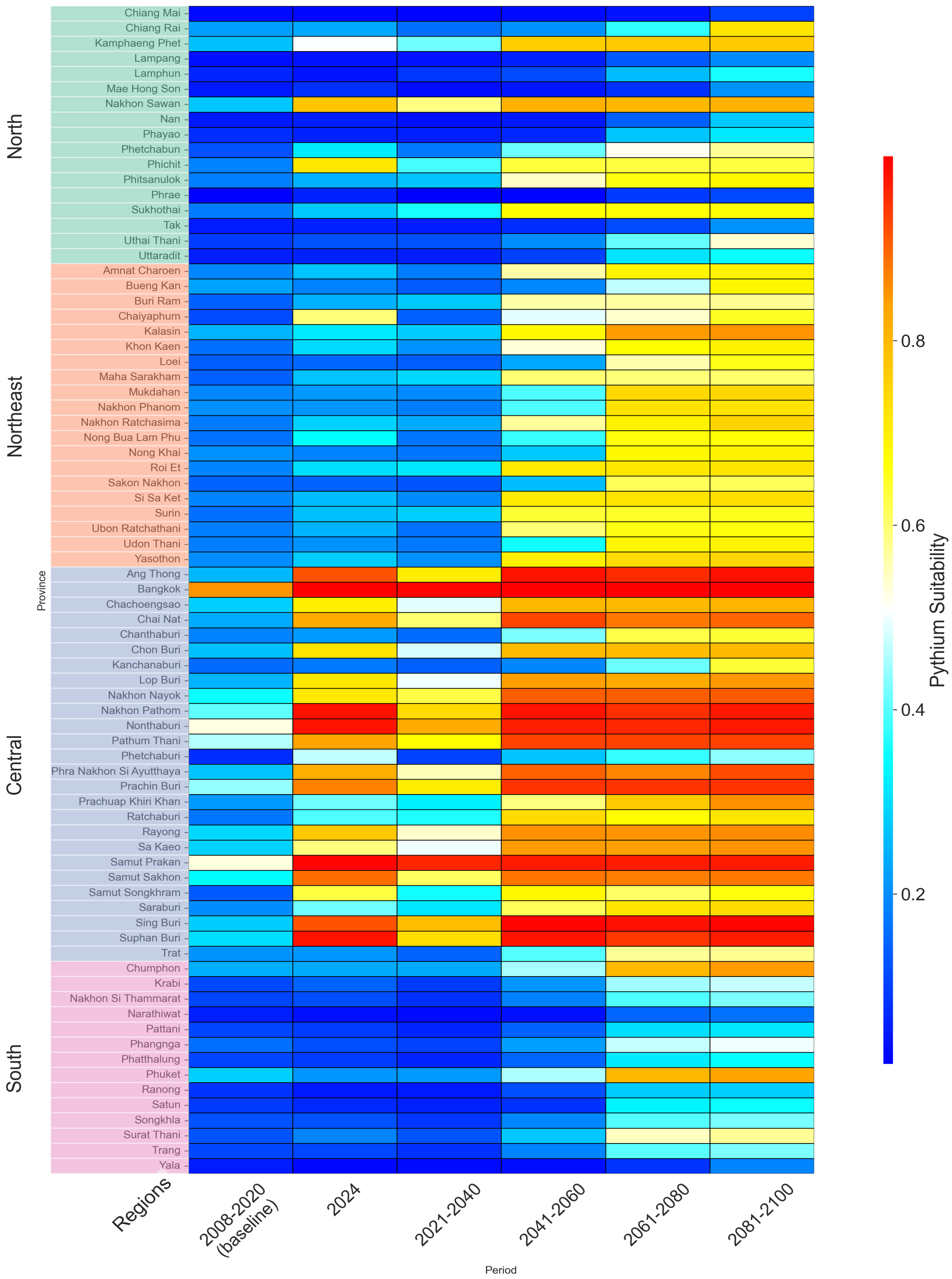

Figure S27: Provincial suitability distributions under baseline conditions, near-current projection (2024), and long-term projection for 2021–2040, 2041–2060, 2061–2080, and 2081–2100 under SSP5–8.5). Values represent continuous suitability probabilities (0–1) estimated using MaxEnt.

Under SSP5–8.5 (Fig. S27), the probability map shows more substantial and more uneven regional changes compared to all other SSP scenarios. In the *North* (e.g., Chiang Mai, Lampang, Phayao, Phichit, Sukhothai, Uttaradit), probabilities remain low at the beginning of the century, increase modestly to moderate by 2041-2020, and then stabilize, while most other northern provinces stay low throughout. In the *Northeast* (e.g., Khon Kaen, Nakhon Ratchasima, Udon Thani, Maha Sarakham, Roi Et), suitability increases from low at baseline to moderate by 2041-2060, although strength and timing vary, with some provinces stabilizing earlier and others later. The *Central* region (e.g., Bangkok, Chon Buri, Nakhon Pathom, Nonthaburi, Pathum Thani, Ayutthaya, Samut Prakan) shows the highest increases, with a large group that changes from

moderate at baseline to high by 2081–2100. Surrounding provinces (e.g., Lop Buri, Ratchaburi, Kanchanaburi) increase more gradually, while a few remain moderate through the late century. In the *South* (e.g., Surat Thani, Songkhla, Phuket, Nakhon Si Thammarat), most provinces remain low, although some coastal provinces show gradual upward trends after 2041-2060, indicating modest increases in the late century.

5 MESS values

Table S5: Area percentages of provinces with novel environments (2024)

**Province**		**Province**	
Amnat Charoen	0.00	Phatthalung	0.00
Ang Thong	100.00	Phayao	0.00
Bangkok	100.00	Phetchabun	28.97
Bueng Kan	0.00	Phetchaburi	68.46
Buri Ram	0.00	Phichit	98.60
Chachoengsao	80.80	Phitsanulok	14.73
Chai Nat	100.00	Phra Nakhon Si Ayutthaya	100.00
Chaiyaphum	40.33	Phrae	0.00
Chanthaburi	28.52	Phuket	30.43
Chiang Mai	0.00	Prachin Buri	63.11
Chiang Rai	0.00	Prachuap Khiri Khan	40.33
Chon Buri	85.65	Ranong	0.68
Chumphon	0.00	Ratchaburi	42.40
Kalasin	0.00	Rayong	79.89
Kamphaeng Phet	50.12	Roi Et	0.00
Kanchanaburi	22.44	Sa Kaeo	62.88
Khon Kaen	1.35	Sakon Nakhon	0.00
Krabi	72.37	Samut Prakan	100.00
Lampang	0.00	Samut Sakhon	100.00
Lamphun	0.00	Samut Songkhram	100.00
Loei	0.00	Saraburi	56.55
Lop Buri	71.07	Satun	23.19
Mae Hong Son	1.27	Si Sa Ket	0.23
Maha Sarakham	0.00	Sing Buri	100.00
Mukdahan	0.00	Songkhla	5.11
Nakhon Nayok	64.15	Sukhothai	14.72
Nakhon Pathom	100.00	Suphan Buri	80.75
Nakhon Phanom	0.00	Surat Thani	36.83
Nakhon Ratchasima	5.36	Surin	0.00
Nakhon Sawan	83.98	Tak	0.24
Nakhon Si Thammarat	55.32	Trang	44.70
Nan	0.00	Trat	48.92
Narathiwat	0.48	Ubon Ratchathani	0.67
Nong Bua Lam Phu	0.00	Udon Thani	0.00
Nong Khai	0.00	Uthai Thani	32.61
Nonthaburi	100.00	Uttaradit	0.26
Pathum Thani	100.00	Yala	10.43
Pattani	20.00	Yasothon	0.00
Phangnga	15.30		

Table S6: Area percentages of provinces with novel environments (SSP1–2.6)

Province	2021–2040 (%)	2041–2060 (%)	2061–2080 (%)	2081–2100 (%)
Amnat Charoen	0.00	63.52	89.31	91.19
Ang Thong	100.00	100.00	100.00	100.00
Bangkok	100.00	100.00	100.00	100.00
Bueng Kan	0.00	0.00	0.00	0.00
Buri Ram	21.07	89.67	91.94	92.56
Chachoengsao	79.12	88.76	93.98	94.78
Chai Nat	100.00	100.00	100.00	100.00
Chaiyaphum	0.65	46.67	61.95	62.11
Chanthaburi	2.02	64.31	70.03	71.72
Chiang Mai	0.00	0.00	0.00	0.00
Chiang Rai	0.00	0.00	0.00	0.00
Chon Buri	83.25	95.69	96.65	97.13
Chumphon	0.00	2.10	21.33	23.08
Kalasin	0.00	72.32	89.29	90.18
Kamphaeng Phet	71.84	81.15	82.82	82.82
Kanchanaburi	27.24	36.22	43.06	43.38
Khon Kaen	2.69	66.92	86.35	87.31
Krabi	4.85	49.78	74.89	75.33
Lampang	0.00	3.59	6.69	6.36
Lamphun	0.00	0.00	0.00	0.00
Loei	0.00	0.00	0.77	0.77
Lop Buri	83.65	95.91	97.80	97.80
Mae Hong Son	0.00	0.00	0.16	0.16
Maha Sarakham	22.06	100.00	100.00	100.00
Mukdahan	0.00	0.00	35.82	39.80
Nakhon Nayok	69.81	72.64	72.64	74.53
Nakhon Pathom	100.00	100.00	100.00	100.00
Nakhon Phanom	0.00	0.00	3.62	10.51
Nakhon Ratchasima	26.02	68.22	73.68	73.88
Nakhon Sawan	92.21	94.81	95.02	95.02
Nakhon Si Tham-marat	1.91	45.53	64.04	65.53
Nan	0.00	0.00	0.00	0.00
Narathiwat	0.00	0.48	11.54	12.02
Nong Bua Lam Phu	0.00	3.03	61.62	63.64
Nong Khai	0.00	0.00	14.47	20.13
Nonthaburi	100.00	100.00	100.00	100.00
Pathum Thani	100.00	100.00	100.00	100.00
Pattani	0.00	42.22	78.89	78.89
Phangnga	0.00	32.79	54.64	55.19
Phichit	100.00	100.00	100.00	100.00
Phitsanulok	39.69	51.08	61.10	61.30
Phra Nakhon Si Ayutthaya	100.00	100.00	100.00	100.00
Phrae	0.00	0.62	16.46	15.53
Phuket	13.04	73.91	82.61	82.61
Prachin Buri	68.44	77.05	78.28	78.69
Prachuap Khiri Khan	16.07	38.03	48.20	49.51
Ranong	0.00	0.00	8.16	10.20
Ratchaburi	55.20	65.20	69.20	70.00
Rayong	68.16	91.62	93.85	94.97
Roi Et	20.11	96.03	98.68	98.94
Sa Kaeo	64.72	86.50	88.34	88.65
Sakon Nakhon	0.00	0.00	0.22	3.24
Samut Prakan	100.00	100.00	100.00	100.00
Samut Sakhon	100.00	100.00	100.00	100.00
Samut Songkhram	100.00	100.00	100.00	100.00
Saraburi	60.12	72.62	80.36	80.36
Satun	0.00	4.35	44.20	43.48
Si Sa Ket	0.00	81.97	88.52	88.76
Sing Buri	100.00	100.00	100.00	100.00
Songkhla	0.30	29.13	50.15	51.95
Sukhothai	60.43	73.93	83.13	83.13
Suphan Buri	84.91	89.06	90.57	90.94
Surat Thani	1.94	29.40	47.17	47.98
Surin	13.27	90.52	93.84	93.84
Tak	5.32	14.30	15.84	15.84
Trang	0.46	41.94	70.51	70.97
Trat	6.47	66.19	71.94	74.10
Ubon Ratchathani	0.00	79.25	89.42	89.83
Udon Thani	0.00	8.72	43.04	49.17
Uthai Thani	43.48	52.17	53.73	53.73
Uttaradit	19.64	32.65	41.07	41.07
Yala	0.00	2.37	7.11	7.11
Yasothon	0.00	82.72	95.81	97.38

Table S7: Area percentages of provinces with novel environments (SSP2–4.5)

**Province**	**2021–2040** (%)	**2041–2060** (%)	**2061–2080** (%)	**2081–2100** (%)	**Province**	**2021–2040** (%)	**2041–2060** (%)	**2061–2080** (%)	**2081–2100** (%)
Amnat Charoen	0.00	89.31	100.00	100.00	Phatthalung	0.00	62.26	80.50	83.02
Ang Thong	100.00	100.00	100.00	100.00	Phayao	0.00	0.00	1.97	34.21
Bangkok	100.00	100.00	100.00	100.00	Phetchabun	36.59	60.60	69.21	75.00
Bueng Kan	0.00	0.00	0.00	24.10	Phetchaburi	38.26	52.01	58.72	64.43
Buri Ram	31.61	92.56	97.73	98.97	Phichit	100.00	100.00	100.00	100.00
Chachoengsao	81.12	95.18	98.80	100.00	Phitsanulok	39.49	61.49	71.51	78.19
Chai Nat	100.00	100.00	100.00	100.00	Phra Nakhon Si Ayutthaya	100.00	100.00	100.00	100.00
Chaiyaphum	2.11	62.93	73.17	78.54	Phrae	0.00	16.77	47.83	67.08
Chanthaburi	3.03	73.06	83.50	87.88	Phuket	13.04	82.61	86.96	95.65
Chiang Mai	0.00	0.00	7.08	13.52	Prachin Buri	69.67	78.69	83.20	85.66
Chiang Rai	0.00	0.00	1.20	32.53	Prachuap Khiri Khan	15.41	50.49	69.51	77.05
Chon Buri	85.17	97.61	99.52	99.52	Ranong	0.00	12.24	48.30	61.22
Chumphon	0.00	24.83	65.38	78.67	Ratchaburi	54.80	70.80	78.80	83.60
Kalasin	0.00	89.88	97.32	99.40	Rayong	71.51	95.53	97.77	98.88
Kamphaeng Phet	71.12	82.82	85.20	87.11	Roi Et	21.43	98.68	99.21	100.00
Kanchanaburi	27.03	43.59	57.59	64.96	Sa Kaeo	67.79	88.65	94.79	96.93
Khon Kaen	3.46	88.08	94.81	97.88	Sakon Nakhon	0.00	0.43	76.89	96.54
Krabi	3.96	77.53	94.71	98.24	Samut Prakan	100.00	100.00	100.00	100.00
Lampang	0.00	7.18	30.02	49.10	Samut Sakhon	100.00	100.00	100.00	100.00
Lamphun	0.00	0.00	18.10	35.29	Samut Songkhram	100.00	100.00	100.00	100.00
Loei	0.00	0.77	40.96	59.81	Saraburi	60.71	80.36	91.07	97.62
Lop Buri	84.59	98.74	99.06	100.00	Satun	0.00	47.10	71.01	80.43
Mae Hong Son	0.00	0.16	7.94	16.98	Si Sa Ket	0.00	88.76	93.44	95.78
Maha Sarakham	23.53	100.00	100.00	100.00	Sing Buri	100.00	100.00	100.00	100.00
Mukdahan	0.00	36.82	82.59	94.53	Songkhla	0.30	51.95	80.78	89.19
Nakhon Nayok	69.81	74.53	79.25	80.19	Sukhothai	59.82	83.13	94.17	96.93
Nakhon Pathom	100.00	100.00	100.00	100.00	Suphan Buri	84.15	90.94	95.85	98.11
Nakhon Phanom	0.00	3.99	76.81	96.01	Surat Thani	1.94	49.76	71.41	77.87
Nakhon Ratchasima	28.70	74.98	85.00	91.66	Surin	17.77	93.84	98.34	99.76
Nakhon Sawan	91.99	95.02	97.19	97.19	Tak	4.73	15.84	25.41	31.91
Nakhon Si Thammarat	1.91	66.81	80.21	83.83	Trang	0.46	72.35	85.71	90.78
Nan	0.00	0.00	10.91	27.52	Trat	5.04	75.54	92.09	94.96
Narathiwat	0.00	14.42	58.65	70.19	Ubon Ratchathani	0.27	89.69	94.38	96.79
Nong Bua Lam Phu	0.00	63.64	95.45	100.00	Udon Thani	0.00	45.45	92.95	97.22
Nong Khai	0.00	16.98	57.23	89.31	Uthai Thani	43.17	54.66	64.91	70.19
Nonthaburi	100.00	100.00	100.00	100.00	Uttaradit	19.13	41.33	58.16	71.17
Pathum Thani	100.00	100.00	100.00	100.00	Yala	0.00	7.58	31.75	41.71
Pattani	0.00	78.89	95.56	97.78	Yasothon	0.00	96.34	100.00	100.00
Phangnga	0.00	56.28	72.68	79.78					

Table S8: Area percentages of provinces with novel environments (SSP3–7.0)

**Province**	**2021–2040** (%)	**2041–2060** (%)	**2061–2080** (%)	**2081–2100** (%)	**Province**	**2021–2040** (%)	**2041–2060** (%)	**2061–2080** (%)	**2081–2100** (%)
Amnat Charoen	0.00	96.86	100.00	100.00	Phatthalung	0.00	69.81	86.79	93.71
Ang Thong	100.00	100.00	100.00	100.00	Phayao	0.00	0.00	48.68	82.57
Bangkok	100.00	100.00	100.00	100.00	Phetchabun	35.60	62.42	76.16	88.91
Bueng Kan	0.00	0.00	63.59	100.00	Phetchaburi	34.23	52.35	66.78	78.19
Buri Ram	26.65	93.80	99.38	100.00	Phichit	100.00	100.00	100.00	100.00
Chachoengsao	79.12	97.59	100.00	100.00	Phitsanulok	39.69	64.44	79.96	92.53
Chai Nat	100.00	100.00	100.00	100.00	Phra Nakhon Si Ayutthaya	100.00	100.00	100.00	100.00
Chaiyaphum	0.65	65.37	80.33	88.46	Phrae	0.00	23.91	72.67	92.55
Chanthaburi	3.03	76.43	89.90	96.30	Phuket	13.04	82.61	100.00	100.00
Chiang Mai	0.00	0.09	16.38	39.93	Prachin Buri	68.85	79.10	86.89	93.85
Chiang Rai	0.00	0.00	43.72	70.91	Prachuap Khiri Khan	14.75	53.77	78.69	90.82
Chon Buri	83.73	98.09	100.00	100.00	Ranong	0.00	18.37	68.71	89.80
Chumphon	0.00	33.92	82.87	97.20	Ratchaburi	53.20	71.60	84.40	94.40
Kalasin	0.00	92.86	99.40	100.00	Rayong	68.72	96.65	98.88	100.00
Kamphaeng Phet	70.88	83.29	87.83	91.89	Roi Et	21.16	98.94	100.00	100.00
Kanchanaburi	26.07	45.83	67.63	82.59	Sa Kaeo	66.56	90.80	97.85	100.00
Khon Kaen	3.08	89.62	98.46	100.00	Sakon Nakhon	0.00	12.74	98.27	100.00
Krabi	5.29	86.78	98.68	99.12	Samut Prakan	100.00	100.00	100.00	100.00
Lampang	0.00	11.09	59.38	85.97	Samut Sakhon	100.00	100.00	100.00	100.00
Lamphun	0.00	0.00	45.70	86.88	Samut Songkhram	100.00	100.00	100.00	100.00
Loei	0.00	3.08	66.15	85.77	Saraburi	58.33	82.14	98.21	99.40
Lop Buri	83.02	98.74	100.00	100.00	Satun	0.00	54.35	86.23	94.20
Mae Hong Son	0.00	0.32	22.38	49.21	Si Sa Ket	0.00	89.93	96.49	100.00
Maha Sarakham	23.90	100.00	100.00	100.00	Sing Buri	100.00	100.00	100.00	100.00
Mukdahan	0.00	50.25	99.00	100.00	Songkhla	0.30	65.77	92.79	97.90
Nakhon Nayok	69.81	76.42	82.08	87.74	Sukhothai	60.12	85.28	96.93	99.39
Nakhon Pathom	100.00	100.00	100.00	100.00	Suphan Buri	84.15	91.32	98.49	100.00
Nakhon Phanom	0.00	21.74	97.46	100.00	Surat Thani	2.10	58.32	81.26	93.54
Nakhon Ratchasima	26.51	76.56	93.05	98.71	Surin	16.59	95.97	100.00	100.00
Nakhon Sawan	91.77	95.67	97.62	98.92	Tak	4.85	17.14	34.87	58.51
Nakhon Si Thammarat	2.77	74.68	88.30	92.77	Trang	0.46	77.42	93.09	95.39
Nan	0.00	0.17	35.74	70.30	Trat	5.04	78.42	95.68	99.28
Narathiwat	0.00	36.06	77.88	90.87	Ubon Ratchathani	0.67	91.30	97.46	100.00
Nong Bua Lam Phu	0.00	75.25	100.00	100.00	Udon Thani	0.00	61.97	98.33	100.00
Nong Khai	0.00	32.70	98.74	100.00	Uthai Thani	41.93	56.21	73.60	87.58
Nonthaburi	100.00	100.00	100.00	100.00	Uttaradit	19.64	46.68	76.53	95.92
Pathum Thani	100.00	100.00	100.00	100.00	Yala	0.00	18.01	51.18	75.83
Pattani	0.00	91.11	97.78	100.00	Yasothon	0.00	98.95	100.00	100.00
Phangnga	0.00	61.20	85.25	95.08					

Table S9: Area percentages of provinces with novel environments (SSP5–8.5)

**Province**	**2021–2040** (%)	**2041–2060** (%)	**2061–2080** (%)	**2081–2100** (%)	**Province**	**2021–2040** (%)	**2041–2060** (%)	**2061–2080** (%)	**2081–2100** (%)
Amnat Charoen	0.00	100.00	100.00	100.00	Phatthalung	0.00	76.10	91.19	98.74
Ang Thong	100.00	100.00	100.00	100.00	Phayao	0.00	0.33	73.03	96.71
Bangkok	100.00	100.00	100.00	100.00	Phetchabun	41.39	67.55	84.60	99.17
Bueng Kan	0.00	0.00	100.00	100.00	Phetchaburi	42.62	57.05	74.16	95.30
Buri Ram	65.70	97.31	100.00	100.00	Phichit	100.00	100.00	100.00	100.00
Chachoengsao	83.53	98.39	100.00	100.00	Phitsanulok	41.06	68.76	87.62	98.82
Chai Nat	100.00	100.00	100.00	100.00	Phra Nakhon Si Ayutthaya	100.00	100.00	100.00	100.00
Chaiyaphum	9.92	71.54	87.15	99.67	Phrae	0.00	38.51	88.51	99.38
Chanthaburi	16.16	82.83	94.61	99.66	Phuket	26.09	86.96	100.00	100.00
Chiang Mai	0.00	3.04	31.83	69.55	Prachin Buri	70.49	81.97	90.98	100.00
Chiang Rai	0.00	0.00	62.48	91.22	Prachuap Khiri Khan	21.31	65.57	85.57	98.03
Chon Buri	89.95	99.52	100.00	100.00	Ranong	0.00	40.14	81.63	98.64
Chumphon	0.00	56.29	92.66	99.65	Ratchaburi	56.80	76.80	90.80	100.00
Kalasin	3.57	96.43	100.00	100.00	Rayong	81.01	97.77	100.00	100.00
Kamphaeng Phet	75.18	84.25	91.17	96.90	Roi Et	38.62	99.21	100.00	100.00
Kanchanaburi	29.38	52.35	77.56	96.37	Sa Kaeo	74.23	93.25	99.39	100.00
Khon Kaen	13.46	94.23	100.00	100.00	Sakon Nakhon	0.00	64.58	100.00	100.00
Krabi	11.89	92.07	99.12	99.56	Samut Prakan	100.00	100.00	100.00	100.00
Lampang	0.00	22.35	78.47	98.53	Samut Sakhon	100.00	100.00	100.00	100.00
Lamphun	0.00	8.60	74.66	99.55	Samut Songkhram	100.00	100.00	100.00	100.00
Loei	0.00	30.96	80.38	96.73	Saraburi	62.50	88.10	99.40	100.00
Lop Buri	88.05	99.06	100.00	100.00	Satun	0.00	62.32	90.58	100.00
Mae Hong Son	0.00	2.70	39.68	78.57	Si Sa Ket	1.87	92.27	100.00	100.00
Maha Sarakham	65.07	100.00	100.00	100.00	Sing Buri	100.00	100.00	100.00	100.00
Mukdahan	0.00	75.62	100.00	100.00	Songkhla	0.60	76.58	97.00	99.40
Nakhon Nayok	71.70	78.30	84.91	98.11	Sukhothai	63.50	91.41	98.77	100.00
Nakhon Pathom	100.00	100.00	100.00	100.00	Suphan Buri	85.66	94.34	99.25	100.00
Nakhon Phanom	0.00	64.13	100.00	100.00	Surat Thani	3.07	67.21	89.01	98.87
Nakhon Ratchasima	40.91	83.02	97.12	100.00	Surin	48.82	97.63	100.00	100.00
Nakhon Sawan	92.64	96.75	98.27	99.57	Tak	8.63	22.10	49.53	83.69
Nakhon Si Thammarat	6.60	78.30	91.28	97.23	Trang	0.46	83.87	94.47	97.70
Nan	0.00	4.03	59.73	91.28	Trat	13.67	88.49	98.56	99.28
Narathiwat	0.00	53.85	87.98	96.63	Ubon Ratchathani	2.41	93.84	99.73	100.00
Nong Bua Lam Phu	0.00	93.94	100.00	100.00	Udon Thani	0.00	85.90	99.81	100.00
Nong Khai	0.00	46.54	100.00	100.00	Uthai Thani	45.03	62.73	84.78	96.58
Nonthaburi	100.00	100.00	100.00	100.00	Uttaradit	22.70	53.83	90.82	99.49
Pathum Thani	100.00	100.00	100.00	100.00	Yala	0.00	26.07	69.67	91.47
Pattani	0.00	95.56	98.89	100.00	Yasothon	0.00	99.48	100.00	100.00
Phangnga	0.55	71.58	91.26	97.81					

Figure S28: Lambda values from MaxEnt model calibration, showing fitted feature weights and regularization parameters for fold 0.

```
(landcover=10.0), 0.5516492635320103, 0.0, 1.0
(landcover=20.0), 0.8785729951064344, 0.0, 1.0
(landcover=30.0), 0.24286707689109646, 0.0, 1.0
(landcover=100.0), 0.2560775172512121, 0.0, 1.0
(landcover=130.0), 2.180186952922501, 0.0, 1.0
(landcover=190.0), 0.8193596409465485, 0.0, 1.0
(landuse=0.0), 0.0, 0.0, 1.0
(landuse=1.0), -1.5661642026947211, 0.0, 1.0
(landuse=2.0), 0.33938757698627264, 0.0, 1.0
(landuse=6.0), -0.6585007368372057, 0.0, 1.0
(landuse=7.0), 0.4958260718535063, 0.0, 1.0
(landuse=9.0), 0.6747786001031638, 0.0, 1.0
ndvi, 0.0, -0.11309412, 0.8416913
ppt, 0.0, 62.794445, 216.23611
soil, 3.2983506052768345, 22.041666, 440.89166
tavg, 1.7967892313746663, 20.402779, 29.20139
ndvi^2, -1.5126461340287698, 1.0707767968710759E-5, 0.7084442444956901
‘ppt, 0.229305972749574, 62.794445, 74.05694700000001
’tavg, 1.0738064496428006, 28.395138, 29.20139
‘soil, 1.7575601108866417, 22.041666, 66.408332
’tavg, 0.8885007354877341, 28.9465275, 29.20139
‘ppt, 0.17261093335820102, 62.794445, 85.52639
‘ppt, 0.09308213018391572, 62.794445, 85.684722
‘ppt, 0.12443864085023223, 62.794445, 85.57638700000001
‘ppt, 0.14087201433978053, 62.794445, 85.51806
‘ppt, 0.13880775512933716, 62.794445, 85.5375
‘ppt, 0.11586334617934907, 62.794445, 85.565278
‘ppt, 0.0687135164028223, 62.794445, 85.67917
‘ppt, 0.027505938682260606, 62.794445, 85.57916700000001
‘ppt, 0.05215028050524708, 62.794445, 85.59028
‘ppt, 0.394004395038107, 62.794445, 85.620833
‘ppt, 0.23111091338747797, 62.794445, 85.63194200000001
’tavg, 0.10924850294643561, 28.945137, 29.20139
‘ppt, 0.5757975461789675, 62.794445, 85.62361
‘ppt, 0.015047177070559862, 62.794445, 85.62777700000001
’tavg, 0.15931548754296676, 28.428470500000003, 29.20139
‘ppt, 0.16126616113233422, 62.794445, 85.61805799999999
‘ppt, 0.05175112380116846, 62.794445, 85.61528
‘ppt, 0.12523620905613628, 62.794445, 73.65972
‘ppt, 0.15677617410105316, 62.794445, 73.665275
‘ppt, 0.18892507308618148, 62.794445, 85.59305699999999
‘ppt, 0.014353951146660934, 62.794445, 73.67083299999999
‘ppt, 0.01539060644560292, 62.794445, 73.675003
‘ppt, 0.01210196359699581, 62.794445, 73.67917
‘ppt, 0.009437348126814704, 62.794445, 73.684722
’tavg, 0.018873090688751357, 28.429859, 29.20139
‘ppt, 0.010592377103012683, 62.794445, 73.6875
‘ppt, 0.016624236810254142, 62.794445, 73.69722
linearPredictorNormalizer, 6.229877304037299
densityNormalizer, 616.097577585467
numBackgroundPoints, 21117
entropy, 9.188044753919351
```

Figure S29: Lambda values from MaxEnt model calibration, showing fitted feature weights and regularization parameters for fold 1.

```
(landcover=10.0), 0.19282202850543337, 0.0, 1.0
(landcover=11.0), 0.17990839714449444, 0.0, 1.0
(landcover=20.0), 0.7811251951262904, 0.0, 1.0
(landcover=30.0), 0.08183581281168371, 0.0, 1.0
(landcover=100.0), 0.0999118644981574, 0.0, 1.0
(landcover=130.0), 1.9506680498271842, 0.0, 1.0
(landcover=190.0), 1.434468817998982, 0.0, 1.0
(landuse=0.0), 0.0, 0.0, 1.0
(landuse=1.0), -1.8366813335034116, 0.0, 1.0
(landuse=2.0), 0.07124391463959145, 0.0, 1.0
(landuse=6.0), -0.7574509981305347, 0.0, 1.0
(landuse=7.0), 0.1907259087680808, 0.0, 1.0
(landuse=9.0), 0.5562468359304013, 0.0, 1.0
(landuse=10.0), 0.024247231194412626, 0.0, 1.0
ndvi, 0.0, -0.11309412, 0.8416913
ppt, 0.0, 62.794445, 216.23611
soil, 3.1209013275482027, 22.041666, 440.89166
tavg, 1.5129202454490296, 20.402779, 29.20139
ndvi^2, -2.0094620264730643, 1.0707767968710759E-5, 0.7084442444956901
ppt^2, -0.4583346944418579, 3943.142322858025, 46758.0552679321
‘ppt, 0.8557096370833113, 62.794445, 73.65972
’tavg, 0.4693101505242554, 28.407638499999997, 29.20139
‘soil, 1.0402456552213284, 22.041666, 65.82778
‘ppt, 0.2665381427942572, 62.794445, 85.9875025
’tavg, 0.02140487493009552, 28.94236, 29.20139
’tavg, 0.2675603718167717, 28.9381945, 29.20139
‘ppt, 0.3556731484126395, 62.794445, 86.5152775
’tavg, 0.32468290201734107, 28.9340265, 29.20139
‘ppt, 0.046890901985607816, 62.794445, 86.79305199999999
‘ppt, 0.10362049931763645, 62.794445, 86.655555
’tavg, 0.19134587817259488, 28.932638, 29.20139
‘ppt, 0.13165127394967316, 62.794445, 86.76110750000001
‘ppt, 0.11049947131074472, 62.794445, 86.57083
‘ppt, 0.0808387728244435, 62.794445, 86.565278
‘ppt, 0.10874716670844803, 62.794445, 86.4902805
’tavg, 0.07882012788046461, 28.421528000000002, 29.20139
‘ppt, 0.12006615746388595, 62.794445, 86.5124975
‘ppt, 0.05009493603703955, 62.794445, 86.4847225
‘ppt, 0.07905090859937845, 62.794445, 73.1125
‘ppt, 0.051439655118070894, 62.794445, 86.51806
‘ppt, 0.04984864671164457, 62.794445, 86.469445
‘ppt, 0.06302627607595523, 62.794445, 86.4625
‘ppt, 0.062419617012134596, 62.794445, 86.43888799999999
‘ppt, 0.12213865776295031, 62.794445, 86.41805199999999
‘ppt, 0.12092239342457609, 62.794445, 86.40555499999999
‘ppt, 0.11978735280786411, 62.794445, 86.3874975
‘ppt, 0.0899985654704726, 62.794445, 73.13472
‘ppt, 0.05997697954895736, 62.794445, 86.379167
‘ppt, 0.059389318118270945, 62.794445, 86.37361
‘ppt, 0.0618723896734128, 62.794445, 86.370833
‘ppt, 0.06686075823872503, 62.794445, 86.163885
‘ppt, 0.046254840040292726, 62.794445, 73.18194700000001
‘ppt, 0.027659175524930413, 62.794445, 86.205555
‘ppt, 0.027377084175941507, 62.794445, 86.19861
‘ppt, 0.05381496562764415, 62.794445, 86.184722
‘ppt, 0.026934361256339894, 62.794445, 86.17083299999999
‘ppt, 0.03969409564688087, 62.794445, 73.20139
‘ppt, 0.03168846704525097, 62.794445, 86.05417
’tavg, 0.04134541123159004, 28.928470500000003, 29.20139
```

Figure S30: Lambda values from MaxEnt model calibration, showing fitted feature weights and regularization parameters for fold 2.

```
(landcover=10.0), 0.16490399567151345, 0.0, 1.0
(landcover=11.0), 0.16135471187535694, 0.0, 1.0
(landcover=20.0), 0.9813094209983253, 0.0, 1.0
(landcover=30.0), 0.176275572840984, 0.0, 1.0
(landcover=100.0), 0.06578846790216054, 0.0, 1.0
(landcover=190.0), 1.2752907671251574, 0.0, 1.0
(landuse=0.0), 0.0, 0.0, 1.0
(landuse=1.0), -1.4619389519259034, 0.0, 1.0
(landuse=2.0), 0.8026416023104622, 0.0, 1.0
(landuse=7.0), 0.9696871232795087, 0.0, 1.0
(landuse=9.0), 1.0604051503975642, 0.0, 1.0
ndvi, 0.0, -0.11309412, 0.8416913
ppt, 0.0, 62.794445, 216.23611
soil, 3.7076748660688454, 22.041666, 440.89166
tavg, 1.0827020805940362, 20.402779, 29.20139
ndvi^2, -1.7593771808369676, 1.0707767968710759E-5, 0.7084442444956901
ppt^2, -0.05359486627975257, 3943.142322858025, 46758.0552679321
‘ppt, 1.4596279795038225, 62.794445, 73.584723
’tavg, 0.60393059098485, 28.415971499999998, 29.20139
’tavg, 0.17492154670538224, 28.9381945, 29.20139
‘soil, 1.1981177241838503, 22.041666, 65.929165
’tavg, 0.1846067233312734, 28.935416, 29.20139
’tavg, 0.34376219117330786, 28.932638, 29.20139
‘ppt, 0.20306341841743694, 62.794445, 86.655555
‘ppt, 0.2604392685124369, 62.794445, 86.729163
‘ppt, 0.12825534830846014, 62.794445, 86.683335
‘ppt, 0.3057264520438568, 62.794445, 86.61805799999999
‘ppt, 0.03695594496653008, 62.794445, 86.80139
’tavg, 0.3335593283307052, 28.929859999999998, 29.20139
‘ppt, 0.16895867590880284, 62.794445, 86.75694200000001
‘ppt, 0.18470138605865033, 62.794445, 86.75277700000001
‘ppt, 0.085021272247119, 62.794445, 86.73194
‘ppt, 0.07283907798743465, 62.794445, 73.1875
‘ppt, 0.08184976228954795, 62.794445, 73.20139
‘ppt, 0.04641904682302602, 62.794445, 86.68888799999999
‘ppt, 0.020605947444318484, 62.794445, 86.65972
‘ppt, 0.022921919478947436, 62.794445, 73.226388
‘ppt, 0.03573614958387685, 62.794445, 73.23611
’tavg, 0.06959095543829133, 28.411804, 29.20139
‘ppt, 0.020299038798902436, 62.794445, 86.67917
‘ppt, 0.0414923286922127, 62.794445, 86.67361299999999
‘ppt, 0.023844486076280685, 62.794445, 86.67083299999999
‘ppt, 0.022520311550812605, 62.794445, 86.66805199999999
‘ppt, 0.021780480725240057, 62.794445, 86.6625
‘ppt, 0.22554202118478253, 62.794445, 86.650002
‘ppt, 0.1087859554913192, 62.794445, 86.644447
‘ppt, 0.021277019679888008, 62.794445, 86.63889
‘ppt, 0.03193709198641275, 62.794445, 73.24305799999999
‘ppt, 0.01565216341514486, 62.794445, 86.633332
‘ppt, 0.01560196296617856, 62.794445, 86.629167
‘ppt, 0.06052867650579081, 62.794445, 86.61389249999999
‘ppt, 0.03182338405118302, 62.794445, 86.623613
‘ppt, 0.028673239147904643, 62.794445, 86.6097225
‘ppt, 0.028140186829134536, 62.794445, 86.595832
‘ppt, 0.02807651768898463, 62.794445, 86.59305699999999
‘ppt, 0.027992819553223896, 62.794445, 86.59028
‘ppt, 0.08180734528368398, 62.794445, 86.5875
‘ppt, 0.02766684506999217, 62.794445, 86.584723
‘ppt, 0.002940879359895489, 62.794445, 86.522225
```

Figure S31: Lambda values from MaxEnt model calibration, showing fitted feature weights and regularization parameters for fold 3.

```
(landcover=10.0), 0.16426678677893747, 0.0, 1.0
(landcover=11.0), 0.2607772903814948, 0.0, 1.0
(landcover=20.0), 1.0465007916781564, 0.0, 1.0
(landcover=30.0), 0.0784212004297826, 0.0, 1.0
(landcover=100.0), 0.030191261782280942, 0.0, 1.0
(landcover=130.0), 2.109550308083431, 0.0, 1.0
(landcover=190.0), 1.8587946881795174, 0.0, 1.0
(landuse=0.0), 0.0, 0.0, 1.0
(landuse=1.0), -1.897392601646248, 0.0, 1.0
(landuse=2.0), 0.11129862425189624, 0.0, 1.0
(landuse=6.0), -0.7189462567329691, 0.0, 1.0
(landuse=7.0), 0.20386433277971655, 0.0, 1.0
(landuse=9.0), 0.5623388393158485, 0.0, 1.0
(landuse=10.0), 0.009972888407174633, 0.0, 1.0
ndvi, 0.0, -0.11309412, 0.8416913
ppt, 0.0, 62.794445, 216.23611
soil, 4.226945444883093, 22.041666, 440.89166
tavg, 1.5184537298885628, 20.402779, 29.20139
ndvi^2, -0.7778624392158132, 1.0707767968710759E-5, 0.7084442444956901
ppt^2, -0.7464400023400066, 3943.142322858025, 46758.0552679321
‘ppt, 0.30817390637969144, 62.794445, 73.834723
‘ppt, 0.2611088949666702, 62.794445, 84.90139
‘ppt, 0.40823601455861386, 62.794445, 85.57916700000001
‘soil, -0.006161232556129105, 22.041666, 208.401385
‘soil, 2.004951063064651, 22.041666, 64.96111300000001
‘ppt, 0.15527439745421923, 62.794445, 86.54583299999999
‘ppt, 0.11943738932479474, 62.794445, 86.5124975
‘ppt, 0.090141436916629, 62.794445, 86.32083
‘soil, -0.2959556436841056, 22.041666, 265.99306
’ndvi, -0.3783879602294137, 0.5374942, 0.8416913
‘ppt, 0.07951411665234911, 62.794445, 86.459723
‘ppt, 0.09483127712933839, 62.794445, 86.30139
‘ppt, 0.09805932822221704, 62.794445, 86.31805299999999
‘ppt, 0.11529549430999855, 62.794445, 86.27639
‘ppt, 0.19662111637500496, 62.794445, 73.57639
’tavg, 0.023277496576414577, 28.509026499999997, 29.20139
’tavg, 0.06207250151635624, 28.507637, 29.20139
’tavg, 0.28878757509339903, 28.506248499999998, 29.20139
‘ppt, 0.12852741399838882, 62.794445, 73.5
‘ppt, 0.8569699286398487, 62.794445, 85.9875025
‘ppt, 0.2563785839083674, 62.794445, 85.9847225
’tavg, 0.055032107563950626, 28.50486, 29.20139
’ndvi, -0.5962141119487957, 0.5480188349999999, 0.8416913
’tavg, 0.3475314230959901, 28.5034715, 29.20139
’tavg, 0.08254811747332523, 28.5006945, 29.20139
‘ppt, 0.008733922491118602, 62.794445, 85.895837
‘ppt, 0.007683027864357765, 62.794445, 85.82638700000001
’tavg, 0.01079923856643578, 28.4993055, 29.20139
‘ppt, 0.003961870907382651, 62.794445, 85.83194800000001
‘ppt, 0.019786250256632328, 62.794445, 85.815275
‘ppt, 0.04971346330085863, 62.794445, 85.809722
‘ppt, 0.018952898455070068, 62.794445, 85.80694700000001
‘ppt, 0.0293878113930533, 62.794445, 85.80417
‘ppt, 0.01952380904141622, 62.794445, 85.800003
‘ppt, 0.02599946727392271, 62.794445, 85.754167
linearPredictorNormalizer, 6.198210491863461
densityNormalizer, 429.4940622473078
numBackgroundPoints, 21117
entropy, 9.12739459877354
```

Figure S32: Lambda values from MaxEnt model calibration, showing fitted feature weights and regularization parameters for fold 4.

```
(landcover=10.0), 0.16490399567151345, 0.0, 1.0
(landcover=11.0), 0.16135471187535694, 0.0, 1.0
(landcover=20.0), 0.9813094209983253, 0.0, 1.0
(landcover=30.0), 0.176275572840984, 0.0, 1.0
(landcover=100.0), 0.06578846790216054, 0.0, 1.0
(landcover=190.0), 1.2752907671251574, 0.0, 1.0
(landuse=0.0), 0.0, 0.0, 1.0
(landuse=1.0), -1.4619389519259034, 0.0, 1.0
(landuse=2.0), 0.8026416023104622, 0.0, 1.0
(landuse=7.0), 0.9696871232795087, 0.0, 1.0
(landuse=9.0), 1.0604051503975642, 0.0, 1.0
ndvi, 0.0, -0.11309412, 0.8416913
ppt, 0.0, 62.794445, 216.23611
soil, 3.7076748660688454, 22.041666, 440.89166
tavg, 1.0827020805940362, 20.402779, 29.20139
ndvi^2, -1.7593771808369676, 1.0707767968710759E-5, 0.7084442444956901
ppt^2, -0.05359486627975257, 3943.142322858025, 46758.0552679321
‘ppt, 1.4596279795038225, 62.794445, 73.584723
’tavg, 0.60393059098485, 28.415971499999998, 29.20139
’tavg, 0.17492154670538224, 28.9381945, 29.20139
‘soil, 1.1981177241838503, 22.041666, 65.929165
’tavg, 0.1846067233312734, 28.935416, 29.20139
’tavg, 0.34376219117330786, 28.932638, 29.20139
‘ppt, 0.20306341841743694, 62.794445, 86.655555
‘ppt, 0.2604392685124369, 62.794445, 86.729163
‘ppt, 0.12825534830846014, 62.794445, 86.683335
‘ppt, 0.3057264520438568, 62.794445, 86.61805799999999
‘ppt, 0.03695594496653008, 62.794445, 86.80139
’tavg, 0.3335593283307052, 28.929859999999998, 29.20139
‘ppt, 0.16895867590880284, 62.794445, 86.75694200000001
‘ppt, 0.18470138605865033, 62.794445, 86.75277700000001
‘ppt, 0.085021272224 7119, 62.794445, 86.73194
‘ppt, 0.07283907798743465, 62.794445, 73.1875
‘ppt, 0.08184976228954795, 62.794445, 73.20139
‘ppt, 0.04641904682302602, 62.794445, 86.68888799999999
‘ppt, 0.020605947444318484, 62.794445, 86.65972
‘ppt, 0.022921919478947436, 62.794445, 73.226388
‘ppt, 0.03573614958387685, 62.794445, 73.23611
’tavg, 0.06959095543829133, 28.411804, 29.20139
‘ppt, 0.020299038798902436, 62.794445, 86.67917
‘ppt, 0.0414923286922127, 62.794445, 86.67361299999999
‘ppt, 0.023844486076280685, 62.794445, 86.67083299999999
‘ppt, 0.022520311550812605, 62.794445, 86.66805199999999
‘ppt, 0.021780480725240057, 62.794445, 86.6625
‘ppt, 0.22554202118478253, 62.794445, 86.650002
‘ppt, 0.1087859554913192, 62.794445, 86.644447
‘ppt, 0.021277019679888008, 62.794445, 86.63889
‘ppt, 0.03193709198641275, 62.794445, 73.24305799999999
‘ppt, 0.01565216341514486, 62.794445, 86.633332
‘ppt, 0.01560196296617856, 62.794445, 86.629167
‘ppt, 0.06052867650579081, 62.794445, 86.61389249999999
‘ppt, 0.03182338405118302, 62.794445, 86.623613
‘ppt, 0.028673239147904643, 62.794445, 86.6097225
‘ppt, 0.028140186829134536, 62.794445, 86.595832
‘ppt, 0.02807651768898463, 62.794445, 86.59305699999999
‘ppt, 0.027992819553223896, 62.794445, 86.59028
‘ppt, 0.08180734528368398, 62.794445, 86.5875
‘ppt, 0.02766684506999217, 62.794445, 86.584723
‘ppt, 0.002940879359895489, 62.794445, 86.522225
```

Figure S33: Lambda values from MaxEnt model calibration, showing fitted feature weights and regularization parameters for fold 5.

```
(landcover=10.0), 0.3315964994041709, 0.0, 1.0
(landcover=11.0), 0.24474595305131572, 0.0, 1.0
(landcover=20.0), 0.9027562084991272, 0.0, 1.0
(landcover=30.0), 0.14184788090197087, 0.0, 1.0
(landcover=100.0), 0.02978520917730976, 0.0, 1.0
(landcover=130.0), 2.0637317704494125, 0.0, 1.0
(landcover=190.0), 1.1349636210981726, 0.0, 1.0
(landuse=0.0), 0.1519650443596067, 0.0, 1.0
(landuse=1.0), -1.9104135323619678, 0.0, 1.0
(landuse=2.0), 0.16204721589288332, 0.0, 1.0
(landuse=6.0), -0.39386164828082226, 0.0, 1.0
(landuse=7.0), 0.5090753148829801, 0.0, 1.0
(landuse=9.0), 0.20701221779185333, 0.0, 1.0
ndvi, 0.0, -0.11309412, 0.8416913
ppt, 0.0, 62.794445, 216.23611
soil, 4.683600135117185, 22.041666, 440.89166
tavg, 1.3988939295673142, 20.402779, 29.20139
ndvi^2, -1.3642061365450115, 1.0707767968710759E-5, 0.7084442444956901
ppt^2, -0.9017075802757649, 3943.142322858025, 46758.0552679321
‘ppt, 0.6390022336384148, 62.794445, 74.04305199999999
‘ppt, 1.3325403979503099, 62.794445, 84.440278
’tavg, 1.1179741410833963, 28.7298605, 29.20139
‘ppt, 0.47217552620898473, 62.794445, 84.866668
’ndvi, -0.5016379976349253, 0.5624717, 0.8416913
‘soil, 0.38314672389592863, 22.041666, 65.31388799999999
‘ppt, 0.16279048498863413, 62.794445, 85.9847225
‘ppt, 0.1821004418261383, 62.794445, 85.94583
‘soil, 0.19045458461460857, 22.041666, 65.033333
‘soil, 0.19417186972390288, 22.041666, 65.10694
‘soil, 0.18361614304286245, 22.041666, 65.184722
‘ppt, 0.060163652709311455, 62.794445, 85.70416700000001
‘soil, 0.009587544634766397, 22.041666, 65.001387
‘soil, 0.1342088193741025, 22.041666, 65.18888799999999
‘ppt, 0.10378492735854429, 62.794445, 85.4125
‘soil, 0.13866910481193098, 22.041666, 65.455555
‘soil, 0.12183320623171423, 22.041666, 65.683335
‘soil, 0.14461900829456473, 22.041666, 66.1347195
‘ppt, 0.07270826535119448, 62.794445, 85.49305799999999
‘soil, 0.07427084215117563, 22.041666, 65.872225
‘soil, 0.1371685237642104, 22.041666, 66.14166750000001
‘soil, 0.056681347360408516, 22.041666, 65.82361
‘ppt, 0.10111470953664019, 62.794445, 85.3874975
‘soil, 0.0961668844706637, 22.041666, 65.715277
‘soil, 0.05760019446934292, 22.041666, 65.72361000000001
‘soil, 0.08773697548535916, 22.041666, 65.80278
‘ppt, 0.04842334993416285, 62.794445, 85.3597225
‘ppt, 0.011988145631282233, 62.794445, 85.354165
‘ppt, 0.19197895857670352, 62.794445, 85.2875
’tavg, 0.029111375151571094, 28.736805, 29.20139
’tavg, 0.05175883915267326, 28.735416, 29.20139
‘ppt, 0.04060499271917148, 62.794445, 85.27639
‘ppt, 0.010860504525859032, 62.794445, 85.28194300000001
‘ppt, 0.03120238169432654, 62.794445, 85.28472
‘ppt, 0.013536324777619256, 62.794445, 73.830555
‘ppt, 0.006138102020973551, 62.794445, 85.273612
linearPredictorNormalizer, 6.522333900116794
densityNormalizer, 329.5477543906968
numBackgroundPoints, 21117
entropy, 9.20535854963109
```

Figure S34: Lambda values from MaxEnt model calibration, showing fitted feature weights and regularization parameters for fold 6.

```
(landcover=10.0), 0.6840238241981481, 0.0, 1.0
(landcover=20.0), 1.0798764504608465, 0.0, 1.0
(landcover=100.0), 0.1793831583532885, 0.0, 1.0
(landcover=130.0), 2.3090092520884773, 0.0, 1.0
(landcover=190.0), 2.0290298844936725, 0.0, 1.0
(landuse=0.0), 0.028944927562298606, 0.0, 1.0
(landuse=1.0), -1.7967727278398702, 0.0, 1.0
(landuse=2.0), 0.2946166485484663, 0.0, 1.0
(landuse=6.0), -0.407206770926344, 0.0, 1.0
(landuse=7.0), 0.2448884574179401, 0.0, 1.0
(landuse=9.0), 0.2410029830943555, 0.0, 1.0
ndvi, 0.0, -0.11309412, 0.8416913
ppt, 0.0, 62.794445, 216.23611
soil, 2.963532966508948, 22.041666, 440.89166
tavg, 1.756281885105557, 20.402779, 29.20139
ndvi^2, -2.560066626951634, 1.0707767968710759E-5, 0.7084442444956901
ppt^2, -6.197076788459777, 3943.142322858025, 46758.0552679321
soil^2, 4.263182755290321, 485.83504005555596, 194385.45585755561
`ppt, 0.2364327112713367, 62.794445, 85.220832
`ppt, 0.22184348262974127, 62.794445, 85.34861
`ppt, 1.5199877910504946, 62.794445, 85.52639
`ppt, 0.538880875778924, 62.794445, 73.24305799999999
'tavg, 1.228182467502584, 28.9798605, 29.20139
`soil, 0.5097909125919192, 22.041666, 64.10833249999999
`tavg, -0.10539702336917492, 20.402779, 26.7854175
`soil, 0.3770565499780094, 22.041666, 64.23889249999999
`soil, 0.2861015662946041, 22.041666, 64.28472
`soil, 0.34872343792571203, 22.041666, 64.4625
`ppt, 0.06527379340691197, 62.794445, 85.95416700000001
`ppt, 0.05444127470210688, 62.794445, 73.601388
`ppt, 0.05378328144016927, 62.794445, 85.87777700000001
'tavg, 0.1493430491967708, 28.970138499999997, 29.20139
`soil, 0.05518803771579149, 22.041666, 64.369445
`ppt, 0.05282864118010885, 62.794445, 73.63889
`ppt, 0.04130830884518182, 62.794445, 85.754167
`ppt, 0.02135372371112203, 62.794445, 85.76389
`soil, 0.23931139153509778, 22.041666, 64.43888799999999
`ppt, 0.08693029839627761, 62.794445, 73.64306
`ppt, 0.04563876794931459, 62.794445, 85.78194500000001
`soil, 0.07075065623453951, 22.041666, 64.434722
`ppt, 0.06722026847212655, 62.794445, 85.79583299999999
'tavg, 0.11746434655669621, 28.428470500000003, 29.20139
`ppt, 0.024003529213660165, 62.794445, 85.800003
`soil, 0.013186243792451301, 22.041666, 64.599998
'tavg, 0.22714151441889693, 28.429859, 29.20139
`ppt, 0.03819324879372394, 62.794445, 73.647225
`ppt, 0.005503596458047744, 62.794445, 85.80694700000001
`ppt, 0.005118847751974708, 62.794445, 85.80417
linearPredictorNormalizer, 5.617753198119896
densityNormalizer, 468.4188809634816
numBackgroundPoints, 21117
entropy, 8.978551844387004
```

Figure S35: Lambda values from MaxEnt model calibration, showing fitted feature weights and regularization parameters for fold 7.

```
(landcover=10.0), 0.0, 0.0, 1.0
(landcover=11.0), 0.08813922137421229, 0.0, 1.0
(landcover=20.0), 0.7762823182009897, 0.0, 1.0
(landcover=100.0), 0.10085596799356311, 0.0, 1.0
(landcover=130.0), 1.8671380528572272, 0.0, 1.0
(landcover=190.0), 1.0724651462521984, 0.0, 1.0
(landuse=0.0), 0.0, 0.0, 1.0
(landuse=1.0), -1.644167341491408, 0.0, 1.0
(landuse=2.0), 0.40847804772380797, 0.0, 1.0
(landuse=6.0), -0.6458741940365968, 0.0, 1.0
(landuse=7.0), 0.5883053624441298, 0.0, 1.0
(landuse=9.0), 0.2793440481126119, 0.0, 1.0
ndvi, 0.0, -0.11309412, 0.8416913
ppt, 0.0, 62.794445, 216.23611
soil, 3.0076467293023095, 22.041666, 440.89166
tavg, 2.4555411705978285, 20.402779, 29.20139
ndvi^2, -2.0075556827012835, 1.0707767968710759E-5, 0.7084442444956901
ppt^2, -0.45817437827737034, 3943.142322858025, 46758.0552679321
‘soil, -0.026878674592704276, 22.041666, 208.401385
‘soil, 0.4818243040536843, 22.041666, 67.84722199999999
‘ppt, 0.20034546564504627, 62.794445, 86.24305799999999
‘ppt, 0.1513905322294633, 62.794445, 86.34305699999999
‘soil, 0.21393004354815662, 22.041666, 67.78611000000001
‘ppt, 0.5123306996046488, 62.794445, 86.3874975
’tavg, 0.22693307836653007, 28.943748499999998, 29.20139
‘soil, 0.14482264309494722, 22.041666, 68.106945
‘ppt, 0.05549108508615938, 62.794445, 86.67083299999999
‘soil, 0.1407901941124693, 22.041666, 68.037497
‘ppt, 0.08122247364965272, 62.794445, 86.5152775
‘ppt, 0.05796379922052087, 62.794445, 86.5124975
’tavg, 0.12447721834789656, 28.9381945, 29.20139
’tavg, 0.1261364660957071, 28.935416, 29.20139
‘soil, 0.05762198010575233, 22.041666, 67.62361
‘ppt, 0.04486976802289269, 62.794445, 86.54583299999999
‘soil, 0.24081227270207914, 22.041666, 67.654165
’tavg, 0.339468031143571, 28.9368055, 29.20139
‘ppt, 0.02009819675537436, 62.794445, 86.469445
’tavg, 0.055939594553212624, 28.452084, 29.20139
‘ppt, 0.4229570132829168, 62.794445, 86.46528
‘ppt, 0.04760331503528179, 62.794445, 86.4625
‘ppt, 0.0999815236978382, 62.794445, 86.459723
‘ppt, 0.047272295605110155, 62.794445, 86.45694800000001
‘ppt, 0.03558733763146757, 62.794445, 86.383332
‘ppt, 0.03548158706082484, 62.794445, 86.379167
‘ppt, 0.03536897821919931, 62.794445, 86.37639
‘ppt, 0.1513945287232551, 62.794445, 86.37361
‘ppt, 0.0347676194217428, 62.794445, 86.370833
‘ppt, 0.1494291893222136, 62.794445, 86.32638700000001
‘ppt, 0.1083714246493821, 62.794445, 86.32361
‘ppt, 0.07060101960371422, 62.794445, 86.31805299999999
‘ppt, 0.03334638672811109, 62.794445, 86.31388799999999
‘soil, 0.11853130950445011, 22.041666, 67.309725
’tavg, 0.4790006797544473, 28.453471999999998, 29.20139
‘soil, -0.4649238609449497, 22.041666, 208.390275
‘ppt, 0.12305260851792235, 62.794445, 86.279168
‘soil, 0.09028780125111817, 22.041666, 67.320832
‘ppt, 0.05273521227422704, 62.794445, 86.30139
‘ppt, 0.09963475788039551, 62.794445, 86.294445
‘ppt, 0.04705529333732725, 62.794445, 86.2875
```

Figure S36: Lambda values from MaxEnt model calibration, showing fitted feature weights and regularization parameters for fold 8.

```
(landcover=10.0), 0.16847522654355984, 0.0, 1.0
(landcover=11.0), 0.25316476596444276, 0.0, 1.0
(landcover=20.0), 0.6941473425326736, 0.0, 1.0
(landcover=30.0), 0.30619126297043436, 0.0, 1.0
(landcover=100.0), 0.105543390588136, 0.0, 1.0
(landcover=130.0), 1.8223171679342214, 0.0, 1.0
(landcover=190.0), 1.3748529135201126, 0.0, 1.0
(landuse=0.0), 0.0, 0.0, 1.0
(landuse=1.0), -1.6968924342250002, 0.0, 1.0
(landuse=2.0), 0.04723105867248056, 0.0, 1.0
(landuse=6.0), -0.3912303838628079, 0.0, 1.0
(landuse=7.0), 0.28265441511633377, 0.0, 1.0
(landuse=9.0), 0.6899924738661993, 0.0, 1.0
(landuse=10.0), 0.19961311764182013, 0.0, 1.0
ndvi, 0.0, -0.11309412, 0.8416913
ppt, 0.0, 62.794445, 216.23611
soil, 4.25471645677768, 22.041666, 440.89166
tavg, 0.0, 20.402779, 29.20139
ndvi^2, -2.222911773054531, 1.0707767968710759E-5, 0.7084442444956901
ppt^2, -0.36408500877424615, 3943.142322858025, 46758.0552679321
‘ppt, 1.230894959226757, 62.794445, 85.166665
’tavg, 1.4132224269721592, 28.510416, 29.20139
’ndvi, -0.11031848351546522, 0.548829705, 0.8416913
‘soil, 0.4129299299040643, 22.041666, 64.415275
’ndvi, -1.1701666929334271, 0.5480188349999999, 0.8416913
‘ppt, 0.38925341117283146, 62.794445, 86.074997
‘ppt, 0.3359519370488543, 62.794445, 86.370833
’tavg, 0.18337385619833357, 28.94236, 29.20139
‘tavg, -1.0887631120740997, 20.402779, 26.7854175
‘ppt, 0.1610410166423369, 62.794445, 86.59305699999999
‘soil, 0.21997162750546953, 22.041666, 64.4625
‘ppt, 0.1192083607655725, 62.794445, 86.72361
‘soil, 0.2669216405167662, 22.041666, 64.540275
‘soil, -0.4728189098605978, 22.041666, 265.99306
‘ppt, 0.12040188792675967, 62.794445, 86.708335
’tavg, 0.22237408744069814, 28.935416, 29.20139
‘soil, 0.13288748898369224, 22.041666, 64.66388699999999
‘ppt, 0.1436994674746559, 62.794445, 86.469445
‘soil, 0.18811012025682308, 22.041666, 65.040275
‘ppt, 0.09905493455184475, 62.794445, 86.46528
‘ppt, 0.12990718084214306, 62.794445, 86.5152775
‘soil, 0.15104964620884753, 22.041666, 65.27638999999999
‘soil, 0.1825528295785084, 22.041666, 65.516667
‘soil, 0.18612733536657772, 22.041666, 65.58194800000001
‘soil, 0.19150877195210897, 22.041666, 65.80278
‘ppt, 0.16404925893444197, 62.794445, 86.3944425
‘ppt, 0.1457554649938111, 62.794445, 73.595832
‘ppt, 0.12746461525816152, 62.794445, 86.3874975
‘ppt, 0.0634342087232024, 62.794445, 86.379167
’tavg, 0.0692741301518136, 28.513194499999997, 29.20139
’tavg, 0.0886562928383869, 28.9340265, 29.20139
‘ppt, 0.035959512317229055, 62.794445, 86.25972
‘ppt, 0.018710710272391805, 62.794445, 86.25694200000001
‘ppt, 0.05704778170871349, 62.794445, 86.28194300000001
‘ppt, 0.052816299694199105, 62.794445, 86.155555
‘ppt, 0.20177077892879186, 62.794445, 73.63472
‘ppt, 0.01621067898957928, 62.794445, 86.20138700000001
‘ppt, 0.01601299088505974, 62.794445, 86.205555
‘ppt, 0.015640407709263813, 62.794445, 86.19861
```

Figure S37: Lambda values from MaxEnt model calibration, showing fitted feature weights and regularization parameters for fold 9.

(landcover=10.0), 0.3418220503904655, 0.0, 1.0
(landcover=11.0), 0.2946154884978888, 0.0, 1.0
(landcover=20.0), 0.8644802814608451, 0.0, 1.0
(landcover=30.0), 0.16133058258410177, 0.0, 1.0
(landcover=100.0), 0.049676357610148644, 0.0, 1.0
(landcover=130.0), 2.107048233355934, 0.0, 1.0
(landcover=190.0), 1.3274572776432965, 0.0, 1.0
(landuse=0.0), 0.0, 0.0, 1.0
(landuse=1.0), -1.920291810307604, 0.0, 1.0
(landuse=2.0), 0.10333890499402709, 0.0, 1.0
(landuse=6.0), -0.7008625883585617, 0.0, 1.0
(landuse=7.0), 0.46846065945592535, 0.0, 1.0
(landuse=9.0), 0.5910916127776392, 0.0, 1.0
ndvi, 0.0, -0.11309412, 0.8416913
ppt, 0.0, 62.794445, 216.23611
soil, 4.8801689639829835, 22.041666, 440.89166
tavg, 1.6935917379016103, 20.402779, 29.20139
ndvi^2, -2.25032489297961, 1.0707767968710759E-5, 0.7084442444956901
ppt^2, -1.2316945645057555, 3943.142322858025, 46758.0552679321
‘ppt, 0.745597578676201, 62.794445, 84.930557
’tavg, 1.155811313751701, 28.7770825, 29.20139
‘ppt, 0.599065907373327, 62.794445, 85.14306
‘ppt, 0.47077317748871444, 62.794445, 85.61528
‘ppt, 0.30873399543196967, 62.794445, 85.754167
‘soil, 0.26093108115026015, 22.041666, 64.809725
‘soil, 0.1146999033364137, 22.041666, 64.540275
‘ppt, 0.08346873839884271, 62.794445, 86.3874975
‘soil, 0.15990851936726103, 22.041666, 64.497223
‘soil, 0.2578731989627407, 22.041666, 64.4625
‘ppt, 0.18548374984232527, 62.794445, 86.42639
‘soil, 0.2431503220375072, 22.041666, 65.033333
‘ppt, 0.14037802494250856, 62.794445, 86.31805299999999
‘soil, 0.12680647298230555, 22.041666, 64.8
‘soil, 0.1371632433226378, 22.041666, 65.370833
‘soil, 0.23813973844344064, 22.041666, 65.18888799999999
‘ppt, 0.07721816363335905, 62.794445, 86.25694200000001
‘soil, 0.2705581145108057, 22.041666, 65.31388799999999
‘ppt, 0.0974302651538357, 62.794445, 85.9875025
‘soil, 0.05443138954544969, 22.041666, 65.040275
’tavg, 0.12004983925923472, 28.935416, 29.20139
‘ppt, 0.039686861638515133, 62.794445, 73.5
‘ppt, 0.056708834009999216, 62.794445, 73.5125
’tavg, 0.15508194663369418, 28.9340265, 29.20139
‘ppt, 0.015779820049567777, 62.794445, 85.95416700000001
‘soil, 0.062397045348956286, 22.041666, 65.10694
‘ppt, 0.024665755475666906, 62.794445, 85.96528
‘ppt, 0.017617651168734046, 62.794445, 85.940278
‘ppt, 0.025703552860671584, 62.794445, 73.519447
‘ppt, 0.10120925319936147, 62.794445, 85.9375
‘ppt, 0.043388318083984065, 62.794445, 85.934722
‘ppt, 0.021232485959391048, 62.794445, 73.540277
‘ppt, 0.08324677824319823, 62.794445, 73.54861299999999
’tavg, 0.029763007865681534, 28.7881925, 29.20139
’tavg, 0.08453815468634318, 28.789582, 29.20139
‘ppt, 0.019224126004181163, 62.794445, 85.92083199999999
‘ppt, 0.12176564822954383, 62.794445, 85.91111000000001
‘ppt, 0.03813748110679496, 62.794445, 85.904168
‘ppt, 0.07679205193707915, 62.794445, 73.55278
‘ppt, 0.03783366152815475, 62.794445, 85.900002
